# Malaria chemoprevention with monthly dihydroartemisinin-piperaquine for the post-discharge management of severe anaemia in children aged less than 5 years in Uganda and Kenya: study protocol for a multi-centre, two-arm, randomised, placebo-controlled, superiority trial

**DOI:** 10.1186/s13063-018-2972-1

**Published:** 2018-11-06

**Authors:** Titus K. Kwambai, Aggrey Dhabangi, Richard Idro, Robert Opoka, Simon Kariuki, Aaron M. Samuels, Meghna Desai, Michael Boele van Hensbroek, Chandy C. John, Bjarne Robberstad, Duolao Wang, Kamija Phiri, Feiko O. ter Kuile

**Affiliations:** 1 Kenya Medical Research Institute (KEMRI), Centre for Global Health Research (CGHR), PO Box 1578, Kisumu, 40100 Kenya; 2 Kisumu County Department of Health, Kenya Ministry of Health, Kisumu, Kenya; 3 Department of Clinical Sciences, Liverpool School of Tropical Medicine (LSTM), Liverpool, UK; 4 Makerere University College of Health Sciences, Kampala, Uganda; 5 Malaria Branch, Division of Parasitic Diseases and Malaria, Centers for Disease Control and Prevention (CDC), Atlanta, GA USA; 6 Department of Global Child Health, Emma Children’s Hospital Academic Medical Centre, University of Amsterdam, Amsterdam, the Netherlands; 7 Ryan White Center for Pediatric Infectious Disease and Global Health, Indiana University School of Medicine, Indianapolis, IN USA; 8 Centre for International Health, Department of Global Public Health and Primary Care, University of Bergen, Bergen, Norway; 9 College of Medicine, University of Malawi, Blantyre, Malawi

**Keywords:** Malaria, Severe anaemia, Chemoprevention, Post-discharge, Readmission, Mortality, Dihydroartemisinin-piperaquine, Protocol, Cost-effectiveness

## Abstract

**Background:**

Children hospitalised with severe anaemia in malaria endemic areas in Africa are at high risk of readmission or death within 6 months post-discharge. Currently, no strategy specifically addresses this period. In Malawi, 3 months of post-discharge malaria chemoprevention (PMC) with monthly treatment courses of artemether-lumefantrine given at discharge and at 1 and 2 months prevented 30% of all-cause readmissions by 6 months post-discharge. Another efficacy trial is needed before a policy of malaria chemoprevention can be considered for the post-discharge management of severe anaemia in children under 5 years of age living in malaria endemic areas.

**Objective:**

We aim to determine if 3 months of PMC with monthly 3-day treatment courses of dihydroartemisinin-piperaquine is safe and superior to a single 3-day treatment course with artemether-lumefantrine provided as part of standard in-hospital care in reducing all-cause readmissions and deaths (composite primary endpoint) by 6 months in the post-discharge management of children less than 5 years of age admitted with severe anaemia of any or undetermined cause.

**Methods/design:**

This is a multi-centre, two-arm, placebo-controlled, individually randomised trial in children under 5 years of age recently discharged following management for severe anaemia. Children in both arms will receive standard in-hospital care for severe anaemia and a 3-day course of artemether-lumefantrine at discharge. At 2 weeks after discharge, surviving children will be randomised to receive either 3-day courses of dihydroartemisinin-piperaquine at 2, 6 and 10 weeks or an identical placebo and followed for 26 weeks through passive case detection. The trial will be conducted in hospitals in malaria endemic areas in Kenya and Uganda. The study is designed to detect a 25% reduction in the incidence of all-cause readmissions or death (composite primary outcome) from 1152 to 864 per 1000 child years (power 80%, α = 0.05) and requires 520 children per arm (1040 total children).

**Results:**

Participant recruitment started in May 2016 and is ongoing.

**Trial registration:**

ClinicalTrials.gov, NCT02671175. Registered on 28 January 2016.

**Electronic supplementary material:**

The online version of this article (10.1186/s13063-018-2972-1) contains supplementary material, which is available to authorized users.

## Background

Severe anaemia, defined as haemoglobin (Hb) concentration level below 5.0 g/dL or haematocrit below 15.0% [1], is a major public health problem in low and middle-income countries. Severe anaemia is associated with approximately one third of hospital admissions among febrile children in sub-Saharan Africa, contributing substantially to paediatric morbidity and mortality, especially in malaria endemic areas [2, 3]. Children under 5 years of age are most vulnerable to the long-term effects of severe anaemia, including decreased cognitive performance and mental and motor development [4]. Rates of in-hospital mortality due to severe anaemia ranging from 4 to 12% have been reported in different epidemiological settings [5–7]. In addition, these reports indicate a high post-discharge mortality and morbidity, especially in the first 3 to 6 months. Longitudinal follow-up of children aged less than 5 years admitted with severe anaemia in Malawi showed that 8.2% died by 6 months post-discharge and 5.9% were readmitted with severe anaemia, compared to those without severe anaemia, among whom 1.6% died and 0.5% were readmitted [8, 9]. Similar high rates of post-discharge mortality (10% by 8 weeks) were observed in malaria endemic areas of western Kenya [10] and in Uganda, where 12% died or were readmitted within 6 months [11].

Standard in-hospital treatment of severe anaemia in many countries in sub-Saharan Africa consists of a blood transfusion and parenteral artesunate for severe malaria [12]. For severe malarial anaemia, this is completed with a 3-day course of artemisinin-based combination therapy (ACT), usually artemether-lumefantrine. Children are often discharged with a short course of iron and folate, typically with no further scheduled follow-up. Haematological recovery from malaria-associated anaemia takes at least 6 weeks [13, 14]. However, many children in these areas experience episodes of new or recrudescent malaria infections after discharge. These infections negate the initial rise in haemoglobin achieved by blood transfusion, resulting in delayed haematological recovery and potential rebound of severe anaemia and death in some children [10, 15–17]. Furthermore, delayed haemolytic anaemia occurring 1 to 3 weeks after artesunate treatment of falciparum malaria has been reported in non-immune travellers [18, 19], although more recent studies show this to be rare in African children [20].

Malaria control strategies in endemic and epidemic-prone areas include intermittent preventive therapy (IPT). IPT is the administration of a full treatment course using long-acting antimalarials at pre-defined time intervals irrespective of a patient’s malaria status to clear existing infections and to provide prolonged prophylaxis against new infections [21]. The World Health Organization (WHO) recommends IPT as a malaria control strategy in malaria endemic areas for pregnant women (IPTp) [22, 23], infants (IPTi) [24] and for children in areas with seasonal malaria transmission (‘seasonal malaria chemoprevention’, or SMC) [25]. Currently, no control strategy specifically addresses the high-risk post-discharge period for children previously treated for severe anaemia in malaria endemic areas. In Malawi, 3 months of malaria chemoprevention with three full treatment courses of artemether-lumefantrine, given in-hospital to children under 5 years of age admitted with severe malarial anaemia, and at 1 and 2 months post-discharge, prevented 31% of deaths or readmissions by 6 months post-discharge, and 30% of all-cause readmissions [17]. These results are consistent with earlier findings from The Gambia which showed that, in children with severe anaemia, chemoprevention targeted during the malaria transmission season halved the rate of clinical malaria and reduced all-cause hospital readmission by 78% in one trial and recurrence of severe anaemia by 78% in another [26, 27]. These data indicate that malaria chemoprevention in the post-discharge period may provide substantial health benefits.

We are conducting an efficacy trial in Kenya and Uganda to determine the efficacy and safety of 3 months of malaria chemoprevention post-discharge as a potentially cost-effective strategy to reduce all-cause readmissions and deaths in children admitted with severe anaemia. We hypothesise that, by creating a prophylactic time-window post-transfusion for malaria, more time is assured for bone marrow recovery, resulting in a more sustained haematological recovery post-discharge.

We refer to this strategy as post-discharge malaria chemoprevention (PMC) to illustrate the similarities with SMC rather than with IPT in pregnancy as it aims to provide complete, rather than intermittent prophylaxis.

## Methods/design

### Design overview

This will be a multi-centre, parallel group, two-arm, placebo-controlled, individually randomised, superiority trial with 1:1 allocation ratio comparing the safety and efficacy of three courses of monthly PMC with dihydroartemisinin-piperaquine (PMC-DP) or placebo post-discharge provided in addition to the standard single 3-day treatment course with artemether-lumefantrine given as part of routine in-hospital care (ClinicalTrials.gov, NCT02671175; registered 28 January 2016). Randomisation to PMC-DP or placebo will occur at 2 weeks after enrolment, and PMC treatments will be administered at 2, 6 and 10 weeks. The primary outcome will be the number of all-cause deaths or all-cause readmissions between 2 and 26 weeks after enrolment (composite outcome). The study will be conducted in Uganda and Kenya, using randomisation stratified by weight and study centre. The study will include a total of 1040 children (520 per study arm) less than 5 years of age who have been admitted for severe anaemia and have successfully completed the standard in-hospital treatment.

### Primary objective

The primary objective is to determine if 3 months of post-discharge malaria chemoprevention with monthly 3-day treatment courses of DP is superior to the single 3-day treatment course with artemether-lumefantrine provided as part of standard in-hospital care in reducing all-cause readmissions and deaths by 6 months in the post-discharge management of children less than 5 years of age admitted with severe anaemia.

### Secondary objectives

The secondary objectives include the determination of the safety of three courses of monthly DP and the cost-effectiveness of PMC-DP compared to the current standard of care.

### Design considerations

#### Rationale for choice of DP for PMC

Optimal antimalarial prophylaxis with maximum compliance would be provided by a regimen that is long acting so that administration is not required more frequently than monthly. Sulphadoxine, mefloquine and DP have sufficiently long half-lives to be considered [28]. However, there is high-level resistance to sulphadoxine in many parts of east and southern Africa, precluding its use for this purpose in these malaria endemic areas [29]. Both amodiaquine [30] and mefloquine are poorly tolerated, which is an important consideration when providing drugs for malaria prevention to recipients with few or no symptoms [31, 32]. DP is very effective, well tolerated and provides 4 to 5 weeks of post-treatment prophylaxis. It is therefore currently the drug of choice for use for evaluation as part of IPT and malaria chemoprevention in areas with high-grade parasite resistance to sulphadoxine [33–38]. Furthermore, recent studies show that artemether-lumefantrine and DP exert inverse selective pressure on *Plasmodium falciparum* drug sensitivity [39], suggesting that DP may be a good choice for chemoprevention in areas where artemether-lumefantrine is the first-line drug of choice for case management.

#### Why in this study population?

The primary study population involves children with severe anaemia, rather than only children with severe malarial anaemia, which was the study population in the previous trial in Malawi [17]. This is based on observational studies in Malawi, Uganda and western Kenya showing that children admitted with severe anaemia appear to be at increased risk of readmission and death regardless of whether they had evidence of malaria infection at the time of admission or not (Desai et al., unpublished observations; Richard Opoko, unpublished observations) [17]. Second, reliable diagnosis of the presence of malaria is difficult, and the differentiation between severe anaemia and severe malarial anaemia is not always feasible, as it is common practice in many hospitals in sub-Saharan Africa to start parenteral treatment with antimalarials before the laboratory diagnosis of malaria is available. Furthermore, the interpretation of malaria diagnostic tests on admission may be complicated in children who received antimalarial treatment just prior to admission [40].

#### Efficacy and effectiveness of delivery mechanisms

This current study is an efficacy trial, and each treatment course will be provided by study staff directly. The first dose of each course will be observed, and where feasible, doses on days 2 and 3 will also be given under supervision, or compliance verified by home visits or contacting caretakers by mobile phone. A separate trial, focusing on the effectiveness of different delivery mechanisms, is being conducted by our consortium members in Malawi (NCT02721420).

#### Why this composite primary outcome?

Use of clinical malaria as primary outcome would require a smaller study; however, the composite outcome is used because it is more likely to drive policy. We use a composite outcome rather than a single severe outcome, such as death, to keep sample size requirements manageable.

#### Rationale for assessment by 6 months after enrolment

The period 2–26 weeks instead of 0–26 weeks is used for the primary efficacy analysis because children will not be randomised until 2 weeks after enrolment. Prior to 2 weeks, all children, including those in the placebo arm, will receive a 3-day course of artemether-lumefantrine as part of standard in-hospital care, which will be started before discharge and completed at home after discharge. The duration of post-treatment prophylaxis in our previous trial with artemether-lumefantrine is about 2 to 3 weeks [17], and we therefore do not anticipate any differential effect between the arms until children receive their first study-specific intervention upon randomisation. The protective drug levels have waned in many children by 14 weeks (i.e. about 4 weeks after the last PMC course of DP), but we follow the children for a total of 26 weeks to capture any potential prolonged benefits or rebound effects.

### Study settings

**Fig. 1:**
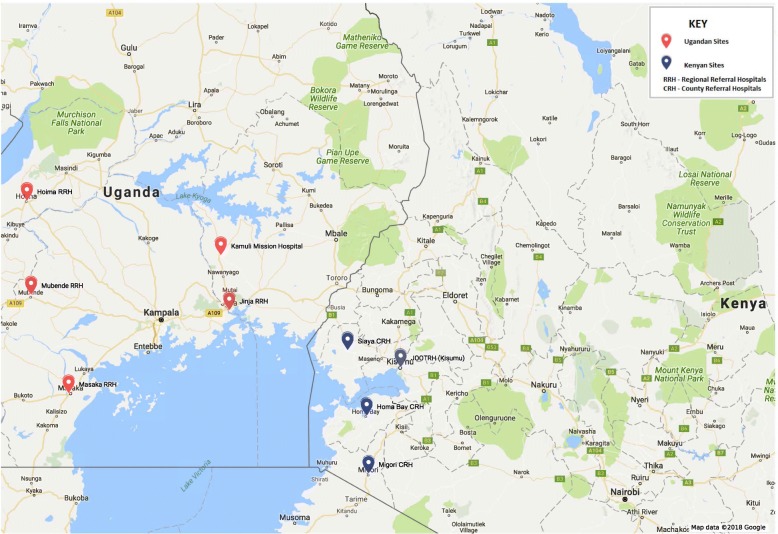
Map of study setting in Kenya and Uganda. Study sites in both western Kenya and Uganda are in the lake endemic region. These are large referral hospitals in the region with adequate diagnostic and treatment capacities for malaria and other conditions

The study will be conducted in hospitals in Kenya and Uganda located in areas with moderate to intense malaria transmission [15, 16]. The annual entomological inoculation rates vary widely. In western Kenya they range from 31.1 to 108.6 infective bites/person/year [41, 42] in areas around Kisumu and Siaya respectively, while in Uganda they range from 2.8 to 4 infective bites/person/year [43, 44] in areas around Jinja and Mubende respectively. In western Kenya, we will recruit participants from hospitals located in areas around Lake Victoria with well-documented malaria transmission intensity, including the Jaramogi Oginga Odinga Teaching & Referral Hospital (JOOTRH) and Siaya, Kisumu, Homa Bay and Migori County referral hospitals. In Uganda, we will recruit from Jinja, Hoima, Masaka and Mubende regional referral hospitals as well as Kamuli Mission Hospital (Fig. 1).

### Inclusion and exclusion criteria

The inclusion and exclusion criteria for pre-study screening, enrolment and for randomisation 2 weeks later are described in the following sections.

### Eligibility criteria

#### Eligibility criteria for pre-study screening

##### The inclusion criteria for enrolment into the pre-study screening period are as follows:

1. Haemoglobin < 5.0 g/dL or packed cell volume < 15%, or requirement for blood transfusion for other clinical reasons on or during admission to the hospital
2. Aged less than 59.5 months
3. Bodyweight ≥ 5 kg
4. Resident in catchment area

##### The exclusion criteria for enrolment into the pre-study screening period are the following:

1. Recognised specific other cause of severe anaemia, e.g. trauma, haematological malignancy, known bleeding disorder
2. Known sickle cell disease
3. Child will reside for more than 25% of the 6 months study period (i.e. 6 weeks or more) outside of catchment area

#### Eligibility criteria for enrolment into study

##### The inclusion criteria for enrolment are as follows:

1. Fulfilled the pre-study screening eligibility criteria
2. Aged less than 59.5 months
3. Clinically stable, able to take oral medication
4. Subject completed blood transfusion(s) or became clinically stable without transfusion
5. Able to feed (for breastfeeding children) or eat (for older children)
6. Provision of informed consent by parent or guardian

##### The exclusion criteria for enrolment are the following:

1. Previous enrolment in the present study
2. Known hypersensitivity to study drug
3. Use or known need at the time of enrolment for concomitant prohibited medication including drugs known to prolong the QTc interval during the 14-week PMC treatment period (Additional file 1, section 8.5.7, page 34)
4. Ongoing or planned participation in another clinical trial involving ongoing or scheduled treatment with prohibited medicinal products or active follow-up during the study
5. A known need for scheduled surgery during the subsequent course of the study
6. Anticipated non-compliance with the follow-up schedule
7. Known heart conditions, or family history of congenital prolongation of the QTc interval

#### Eligibility criteria for randomisation into study (at 2 weeks post-discharge)

##### The inclusion criteria for randomisation are as follows:

1. Fulfilled enrolment eligibility criteria and was enrolled during recent admission
2. Aged < 60 months
3. Still clinically stable, able to take oral medication, able to feed (for breastfeeding children) or eat (for older children) and able to sit unaided (for older children who were already able to do so prior to hospitalisation)

##### The exclusion criteria for randomisation are the following

1. Used DP since enrolment
2. Use or known need at the time of randomisation for concomitant prohibited medication (Additional file 1, section 8.5.7, page 34).
3. Enrolled, or known agreement to enrol into another clinical trial involving ongoing or scheduled treatment with medicinal products during the study.
4. Withdrawal of consent since enrolment

### Interventions

#### Trial medication and interventions

Children will be randomised to one of the two treatment groups: DP or placebo. Children in both arms will receive standard in-hospital care and, at discharge (enrolment) a 3-day course of artemether-lumefantrine regardless of whether they were admitted with severe malarial anaemia or severe anaemia without evidence of malaria.

##### Artemether-lumefantrine

The study will use a Good manufacturing practices (GMP) formulation of artemether-lumefantrine (Coartem®, Novartis Pharmaceuticals). The recommended treatment is a six-dose regimen over a 3-day period with dosing per bodyweight following WHO dosing recommendations as provided for in the latest WHO malaria treatment guidelines [12] (see Additional file 1: Table S3 on page 29).

##### Dihydroartemisinin-piperaquine

The study will use the Eurartesim® brand of DP from Alfasigma (formerly Sigma Tau), Italy, a co-formulated tablet containing 40 mg dihydroartemisinin and 320 mg piperaquine phosphate or as 20/160 (paediatric formulation). Dosing will be per bodyweight according to the schedule recommended by the current WHO guidelines (Additional file 1: Table S4 on page 30).

##### Placebo DP

Placebos for DP will be manufactured by Alfasigma, Italy. The dosage regimen for DP-placebo will be identical in number of tablets per day and timing of the dose to that of the active DP product. The drug administration procedures will also be identical to that for the active drugs.

#### Other medication

##### Standard in-hospital and post-discharge care

Except for the full 3-day course of artemether-lumefantrine, all care provided prior to and following enrolment of the participants in the study (at convalescence) will be according to local (hospital) or national guidelines and therefore not subject to this study. Treatment for malaria in both Kenya and Uganda conforms with the current WHO malaria treatment guidelines [12], which include artemether-lumefantrine as first-line treatment for uncomplicated malaria and parenteral artesunate for severe malaria. Details of non-study-specific care provided by the hospital staff will be recorded.

##### Iron and folate supplementation

All children will receive 28 days of iron and folate supplementation at 2 weeks post-discharge as part of routine care for severe anaemia. A standardised prophylactic dose of iron supplementation (about 2 mg/kg) will be given as monotherapy or as part of the fixed-dose formulation with folic acid (see Additional file 1, section 8.5.2.7, page 31).

### Outcomes

The primary and secondary efficacy outcomes are discussed in the following sections.

#### Primary efficacy outcome

The primary efficacy outcome is the number of all-cause deaths or all-cause readmissions between 2 and 26 weeks after enrolment (composite outcome).

#### Key secondary efficacy outcomes

1. Readmission due to severe malaria (defined as any treatment with parenteral quinine or artesunate, or presence of severe anaemia and treatment with oral antimalarials) by 26 weeks from randomisation
2. Readmissions due to severe anaemia (defined as Hb < 5 g/dL or packed cell volume < 15% or requirement for blood transfusion based on other clinical indication) by 26 weeks from randomisation
3. Readmission due to severe malarial anaemia (severe anaemia plus parenteral or oral antimalarial treatment) by 26 weeks from randomisation
4. Readmission due to severe anaemia or severe malaria (composite outcome) by 26 weeks from randomisation
5. All-cause mortality by 26 weeks from randomisation
6. All-cause hospital readmission by 26 weeks from randomisation
7. Clinic visits because of smear- or malaria rapid diagnostic test (RDT)-confirmed non-severe malaria by 26 weeks from randomisation

#### Other secondary efficacy outcomes

1. Readmission due to severe malaria-specific anaemia (severe anaemia plus parenteral or oral antimalarial treatment and parasite density > 5000/μL) by 26 weeks from randomisation
2. Readmission due to severe disease other than severe anaemia and severe malaria by 26 weeks from randomisation
3. Non-severe all-cause sick-child clinic visits by 26 weeks from randomisation
4. Non-malaria sick-child clinic visits by 26 weeks from randomisation
5. Malaria infection at 26 weeks
6. Hb at 26 weeks
7. Any anaemia (Hb < 11 g/dL), mild anaemia (Hb 8.0–10.99 g/dL), moderate anaemia (Hb 5.0–7.99 g/dL) and severe anaemia (Hb < 5 g/dL) at 26 weeks
8. Weight-for-age, height-for-age and height-for-weight Z-scores (standard deviation [SD] scores of reference population) at 26 weeks

#### Tolerability and safety outcomes

1. Serious adverse events, excluding primary and secondary efficacy outcomes, by 26 weeks from randomisation
2. Serious adverse events within 7 days after the start of each course of PMC, excluding primary and secondary efficacy outcomes
3. Adverse events by 26 weeks from randomisation
4. Adverse events within 7 days after the start of each course of PMC
5. QTc prolongation measured by electrocardiogram (ECG) 4–6 h after third dose of each course (in a subset of patients)

### Participant timeline

#### Overview of study phases and scheduled visits

The study timelines consist of an in-patient pre-study screening period while the patient is acutely ill (visit 1), followed by a screening, consent and enrolment visit (visit 2). During the convalescence phase in the hospital, patients receive artemether-lumefantrine (visit 3) prior to discharge. The patient returns to the study clinic 14 days later (visit 4) for randomisation. Home treatment visits are made at 6 (visit 5) and 10 (visit 6) weeks. The PMC period starts at 2 weeks and ends at 14 weeks, but participants receive passive follow-up for an additional 12 weeks and are then seen at 26 weeks (visit 7) for an end-of-study assessment (see Additional file 2, Fig. 2 and Additional file 1, section 8.7, pages 37 to 40)

**Fig. 2:**
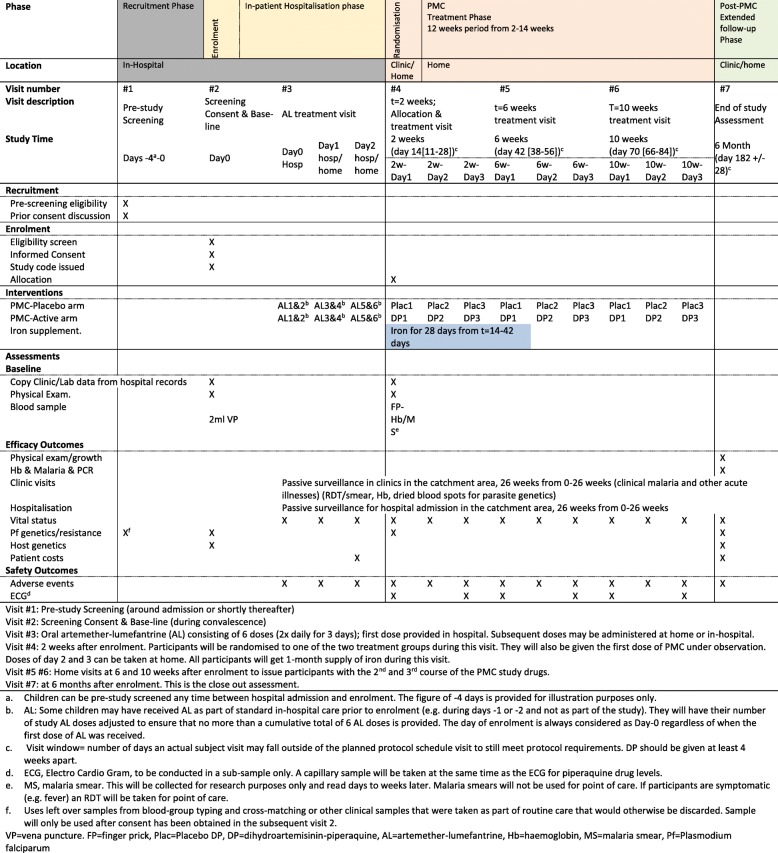
Study Design and Schedule of Assessment (Spirit figure)

.

#### Unscheduled visits (passive follow-up)

A passive surveillance system is in place to monitor intercurrent illnesses during the observation period. Parents are instructed to bring their child to the study clinic for any suspected illness. Blood samples for Hb, malaria diagnosis (RDT and smear) and filter paper dry blood spot (DBS) for parasite genetics are obtained. Verbal autopsy is conducted for children who die at home during the follow-up period. Adverse events and vital status are assessed during all scheduled or unscheduled visits.

### Sample size

#### Original sample size

The initial estimate of the required sample size was 2212 children (1106 per arm) across both countries pooled. This estimate was designed to detect a 30% reduction in the incidence rate of the composite primary outcome (death or all-cause readmission) from 469 per 1000 child years in the control arm to 328 per 1000 child years in the intervention arm (power 90%, α = 0.05), which allowed for one interim analysis and 15.7% loss to follow-up. For these estimations, we assumed an average pooled event rate of 399 per 1000 child years across the two arms, with 328 and 469 events per 1000 child years in the intervention and control arms respectively (Rate Ratio (RR) = 0.70). We based this assumption on observations in western Kenya (Desai et al., unpublished) and Malawi [17]. However, the observed event rate, pooled across both arms, during the first year of the study was 1120/1000 child years, which is almost three times higher than the assumed event rates. The higher rate is consistent with the recently published observations in Uganda [11]. Furthermore, the observed rate of loss to follow-up in the first 533 participants recruited and followed up for 6 months was 7% rather than the assumed 15%.

#### Sample size re-estimation

Following recommendations from the Data Monitoring and Ethics Committee (DMEC) and the Trial Steering Committee (TSC), a blinded interim sample size re-estimation was conducted to take into account the lower than expected rate of loss to follow-up and the higher than expected pooled incidence rate of the composite primary endpoint (death or all-cause readmission). This was favoured over an interim analysis, because the available funding did not allow an extension of the recruitment period, even if the results of any interim analysis had suggested that this would be required.

The revised sample size calculations show that a total sample size of 1040 children (520 per arm) is required to detect a 25% reduction in the incidence of the composite primary outcome from 1152 per 1000 child years (530 events per 1000 children) in the control arm to 864 per 1000 child years (398 per 1000 children) in the intervention arm (power 80%, α = 0.05), allowing for 10% loss to follow-up. The same sample size also provides 90% power to detect a 28.7% reduction in the primary endpoint from 1152 to 822 events per 1000 child years.

### Assignment of interventions

#### Allocation

Eligible children are randomly assigned (1:1) to either PMC-DP or placebo by a computer-generated randomisation schedule stratified by weight (per DP dosing schedule) and study site using permuted blocks of random sizes (see Additional file 1: page 30, Table S4) [12]. Recruitment is ‘competitive’ between the sites in the trial.

#### Blinding

The study is double-blinded to both participants/caretakers and study staff. Allocation concealment is achieved by the use of sealed opaque envelopes, with each envelope containing three other small envelopes (one for each PMC course). The envelopes containing active DP or placebo look identical, and the appearance and consistency of the tablets are also identical.

### Laboratory procedures

Hb is measured using HemoCue 201 (HemoCue, Angelholm, Sweden) photometers. Thick and thin blood films for parasite counts are obtained and examined. The films are read by two independent microscopists by counting any malaria parasites against 200 high-power fields before a slide is declared negative [45]. Point-of-care malaria diagnosis will be conducted using the First Response® Malaria Ag. pLDH/HRP2 Combo Card Test (Premier Medical Corporation, Mumbai, India).

### Statistical methods

A detailed study statistical analytical plan for the final analysis that will supersede the study protocol will be developed during the study before the unblinding of data.

### Analysis populations

The intention-to-treat (ITT) population is defined as all randomised subjects allocated to one of the two treatment arms and will be analysed in the group to which they were randomised, regardless of the type (placebo or active PMC) or number of courses received. The per-protocol (PP) population is a subset of the ITT population, excluding participants with major protocol deviations.

### Missing data

Every effort is being made to minimise the amount of missing data in the trial, and whenever possible, information on the reason for missing data is obtained. No adjustments will be made for missing outcome data, but missing data may be imputed for covariates.

### Assessment of efficacy

Primary analysis will be by ITT and will include all primary endpoint events (i.e. first and repeat events). The follow-up time will be measured as the time in days from the date of randomisation to the end of follow-up (around 26 weeks), death or drop-out. The incidence rate will be calculated per arm and the incidence rate ratio (IRR, PMC to placebo) and 95% confidence interval (CI)estimated using Poisson regression models with treatment (as randomised) as the only covariate. The results will also be expressed as the relative rate reduction (RRR) (95% CI).

#### Sub-group analysis

We will use stratified analysis to assess to what extent the effect of the intervention on the primary outcome is influenced by country, demographic parameters (e.g. age, ethnicity and socio-economic status), clinical parameters, malaria transmission variables (malaria transmission intensity, residence (urban/rural), season, insecticide-treated nets use, site), time of assessment and potential intervention modifiers. Because we did not power the study for sub-group analyses, we will interpret the results of the sub-group analysis cautiously. No adjustment will be made for multiple comparisons.

#### Sensitivity analyses

A number of sensitivity analyses will be conducted to assess the robustness of the primary endpoint analysis. These include analysis of the PP subject population and a covariate adjusted analysis. Other regression models will also be explored. Additional post hoc analyses may also be conducted if deemed appropriate. In addition, we will compare the results of the covariate-adjusted analyses with and without imputation for missing values for covariate values at baseline.

### Analysis of adverse events

Adverse events and serious adverse events are monitored, managed and recorded during the study. They will be recorded and tabulated for each treatment arm, overall, and per body system. Treatment emergent adverse events are defined as adverse events that had an onset day on or after the day of the first dose of study medication. No formal statistical testing will be undertaken. Enrolled children who are clinically unstable 2 weeks post-discharge (i.e. at the time eligibility is assessed for randomisation) and/or have rebound severe anaemia are re-admitted and become eligible for randomisation if they fulfil the entry criteria 2 weeks after the subsequent discharge.

### Procedures for assessing efficacy and safety parameters

#### Primary efficacy outcome

##### All-cause mortality

All-cause mortality will be assessed during visits 4 (2 weeks), 5 (6 weeks) and 6 (10 weeks) and at 18 weeks (by phone) and during the end-of-study assessment at 26 weeks.

##### All-cause and disease-specific readmissions

These readmissions will be assessed through passive case detection as well as a questionnaire administered during visits 4–7 at 2, 6, 10 and 26 weeks and during unscheduled sick visits. Details of admissions and treatment that the participants received are recorded including malaria diagnostic test results and use of antimalarials to allow for differentiation between malaria, severe anaemia and other syndromes.

#### Secondary efficacy outcomes

Secondary efficacy outcomes include all-cause and malaria-specific clinic visits. They will be assessed through passive case detection as well as questionnaires administered during visits 4–7 at 2, 6, 10 and 26 weeks and during unscheduled sick visits. Details of clinic visits are recorded including malaria diagnosis results to allow for differentiation between malaria and non-malaria clinic visits.

### Adverse events

We will adhere to the International Conference on Harmonisation (ICH) good clinical practice (GCP) principles in recording, reporting and managing adverse events and serious adverse events for all participants in both arms (see Additional file 1: page 51, section 9.6.2).

### Cardiac monitoring sub-study

The main safety concern with DP is its dose-dependent QTc prolongation induced by the piperaquine component. Transient QTc prolongation has been confirmed in clinical trials, but there are no data suggesting that the treatment is associated with clinically significant arrhythmias [38, 46, 47]. A trial in Uganda among children 6–24 months old included monthly DP for up to 18 monthly courses. A detailed sub-study of the effect of DP on cardiac repolarisation was conducted in 26 children and concluded that DP is not associated with a trend toward increasing QTc prolongation with increasing number of DP courses [38]. This type of safety data is limited, and we will therefore conduct a nested cardiac monitoring sub-study at Jinja Regional Referral Hospital in Uganda among 66 children who will be selected through convenience sampling. Separate written informed consent will be sought for inclusion in this sub-study. Approximately half of these children are expected to have received PMC with DP. The primary objective is to determine whether transient QTc prolongation increases in magnitude with subsequent courses of DP. Children enrolled in the sub-study will have an ECG taken prior to the first dose of each course and again 4–6 h after taking the third dose of each course of DP (anticipated maximum drug concentration).

## Discussion

Severe anaemia and severe malaria constitute a major public health problem in malaria endemic areas of Africa. Evidence suggests that a major, potentially preventable, component of the burden occurs after discharge and that a proactive approach is needed. Currently, no strategy specifically addresses this high-risk post-discharge period. This study seeks to determine the efficacy, safety and cost-effectiveness of 3 months of malaria chemoprevention post-discharge as an innovative strategy to reduce all-cause readmissions and deaths among children admitted with severe anaemia in malaria endemic areas. The study settings in Kenya and Uganda are representative of the main epidemiological settings appropriate for this intervention. Members from our consortium, under the leadership of the College of Medicine in Malawi, are concurrently conducting a trial in Malawi, under a separate protocol, on potential delivery mechanisms and health services research to determine the uptake, effectiveness, acceptability and feasibility of different mechanisms for delivering PMC (ClinicalTrials.gov: NCT02721420). This strategy builds on existing approaches used for seasonal malaria chemoprevention in west Africa and experience with IPT in pregnant women and infants [48, 49]. Should PMC prove to be effective, cost-effective and feasible, it may be a promising strategy to reduce all-cause readmissions and deaths in children admitted with severe anaemia in malaria endemic areas of Africa.

### Trial status

Recruitment started in May 2016 and is ongoing. Unblinding and analysis will begin after recruitment and follow-up are completed and the database has been completed, cleaned and locked.

## Additional files

Additional file 1: Full study protocol (including SPIRIT figure): v4.0, dated 06 Feb 2018. (PDF 3510 kb)

Additional file 2: Ethics approvals: KEMRI, SOMREC, LSTM, REK vest and CDC. (ZIP 1940 kb)

## Abbreviations

ACT: Artemisinin-based combination therapy
AL: Artemether-lumefantrine
CDC: Centers for Disease Control and Prevention
CGHR: Centre for Global Health Research
CI: Confidence interval
DBS: Dry blood spot
DP: Dihydroartemisinin-piperaquine
DSMB: Data Safety and Monitoring Board
ECG: Electrocardiogram
EDCTP2: European & Developing Countries Clinical Trials Partnership
GCP: Good clinical practice
GLOBVAC: Global Health and Vaccination Research
GMP: Good manufacturing practices
Hb: Haemoglobin
ICH: International Conference on Harmonisation
IPT: Intermittent preventive therapy
IPTi: Intermittent preventive therapy in infants
IPTp: Intermittent preventive therapy in pregnancy
IPTpd: Intermittent preventive therapy post-discharge
ITN: Insecticide-treated net
ITT: Intention-to-treat
KEMRI: Kenya Medical Research Institute
LSTM: Liverpool School of Tropical Medicine
PMC: Post-discharge malaria chemoprevention
PP: Per-protocol
RDT: Rapid diagnostic test
RRR: Relative rate reduction
SA: Severe anaemia
SMA: Severe malarial anaemia
SMC: Seasonal malaria chemoprevention
SOMREC: School of Medicine Research and Ethics Committee
SP: Sulphadoxine pyrimethamine
SSA: Sub-Saharan Africa
TSC: Trial Steering Committee
WHO: World Health Organization
