# Supplementary material for: Malaria chemoprevention with monthly dihydroartemisinin-piperaquine for the post-discharge management of severe anaemia in children aged less than 5 years in Uganda and Kenya: study protocol for a multi-centre, two-arm, randomised, placebo-controlled, superiority trial
Source: Trials. 2018 Nov 6;19:610. doi: 10.1186/s13063-018-2972-1 (PMC6220494; doi:10.1186/s13063-018-2972-1)
Supplement: Supplementary file 1 — Full study protocol (including SPIRIT figure): v4.0, dated 06 Feb 2018. (PDF 3510 kb) [file 13063_2018_2972_MOESM1_ESM.pdf]

Malaria Chemoprevention with monthly treatment with dihydroartemisinin-piperaquine for the post-discharge management of severe anaemia in children aged less than 5 years in Uganda and Kenya: A 3-year, multi-centre, parallel-group, two-arm randomised placebo controlled superiority trial

Short Title: Post-discharge Malaria Chemoprevention (PMC) study

Study Identifiers:

KEMRI:	LSTM REC:	Norway REC:	Uganda REC:	Primary Registry
#2965	#14.034	#2014/1911	#2015-125	Clinicaltrials.gov NCT02671175

Chief Investigator:

- Prof Feiko ter Kuile, KEMRI/CDC and LSTM, Pembroke Place, L3 5QA Liverpool, United Kingdom: +44 151 705 3287; and P.O. Box 1578, Kisumu 40100, Mobile: +254 708 739 228; E-mail: feiko.terkuile@lstmed.ac.uk

Country Co-Principal Investigators:

Uganda

- Dr Richard Idro, Department of Paediatrics and Child Health, College of Health Sciences, Makerere University, P.O Box 7072, Kampala Uganda; Mobile +256 774274173, E-mail: ridro1@gmail.com
- Dr Robert Opoka, College of Health Sciences, Makerere University, P.O Box 7072, Kampala Uganda, Mobile + 256 772996164, Email: opokabob@yahoo.com

Kenya

- Dr Simon Kariuki, KEMRI Centre for Global Health Research (CGHR) and CDC Collaboration, P.O. Box 1578, Kisumu 40100. Mobile: + 254 725 389 246; E-mail: SKariuki@kemricdc.org
- Dr Titus Kwambai, Kenya Ministry of Health, POBox 486 KISUMU 40100, and KEMRI Centre for Global Health Research (CGHR), P.O. Box 1578, Kisumu 40100. Mobile: +254 723354238; E-mail: tkkwambai@gmail.com

Co-Investigators: See Page 8

Funder: The Research Council of Norway, Global Health and Vaccination Research (GLOBVAC)

Sponsor: Liverpool School of Tropical Medicine (LSTM); Pembroke Place, Liverpool L3 5QA, UK
Phone: +44 0151 7053794; Email: lstmgov@lstmed.ac.uk

Revision chronology:

Date	Protocol Version	Details of Changes	Authors (see page 8)	Signature Chief Investigator
25 Aug 2015	1.2 Original		FtK, RI, CJ, RO, MD, SK, MO, BF, MH, JG, JB, MBvH, BR	[signature]
12 Nov 2015	2 Amendment	• Changed brand name for dihydroartemisinin-piperaquine (DP) from Duocotecxin to Eurartesim<br>• Page 30, Table 4; Updated the dose schedule to WHO's new dose recommendations for DP.<br>• Page 123, Added package insert for Eurartesim		[signature]
28 Oct 2016	3.0 Amendment	• Page 10 and 10; Updated the trial registration data.<br>• Page 15, Table 2; Updated the range in days for the scheduled follow-up visits<br>• Page 23; Expanded the number of study sites<br>• Pages 11, 13, 26: Changed minimum weight from >5kg to ≥5 kg<br>• Page 28: added malaria smears to RDT as option to diagnose clinical malaria for unscheduled visits<br>• Pages 34, 74 and 77: Introduced phone call reminder at 18 weeks post enrolment in protocol, patient information sheet and consent statement.<br>• Pages 77, 81, and 85: Minor editorial changes to consent statement for main trial, long term storage of blood and ECG sub study by adding a space to fill the patient ID number and a header to show where to place any thumbprint.		[signature]
06 Feb 2018	4.0 Amendment	• Pages 10, 14, 21 and 40: Revision of sample size		[signature]

Confidentiality Statement: This document contains confidential information that must not be disclosed to anyone other than the sponsor, the investigator team, host institution, relevant ethics committee and regulatory authorities

TABLE OF CONTENTS

1. Title of research protocol ... 8
2. Investigators and institutions ... 8
2.1. Investigators ... 8
2.1.1. Chief Investigator ... 8
2.1.2. Country co-Principal Investigators ... 8
2.1.3. Co-investigators ... 8
2.2. Non-Engaged Collaborators ... 9
2.3. Institutions ... 9
3. Protocol summaries ... 10
3.1. Trial Registration data ... 10
3.2. Narrative Protocol summary ... 13
4. Introduction ... 18
4.1. Severe anaemia and post-discharge mortality ... 18
4.2. Malaria as cause of post-discharge severe anaemia and mortality ... 18
4.3. Prevention of malaria post-discharge and haematological recovery ... 18
4.4. IPT-pd trial Malawi 2013 ... 19
4.5. Proposed Confirmatory trial in Kenya and uganda ... 19
5. Justification for the study ... 19
5.1. Why is this study needed now? ... 19
5.2. Rationale for another placebo controlled trial ... 20
6. Hypothesis ... 20
7. Aim and objectives ... 21
7.1. Aim ... 21
7.2. Objectives ... 21
7.2.1. Primary objective ... 21
7.2.2. Secondary objectives ... 21
8. Design and methodology ... 21
8.1. Overview study design ... 21
8.2. Design Considerations ... 21
8.2.1. Rationale for choice of DP for PMC ... 21
8.2.2. Why in this study population? ... 22
8.2.3. Efficacy vs effectiveness ... 22
8.2.4. Why this composite primary outcome? ... 22
8.2.5. Rationale for assessment by 6 months after enrolment ... 22
8.2.6. Rationale for testing of malaria and anaemia associated genes ... 23
8.2.7. Rationale for testing parasite genetics and resistance-associate genes ... 23
8.3. Study settings ... 23
8.3.1. Kenya ... 24
8.3.2. Uganda ... 25
8.4. Eligibility criteria ... 26
8.4.1. Eligibility criteria for Pre-Study Screening ... 26
8.4.2. Eligibility criteria for enrolment into study ... 26
8.4.3. Eligibility criteria for randomisation into study (at 2 weeks) ... 27
8.5. Interventions ... 28

8.5.1. Standard in-hospital and post-discharge care (not study specific or study related)....28
8.5.2. Trial Medication and Interventions....29
8.5.3. Procedures for Drug handling & Accountability....31
8.5.4. Removal of Patients from Therapy or Assessment....32
8.5.5. Discontinuation from storage of blood for future studies....33
8.5.6. Adherence to study intervention protocol and strategies for retention....34
8.5.7. Prior and concomitant therapy....34
8.6. Outcomes....35
8.6.1. Primary efficacy outcome....35
8.6.2. Key secondary efficacy outcome....35
8.6.3. Other secondary efficacy outcomes....36
8.6.4. Tolerability and safety....36
8.6.5. Economic evaluation outcomes....36
8.7. Participants timeline....37
8.7.1. Overview Study Phases....37
8.7.2. Visit 1: Pre-screening....37
8.7.3. Visit 2: Screening interview and consent & Enrolment....38
8.7.4. Visit 3: AL treatment in hospital/discharge....39
8.7.5. Visit 4: Randomisation and 1st PMC treatment visit....39
8.7.6. Visits 5 and 6: 2nd and 3rd PMC treatment visits....39
8.7.7. Visit 7: 6-month scheduled follow-up....40
8.7.8. Unscheduled visits (passive follow-up)....40
8.8. Sample size....40
8.8.1. Trial....40
8.8.2. Cardiac monitoring study....41
8.9. Recruitment strategies for achieving target sample size....41
8.10. Assignment of interventions and blinding....42
8.10.1. Allocation....42
8.10.2. Blinding....43
9. Data collection, management and analysis....44
9.1. Data collection methods....44
9.1.1. Data management....44
9.2. Statistical methods....44
9.2.1. Trial profile and flowchart....44
9.2.2. Baseline characteristics....44
9.2.3. Analysis Populations....45
9.2.4. Missing Data....45
9.2.5. Assessment of efficacy....45
9.2.6. Analysis of adverse events....47
9.3. Procedures for Assessing Efficacy Parameters....47
9.3.1. Primary outcome....47
9.3.2. Secondary outcomes....48
9.4. Laboratory methods....48
9.4.1. Techniques for host and parasite genetic assays....48
9.5. Monitoring....48
9.5.1. Data Monitoring....48

9.6. Safety monitoring and reporting 49
9.6.1. Cardiac Monitoring 49
9.6.2. Definitions 51
9.6.3. Reporting adverse event procedures 51
9.7. Quality assurance 54
9.7.1. Clinical monitoring and auditing 54
9.7.2. Training 54
9.7.3. Quality assurance/control of laboratory tests 55
9.8. Economic Evaluation sub-Study 55
9.8.1. Patient intervention costs 56
9.8.2. Patient disease costs 56
9.8.3. Provider intervention costs 56
9.8.4. Provider disease costs 56
10. Timeframe and duration of the study 56
11. Ethical considerations & regulatory approvals 57
11.1. Declaration of Helsinki 57
11.2. Research Ethics Approval 57
11.2.1. Review process 57
11.2.2. Protocol amendments 57
11.3. Regulatory Approval and Trial Authorisation 57
11.4. Informed Consent procedures 57
11.4.1. Consent forms 58
11.5. Protection of Privacy and confidentiality 58
11.5.1. Privacy 58
11.5.2. Privacy of individual 58
11.5.3. Confidentiality of data 58
11.6. Declaration of interest 59
11.7. Access to Source Data/Documents 59
11.8. Risks and benefits 59
11.8.1. Risks 59
11.8.2. Benefits 60
11.9. Ancillary and post-trial care 61
11.9.1. Health care during the trial 61
11.9.2. Trial insurance 61
11.9.3. Post-trial care 61
11.10. Expenses reimbursement and incentives 61
12. Dissemination and application of the results 62
12.1.1. Result dissemination and publication policy 62
12.1.2. Impact 63
12.1.3. Training, Fellowships and Capacity Building 63
12.1.4. Authorship and publications 63
12.1.5. Data Sharing Statement 64
13. References 64
14. Financial aspects and conflict of interest 69
14.1.1. Funding of the trial 69
14.1.2. Provision of the study drugs 69
15. Budget and budget Justification 69

16. Appendices 70
16.1. Appendix I. Role Investigators and non-engaged collaborators 70
16.1.1. Protocol development: authors' contributions 70
16.1.2. Role Investigators 70
16.1.3. Role Non-Engaged Collaborators 71
16.2. Appendix II. Participant information sheets and informed consent forms 72
16.2.1. Participant Information Sheet for main trial (English) 72
16.2.2. Consent statement for main trial (English) 77
16.2.3. Participant Information Sheet long-term storage/ future studies (English) 78
16.2.4. Consent statement for long-term storage / future studies (English) 81
16.2.5. Participant Information Sheet for ECG sub study (English) 82
16.2.6. Consent statement for ECG sub study (English) 85
16.2.7. Participant Information Sheet for main trial (DhoLuo) 86
16.2.8. Consent statement for main trial (DhoLuo) 91
16.2.9. Participant Information Sheet long-term storage / future studies (Dholuo) 92
16.2.10. Consent statement long-term storage / future studies (Dholuo) 95
16.2.11. Participant Information Sheet for ECG sub study (Dholuo) 96
16.2.12. Consent statement for ECG sub study (Dholuo) 98
16.2.13. Participant Information Sheet for main trial (Kiswahili) 99
16.2.14. Consent statement for main trial (Kiswahili) 104
16.2.15. Participant Information Sheet long-term storage / future studies (Kiswahili) 105
16.2.16. Consent statement for long-term storage / future studies (Kiswahili) 108
16.2.17. Participant Information Sheet for ECG sub study (Kiswahili) 109
16.2.18. Consent statement for ECG sub study (Kiswahili) 112
16.3. Appendix III. Terms of Reference Oversight committees 113
16.3.1. Trial Management Group (TMG) 113
16.3.2. Trial Steering Committee (TSC) 113
16.3.3. Data Monitoring and Ethics Committee (DMEC) 115
16.4. Appendix IV.Declaration of Helsinki 116
16.5. Appendix V. Budget 119
16.6. Appendix VI. Budget Justification 119
16.7. Appendix VII. SPIRIT 2013 Checklist clinical trial protocol 120
16.8. Appendix VIII. Eurartesim package insert 125
16.9. Appendix IX. Questionnaires 132

Abbreviations

ACT	Artemisinin based combination therapy
AE	Adverse event
AIDS	Acquired immunodeficiency syndrome
CDC	Centers for Disease Control and Prevention
CRF	Case Report form
CRO	Contract Research Organisation
ECG	Electrocardiogram
ERG	Evidence Review Group World Health Organization
DHFR	Dihydrofolate reductase
DP	Dihydroartemisinin-piperaquine
DHPS	Dehydropteroate synthetase
DMEC	Data Monitoring and Ethics Committee
G6PD	Glucose-6-phosphate dehydrogenase
GCP	Good Clinical Practice
Globvac	Global Health and Vaccination Research, Research Council of Norway
IB	Investigator's Brochure
IMP	Investigational Medicinal Product
LSTM	Liverpool School of Tropical Medicine
HIV	Human Immuno-deficiency Virus
IEC/IRB	Independent Ethics Committee / Institutional Review Board
ITT	Intention to Treat
ICH	The International Conference on Harmonisation
IPTpd	Intermittent Preventive Therapy post-discharge
NSD	Norwegian Social Science Data Services, Research Council of Norway
PCR	Polymerase Chain Reaction
PMC	Post-discharge Malaria Chemo-prevention
PK	Pharmacokinetic
SD	standard deviation
SMC	Seasonal Malaria Chemo-prevention
SOP	standard operating procedure
SP	Sulfadoxine-pyrimethamine
SSA	Sub-Saharan Africa
T1/2	plasma half-life
Tmax	time to maximum plasma concentration
URTI	upper respiratory tract infection
QRS	Time interval between Q-,R- and S- waves on ECG records
QT	Time interval between Q- to T-wave (interval of electrocardiogram)
QTc	QT corrected
RBC	Red Blood Cells
SAE	Serious Adverse Event
WHO	World Health Organisation

1. TITLE OF RESEARCH PROTOCOL

Malaria Chemoprevention with monthly treatment with dihydroartemisinin-piperaquine for the post-discharge management of severe anaemia in children aged less than 5 years in Uganda and Kenya: A 3-year, multi-centre, parallel-group, two-arm randomised placebo controlled superiority trial

Short Title: Post-discharge Malaria Chemoprevention (PMC) study

2. INVESTIGATORS AND INSTITUTIONS

2.1. INVESTIGATORS

2.1.1. Chief Investigator

Prof Feiko ter Kuile, KEMRI/CDC and LSTM, Pembroke Place, L3 5QA Liverpool, United Kingdom: +44 151 705 3287; and P.O. Box 1578, Kisumu 40100, Mobile: +254 708 739 228; E-mail: feiko.terkuile@lstmed.ac.uk

2.1.2. Country co-Principal Investigators

Uganda

- Dr Richard Idro, Department of Paediatrics and Child Health, College of Health Sciences, Makerere University, P.O Box 7072, Kampala Uganda; Mobile +256 774274173, E-mail: ridro1@gmail.com
- Dr Robert Opoka, College of Health Sciences, Makerere University, P.O Box 7072, Kampala Uganda, Mobile + 256 772996164, Email: opokabob@yahoo.com

Kenya

- Dr Simon Kariuki, KEMRI Centre for Global Health Research (CGHR) and CDC Collaboration, P.O. Box 1578, Kisumu 40100. Mobile: + 254 725 389 246; E-mail: SKariuki@kemricdc.org
- Dr Titus Kwambai, Kenya Ministry of Health, POBox 486 KISUMU 40100, and KEMRI Centre for Global Health Research (CGHR), P.O. Box 1578, Kisumu 40100. Mobile: +254 723354238; E-mail: tkkwambai@gmail.com

2.1.3. Co-investigators

Uganda	Dr. Aggrey Dhabangi[3]	*USA /Kenya*	Dr Aaron Samuels[9]
	Dr Tom Didimus Ediamu[4]	*Malawi*	Prof Kamija Phiri[12]
	Dr Sophie Namasopo[5]	*Norway*	Prof Bjarne Robberstad[13]
	Dr Harriet Nambuya[5]	*UK*	Prof Brian Faraghar[1]
Kenya	Dr Martina Oneko[2]		Prof Duolao Wang[1]
	Dr Grace M Nalwa[7]	*USA*	Prof Chandy John[14]
	Dr Magdalene Kuria[8]		Prof Jonathan J. Juliano[15]
			Prof Jeffrey A. Bailey[16]

2.2. NON-ENGAGED COLLABORATORS

- Dr. Thomus Ochar[6]
- Dr Andrew Nyandigisi[10]
- Dr Omondi Owino[11]
- Prof Azra Ghani[17]
- Dr Matthew Cairns[17]
- Dr Mary Hamel[9]
- Dr Julie Gutman[9]
- Dr Meghna Desai[9]
- Prof M. Boele van Hensbroek[18]

2.3. INSTITUTIONS

1. Liverpool School of Tropical Medicine (LSTM), Liverpool, United Kingdom
2. KEMRI Centre for Global Health Research (CGHR), Kisian, Kenya
3. College of Health Sciences, Makerere University, Kampala Uganda
4. Hoima Regional Referral Hospital, Hoima, Uganda
5. Jinja Regional referral hospital, Uganda
6. Tororo Hospital, Tororo, Uganda
7. Migori County Referral Hospital, Migori, Kenya
8. Kisumu County Referral Hospital, Kisumu, Kenya
9. Division of Parasitic Diseases and Malaria, US Center for Disease Control and Prevention (CDC), Atlanta, GA, USA
10. National Malaria Control Program, Ministry of Health Kenya, Nairobi, Kenya
11. Ministry of Health, Siaya County, Siaya, Kenya
12. College of Medicine, University of Malawi, Blantyre, Malawi
13. Centre for International Health, &Department of Global Public Health and Primary Care, University of Bergen, Bergen, Norway
14. Ryan White Center for Pediatric Infectious Disease and Global Health, Indiana University School of Medicine, Indianapolis, IN, USA
15. Division of Infectious Diseases, School of Medicine, University of North Carolina, Chapel Hill, USA
16. Department of Medicine Division of Transfusion Medicine and Program in Bioinformatics and Integrative Biology, University of Massachusetts School of Medicine, Worcester, USA
17. MRC Centre for Outbreak Analysis & Modelling, Department of Infectious Disease Epidemiology, Imperial College London, London, United Kingdom
18. Emma Children's Hospital, Academic Medical Centre, University of Amsterdam, the Netherlands

3. PROTOCOL SUMMARIES

3.1. TRIAL REGISTRATION DATA

Data Category	*Information*			
Primary registry and trial identifying number	ClinicalTrials.gov NCT02671175			
Date of registration in primary registry	28/01/2016			
Secondary identifying numbers	KEMRI: #2965	LSTM: #14.034	Norway REC: #2014/1911	Uganda REC:#2015-125
Source(s) of monetary or material support	The Research Council of Norway, Global Health and Vaccination Research (GLOBVAC), grant 234487			
Primary sponsor	Liverpool School of Tropical Medicine (LSTM)			
Secondary sponsor(s)	NA			
Contact for public queries	Prof Feiko ter Kuile, E-mail: terkuile@liv.ac.uk			
Contact for scientific queries	Prof Feiko ter Kuile, LSTM, Pembroke Place, Liverpool, L3 5QA, UK, E-mail: terkuile@liv.ac.uk			
Public title	Malaria chemoprevention in the post-discharge management of children with severe anaemia in Kenya and Uganda			
Scientific title	Malaria Chemoprevention with monthly treatment with dihydroartemisinin-piperaquine for the post-discharge management of severe anaemia in children aged less than 5 years in Uganda and Kenya: A 3-year, multi-centre, parallel-group, two-arm randomised **placebo controlled superiority trial**			
Countries of recruitment	Kenya and Uganda			
Health condition(s) or problem(s) studied	Malaria, readmissions after severe anaemia			
Intervention(s)	Dihydroartemisinin-piperaquine (3-day treatment courses, given 2, 6, and 10 weeks after enrolment)			
	Placebo comparator (matching tablets containing no active ingredients)			
Study type	Interventional			
	Allocation: randomised; intervention model: parallel assignment; arms:2; allocation ratio: 1:1; Masking: double blind placebo controlled			
	Primary purpose: prevention			
	Phase-III			
Date of first enrolment	06/05/2016			
Target sample size	Revised: 1040, original: 2212			
Recruitment status	Recruiting			

Category	*Information*
Key inclusion criteria	Pre-study screening<br>1. Haemoglobin <5.0 g/dl or PCV < 15%, or requirement for blood transfusion for other clinical reasons on or during admission to the hospital<br>2. Aged less than 59.5 months<br>3. Body weight ≥5 kg<br>4. Resident in catchment area<br>Enrolment in study(t=0)<br>1. Fulfilled the pre-study screening eligibility criteria<br>2. Aged < 59.5 months<br>3. Clinically stable, able to take oral medication<br>4. Subject completed blood transfusion(s) or became clinically stable without transfusion<br>5. Able to feed (for breastfeeding children) or eat (for older children)<br>6. Absence of know cardiac problems<br>7. Provision of informed consent by parent or guardian<br>Randomisation (t=2 weeks)<br>1. Fulfilled enrolment eligibility criteria and was enrolled during recent admission<br>2. Aged <60 months<br>3. Still clinically stable, able to take to oral medication, able to feed (for breastfeeding children) or eat (for older children) and able to sit unaided (for older children who were already able to do so prior to hospitalisation)
Exclusion criteria	Pre-study screening<br>1. Recognised specific other cause of severe anaemia (e.g. trauma, haematological malignancy, known bleeding disorder)<br>2. Known sickle cell disease<br>3. Anticipated to reach the 5th birthday (60 months of age) within 2 weeks from enrolment (i.e. prior to randomization)<br>4. Child will reside for more than 25%of the 6 months study period (i.e. 6 weeks or more) outside of catchment area<br>Enrolment in study (t=0)<br>1. Previous enrolment in the present study<br>2. Known hypersensitivity to study drug<br>3. Sickle cell disease<br>4. Use or known need at the time of enrolment for concomitant prohibited medication (see section 8.5.7.2, page 35) during the 14 weeks PMC treatment period (see section 8.5.7.2, page 35).<br>5. Ongoing or planned participation in another clinical trial involving ongoing or scheduled treatment with prohibited medicinal products or active follow-up during the course of the study (6 months from enrolment)

	6. A known need at the time of enrolment for scheduled surgery during the subsequent course of the study (6 months from enrolment)<br>7. Suspected non-compliance with the follow-up schedule<br>8. Know heart conditions, or family history of congenital prolongation of the QTc interval.<br>9. Taking medicinal products that are known to prolong the QTc interval<br>Randomisation (t=2 weeks)<br>1. Used DP since enrolment<br>2. Use or known need at the time of randomisation for concomitant prohibited medication (see section 8.5.7.2, page 35) during the 14 weeks PMC treatment period (see section 8.5.7.2, page 35).<br>3. Enrolled, or known agreement to enrol into another clinical trial involving ongoing or scheduled treatment with medicinal products during the course of the study (6 months from enrolment)<br>4. A known need at the time of randomisation for scheduled surgery during the subsequent course of the study (6 months from enrolment)<br>5. Suspected non-compliance with the follow-up schedule<br>1. Withdrawal of consent since enrolment
Category	*Information*
Primary outcome(s)	All-cause deaths or all-cause re-admissions by 26 weeks from randomization (composite primary outcome)
Key Secondary efficacy outcomes	1. Readmission due to severe malaria (defined as any treatment with parenteral quinine or artesunate, or presence of severe anaemia and treatment with oral antimalarials) by 26 weeks from randomization<br>2. Readmissions due to severe anaemia (defined as Hb <5g/dL or PCV <15% or requirement for blood transfusion based on other clinical indication) by 26 weeks from randomization<br>3. Readmission due to severe malarial anaemia (severe anaemia plus parenteral or oral antimalarial treatment) by 26 weeks from randomization<br>4. Readmission due to severe anaemia or severe malaria (composite outcome) by 26 weeks from randomization<br>5. All-cause mortality by 26 weeks from randomization<br>6. All-cause hospital readmission by 26 weeks from randomization<br>7. Clinic visits because of smear of RDT confirmed non-severe malaria
Safety outcomes	1. Serious adverse events, excluding primary and secondary efficacy outcomes, by 26 weeks from randomization<br>2. Serious adverse events within 7 days after the start of each course of PMC, excluding primary and secondary efficacy outcomes.<br>3. Adverse events by 26 weeks from randomization<br>4. Adverse events within 7 days after start of each course of PMC.<br>5. QTc prolongation measured by electro cardio gram (ECG)4-6 hours after 3rd dose of each course

3.2. NARRATIVE PROTOCOL SUMMARY

Title: Malaria Chemoprevention with monthly treatment with dihydroartemisinin-piperaquine for the post-discharge management of severe anaemia in children aged less than 5 years in Uganda and Kenya: A 3-year, multi-centre, parallel-group, two-arm randomised placebo controlled superiority trial.

Short Title: Post-discharge Malaria Chemoprevention (PMC) study

Background and rationale: Children hospitalised with severe anaemia in Africa are at high risk of readmission or death within 6 months after discharge. No strategy specifically addresses this post-discharge period. In Malawi, 3 months of post-discharge malaria chemoprevention (PMC) with monthly 3-day treatment courses of artemether-lumefantrine (AL) in children with severe malarial anaemia prevented 31% of deaths and readmissions. The effect was in addition to the effect of insecticide-treated bednets. These promising findings now need to be confirmed in other settings before the World Health Organisation can consider PMC for the post-discharge management of severe anaemia.

Primary efficacy objective: To determine if 3 months of post-discharge malaria chemoprevention with monthly 3-day treatment courses of dihydroartemisinin-piperaquine (DP) (PMC-DP) is safe and superior to the standard single 3-day treatment course with artemether-lumefantrine provided as part of standard in-hospital care in reducing all-cause readmissions and deaths by 6 months in the post-discharge management of children less than 5 years of age admitted with severe anaemia.

Hypothesis: An additional three months of malaria chemoprevention with monthly 3-day treatment courses with DHA-piperaquine (each providing about 4 weeks of post-treatment prophylaxis) provided during the post-discharge period to children recently admitted with severe anaemia is superior to reduce all-cause readmission and mortality rates by 6 months compared with 2 weeks of post-treatment prophylaxis provided by the single course of oral artemether-lumefantrine when given as part of the standard in-hospital care around the time of discharge.

Study Type: Multi-centre, 2-arm, placebo-controlled, individually randomized, trial of 3 courses of monthly PMC-DP in Uganda and Kenya, using randomisation stratified by age and study centre.

Sites: 7 hospitals, 4 in western Kenya and 3 in Uganda, in areas with moderate to intense malaria transmission. The number of hospitals will be expanded if recruitment if so required.

Study Population: Inclusion criteria: convalescent children aged less than 5 years and weighing ≥5 kg admitted with severe anaemia (haemoglobin<5g/dL / Ht<15%); clinically stable, able to take or switch to oral medication; post-transfusion Hb >5g/dL. Exclusion criteria: blood loss due to trauma, malignancy, known bleeding disorders or sickle cell disease, known hypersensitivity to study drug, known heart conditions, non-resident in study area, previous participation in study, known need at enrolment for prohibited medication and scheduled surgery during the 6-month course of the study. HIV infection and cotrimoxazole prophylaxis are not exclusion criteria.

Study Interventions: Children in both arms will receive standard in-hospital care for severe anaemia (blood transfusion, often combined with quinine or artesunate IV/IM). All children will then receive a 3-day course of AL (whether they initially had malaria or not), which will be started in-hospital as soon as they are able to take oral medication, and will be completed at home after discharge. At 2 weeks after enrolment surviving children will be randomized to receive either a standard 3-day

courses of DP (Eurartesim®, Sigma Tau, Italy) or an identical placebo regimen at 2, 6 and 10 weeks after enrolment.

Outcome Measures: Primary: Death or all-cause re-admission by 6 months from randomization (composite primary outcome). Key secondary: 1) all-cause mortality, 2) all-cause hospital readmission, 3) readmissions due to severe anaemia or severe malaria (requiring parenteral quinine or artesunate), 4) non-severe all-cause sick-child clinic visits, 5) clinic visit because of RDT or microscopy confirmed non-severe malaria.

Follow-up procedures: Children will be followed for 6 months by passive case detection in 3 phases: Pre-PMC (2 weeks between discharge and randomisation); PMC (2-14 weeks post-discharge); post-PMC (extended follow period from 15 to 26 weeks post-discharge).

Sample size: A sample size of 520 children per arm (1040 total children) allows detection of a 25% reduction in the incidence rate from 1,152 per 1000 child years (530 per 1000 children per 24 weeks) to 864 per 1000 child years, with 10% loss to follow-up (power 80%, α=0.05).

Data Analysis: Primary analysis will be by intention to treat. Incidence rates will be calculated and rate ratios estimated using Poisson regression, stratified by country, with treatment (as randomised) as the only co-variate. To assess how long any initial beneficial effect of PMC is sustained, the observation time will also be divided into a) PMC period (2-14 weeks); and b) Extended follow-up period (15-26 weeks post-discharge).

Partners institutions: University of Bergen; University of Malawi; Makerere University; KEMRI-CDC, Kenya; University of Indiana; Liverpool School of Tropical Medicine; London School of Hygiene & Tropical Medicine; Imperial College London; University of Amsterdam; US Centers for Disease Control and Prevention, University of North Carolina, and University of Massachusetts.

Table 1: Study treatment and phases

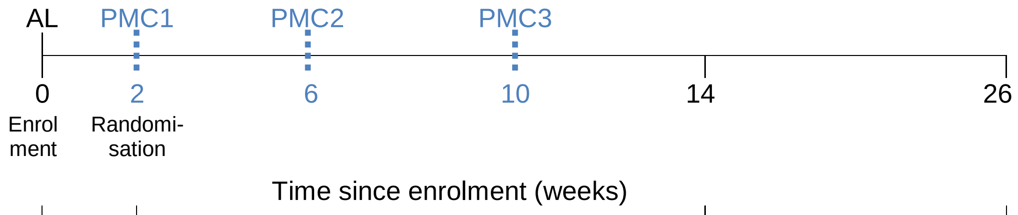

Period	Pre-PMC	PMC	Post-PMC(extended follow-up)
Trial intervention	AL near discharge, both arms	Three DP courses as PMC or placebo	No intervention
Rescue Rx acute malaria	Oral QN, 7 days	AL, 3 days	AL, 3 days
Treatment for severe malaria	Parenteral artesunate / quinine and oral AL, 3 days		Parenteral artesunate / quinine and AL

An end of study assessment will be done 26 weeks (6 months) after discharge.
AL=artemether-lumefantrine. PMC=Post-discharge Malaria Chemoprevention. DP=dihydroartemisinin-piperaquine. QN=quinine

Table 2: Study Design and Schedule of Assessment

Phase	Recruitment Phase	Enrolment	In-patient Hospitalisation phase			Randomisation	PMC Treatment Phase 12 weeks period from 2-14 weeks								Post-PMC Extended follow-up Phase
Location	In-Hospital					Clinic/ Home	Home								Clinic/home
Visit number	#1	#2		#3			#4			#5			#6		#7
Visit description	Pre-study Screening	Screening Consent & Base-line		AL treatment visit			t=2 weeks; Allocation & treatment visit			t=6 weeks treatment visit			T=10 weeks treatment visit		End of study Assessment
Study Time							2 weeks (day 14[11-28])[c]			6 weeks (day 42 [38-56])[c]			10 weeks (day 70 [66-84])[c]		
	Days -4[a]-0	Day0	Day0 Hosp	Day1 hosp/ home	Day2 hosp/ home	2w-Day1	2w-Day2	2w-Day3	6w-Day1	6w-Day2	6w-Day3	10w-Day1	10w-Day2	10w-Day3	6 Month (day 182 +/- 28)[c]
Recruitment															
Pre-screening eligibility	X														
Prior consent discussion	X														
Enrolment															
Eligibility screen		X													
Informed Consent		X													
Study code issued		X													
Allocation						X									
Interventions															
PMC-Placebo arm			AL1&2[b]	AL3&4[b]	AL5&6[b]	Plac1	Plac2	Plac3	Plac1	Plac2	Plac3	Plac1	Plac2	Plac3	
PMC-Active arm			AL1&2[b]	AL3&4[b]	AL5&6[b]	DP1	DP2	DP3	DP1	DP2	DP3	DP1	DP2	DP3	
Iron supplement.						Iron for 28 days from t=14-42 days									
Assessments															
**Baseline**															
Copy Clinic/Lab data from hospital records		X				X									
Physical Exam.		X				X									
Blood sample		2ml VP				FP-Hb/M									

						S[e]									
**Efficacy Outcomes**															
Physical exam/growth															X
Hb & Malaria & PCR															X
Clinic visits	Passive surveillance in clinics in the catchment area, 26 weeks from 0-26 weeks (clinical malaria and other acute illnesses) (RDT/smear, Hb, dried blood spots for parasite genetics)														
Hospitalisation	Passive surveillance for hospital admission in the catchment area, 26 weeks from 0-26 weeks														
Vital status			X	X	X	X	X	X	X	X	X	X	X	X	X
Pf genetics/resistance	X[f]	X				X									X
Host genetics		X													X
Patient costs					X										X
**Safety Outcomes**															
Adverse events			X	X	X	X	X	X	X	X	X	X	X	X	X
ECG[d]						X		X	X		X	X		X	

Visit #1: Pre-study Screening (around admission or shortly thereafter)

Visit #2: Screening Consent & Base-line (during convalescence)

Visit #3: Oral arthemether-lumefantrine (AL) consisting of 6 doses (2x daily for 3 days); first dose provided in hospital. Subsequent doses may be administered at home or in-hospital.

Visit #4: 2 weeks after enrolment. Participants will be randomised to one of the two treatment groups during this visit. They will also be given the first dose of PMC under observation. Doses of day 2 and 3 can be taken at home. All participants will get 1 month supply of iron during this visit.

Visit #5 #6: Home visits at 6 and 10 weeks after enrolment to issue participants with the 2nd and 3rd course of the PMC study drugs.

Visit #7: at 6 months after enrolment. This is the close out assessment.

a. Children can be pre-study screened any time between hospital admission and enrolment. The figure of -4 days is provided for illustration purposes only.

b. AL: Some children may have received AL as part of standard in-hospital care prior to enrolment (e.g. during days -1 or -2 and not as part of the study). They will have their number of study AL doses adjusted to ensure that no more than a cumulative total of 6 AL doses is provided. The day of enrolment is always considered as Day-0 regardless of when the first dose of AL was received.

c. Visit window= number of days an actual subject visit may fall outside of the planned protocol schedule visit to still meet protocol requirements. DP should be given at least 4 weeks apart.

d. ECG, Electro Cardio Gram, to be conducted in a sub-sample only. A capillary sample will be taken at the same time as the ECG for piperaquine drug levels.

e. MS, malaria smear. This will be collected for research purposes only, and read days to weeks later. Malaria smears will not be used for point of care. If participants are symptomatic (e.g. fever) an RDT will be taken for point of care.

f. Uses left over samples from blood-group typing and cross-matching or other clinical samples that were taken as part of routine care that would otherwise be discarded. Sample will only be used after consent has been obtained in the subsequent visit 2.

VP=vena puncture. FP=finger prick, Plac=Placebo DP, DP=dihydroartemisinin-piperaquine, AL=artemether-lumefantrine, Hb=haemoglobin, MS=malaria smear, Pf=Plasmodium falciparum

Figure 1: Flow of Participants

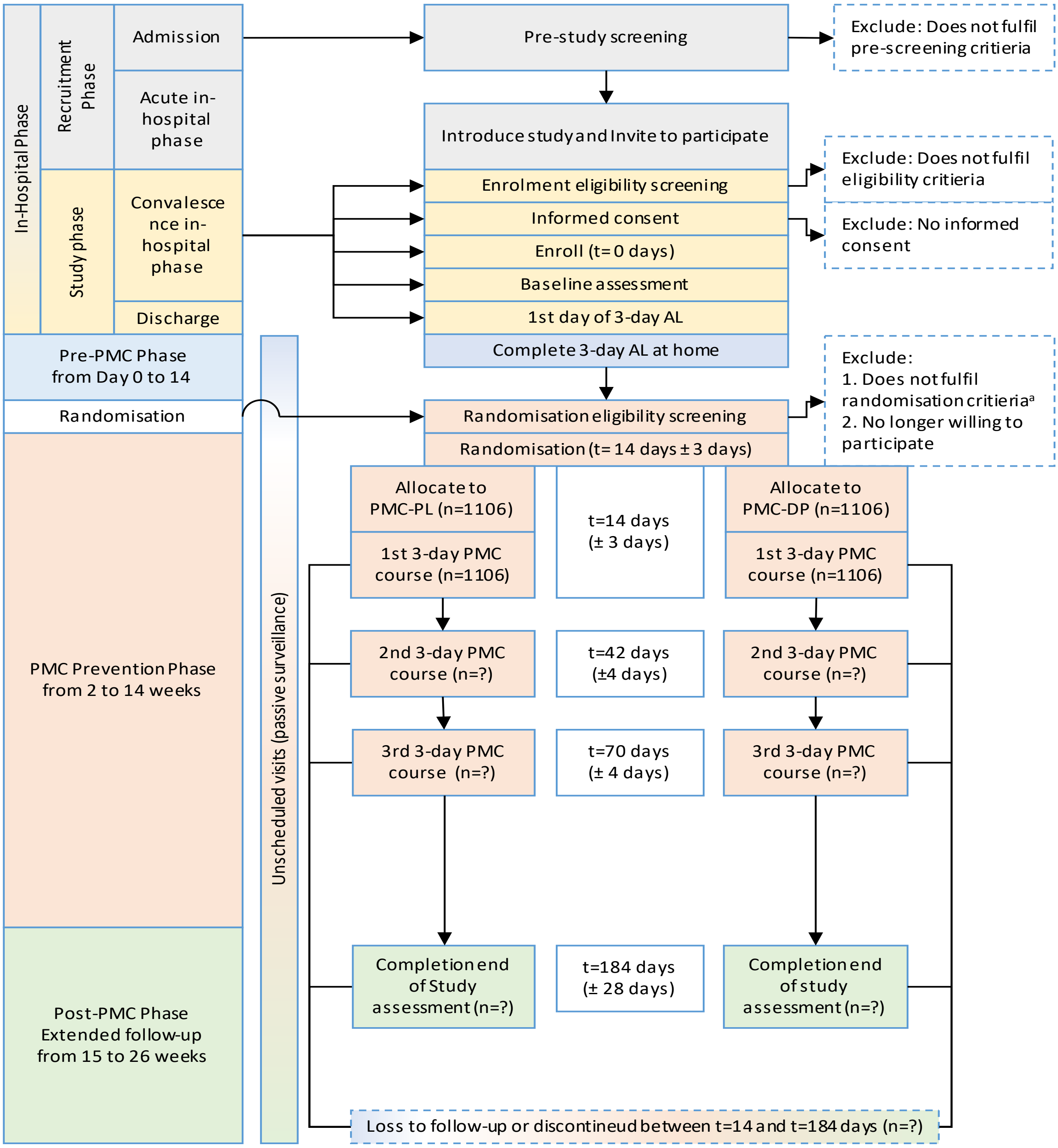

a. Children who fulfil the enrolment criteria but not the randomisation criteria will not be randomised, but where feasible will continued to be followed until the end of study at 26 weeks

4. INTRODUCTION

4.1. SEVERE ANAEMIA AND POST-DISCHARGE MORTALITY

Severe anaemia is a leading cause of hospital admissions in Africa contributing substantially to paediatric mortality. Our recent case-control study in Malawian children indicated that children aged <5y admitted with severe anaemia are not only at high risk of dying during the acute phase in-hospital (6%) but also for several months after they leave hospital: by 6 months post-discharge an additional 8% had died, which is nine times higher than the mortality rates in community-based, age matched children with mild anaemia.[1,2] A further 8% were readmitted with rebound severe anaemia.[2] Similar rates of post-discharge morbidity and mortality are seen in western Kenya, where 17.9% of children <5y admitted with severe anaemia died or were readmitted by 6 months (Desai et al, unpublished observations), while in Uganda as many as 36.5% died or were readmitted within 6 months (C. John et al, unpublished data). Hospitalised children with severe anaemia are particularly at risk within the first 3 months post-discharge, likely due to a combination of environmental, behavioural, nutritional and genetic risk factors.[2,3,4]

4.2. MALARIA AS CAUSE OF POST-DISCHARGE SEVERE ANAEMIA AND MORTALITY

Because most deaths occurred at home the cause of death could not be assessed. However, previous observational studies in western Kenya[5,6] and a recent intervention study in a high transmission area in Malawi showed that malaria in the post-discharge period is an important contributor responsible for a slow haematological recovery, rebound severe anaemia and morbidity.[7]Many children in these areas experience episodes of new or recrudescent malaria infections after discharge which negates the initial rise in haemoglobin (Hb) achieved by blood transfusion in hospital.[5] Haematological recovery from malaria-associated anaemia is known to take at least 6 weeks. This period may be prolonged in those with persistent or new malaria infections due to on-going red cell destruction and red blood cell production failure.[89]

4.3. PREVENTION OF MALARIA POST-DISCHARGE AND HAEMATOLOGICAL RECOVERY

Standard treatment for severe anaemia in many countries in sub-Saharan Africa (SSA) consists of a blood transfusion, antibiotics if bacterial infections are suspected, and in the case of severe malarial anaemia, parenteral anti-malarial treatment (quinine or artesunate). Once children have stabilised and can be switched to oral treatment this is completed with short courses of 3-day treatment with artemisinin-based combination therapy (ACT), usually artemether-lumefantrine (AL). Children are often discharged with a short course of iron and folate, typically with no scheduled follow-up.

We hypothesize that by creating a prophylactic-time-window post transfusion, the bone marrow gets time to recover, resulting in a more sustained haematological recovery post-discharge. Data from Malawi show that this process takes 2-3 months in children with severe anaemia. The use of Intermittent Preventive Therapy (IPT) in children with 2 or 3 courses of artesunate plus

sulphadoxine-pyrimethamine, spaced monthly during the rainy season reduced clinical attacks of malaria by 86% in areas with highly seasonal transmission.[10]IPT is the administration of a full treatment course of long-acting antimalarials at pre-defined time intervals irrespective of a patient's malaria status and clears existing infections and provides prolonged prophylaxis against new infections.[11] The World Health Organisation's (WHO) recommended malaria control strategies include IPT for pregnant women (IPTp)[12,13] and for infants (IPTi)[14] and children (IPTc)[15] living in areas with seasonal malaria transmission (now called seasonal malaria chemoprevention or SMC).[16]

4.4. IPT-PD TRIAL MALAWI 2013

We recently completed a multi-centre, randomized, placebo-controlled trial to determine the impact of malaria chemoprevention with Intermittent Preventive Therapy post-discharge (IPTpd) in 1441 children <5 years of age with severe anaemia in Malawi.[7]This trial showed that provision of 3 months of chemoprevention with 3 full treatment courses of AL (which is eliminated slowly and provides several weeks of post-treatment prophylaxis), given in-hospital for initial malaria episode and at 1 and 2 months post-discharge, prevented 31% (95% CI 5-50, P=0.02) of deaths or readmissions due to severe anaemia or severe malaria (composite primary outcome) by 6 months post-discharge and 41% by 3 months (95% CI 10-62, P=0.014). The beneficial effect was in addition to the initial effect from the standard AL treatment course provided at discharge and in addition to any protective effect by insecticide treated nets (ITNs).[7] These results are consistent with earlier findings from The Gambia, which showed that in children with severe anaemia, chemoprevention (as monthly IPT with SP or as weekly prophylaxis with pyrimethamine-dapsone) targeted during the malaria transmission season halved the rate of clinical malaria and reduced all-cause hospital readmission by 78% in one trial, and recurrence of severe anaemia by 78% in the other.[3,4] These data are scarce, but indicate that IPTpd in the post-discharge period may potentially provide substantial health benefits.

4.5. PROPOSED CONFIRMATORY TRIAL IN KENYA AND UGANDA

We propose to conduct a confirmatory efficacy trial in Kenya and Uganda to determine the efficacy and safety of 3 months of malaria chemoprevention post-discharge as an innovative potentially cost-effective strategy to reduce all-cause readmissions and deaths in children admitted with severe anaemia. We will call the intervention post-discharge malaria chemoprevention or PMC (instead of IPTpd), to illustrate the similarities with SMC rather than with IPTp in pregnancy. SMC and PMC malaria chemoprevention strategies aim to provide complete prophylaxis during a period of 3 to 4 months, whereas IPTp is given intermittently and allows for reinfections to occur between doses and provides partial protection.

5. JUSTIFICATION FOR THE STUDY

5.1. WHY IS THIS STUDY NEEDED NOW?

In the past two decades, most research on severe anaemia and severe malaria focussed on reducing in-hospital mortality. Our observations suggest that a major, potentially preventable, component of the burden occurs after discharge and that a proactive approach using PMC could offer substantial public health gains. This is a priority area for research since no strategy specifically addresses this high-risk post-discharge period. The study sites include the two original sites that initiated post-

discharge risk research in the 1990s[2,5,7] and hospitals in central and eastern Uganda that have identified a similar high post-discharge burden. These sites are representative for the main epidemiological settings appropriate for this intervention: i.e. moderate to high perennial transmission.

5.2. RATIONALE FOR ANOTHER PLACEBO CONTROLLED TRIAL

Although IPTpd / PMC represents a potential new strategy, it builds on existing strategies used for seasonal malaria chemo-prevention in West Africa and experience with IPT in pregnant women and infants.[15,17]Policy makers and providers are familiar with the IPT and seasonal malaria chemoprevention concept and thus more likely to consider PMC if found to be effective and affordable.[17]However there is no policy or strategy for post-discharge prevention in the countries where the studies are proposed and PMC is unlikely to be considered for policy by WHO or local Governments until these results from Malawi are confirmed in further placebo controlled trials in other countries and until more information is available on the potential delivery mechanism, its cost-effectiveness and potential impact. In addition, the impact on the disease burden and cost-effectiveness of PMC in the presence of other ongoing interventions (notably ITNs, and the recent switch from quinine for the treatment of severe malaria during the in-hospital period to the more effective in-hospital treatment with parenteral artesunate) will need to be assessed to provide the appropriate evidence base for National Malaria Control Programmes (NMCPs) to consider giving priority to this component by adding it to their national programmes rather than other potential focus areas. Furthermore, since the conduct of the original study with AL in Malawi, the long acting DP is now registered and available as second line treatment in both Kenya and Uganda as Duo-Cotecxin®, from Beijing HolleyCotec pharmaceutical Co. Ltd, China (henceforth referred to as HolleyCotec) DP is expected to be more effective than AL because it is longer acting providing approximately 4 weeks of near complete post-treatment prophylaxis. Previous studies in adults showed that when the 3-day courses are taken compliantly on a monthly basis it becomes prophylaxis and prevent almost all infections.[18]

These different components will be addressed by a PMC Consortium funded by the Norwegian Government (see also section 12, page 62). This specific proposal describes the placebo controlled trial. A separate study of potential delivery mechanisms will be conducted in Malawi.

6. HYPOTHESIS

An additional three months of malaria chemoprevention with monthly 3-day treatment courses with DHA-piperaquine (each providing about 4 weeks of post-treatment prophylaxis) provided during the post-discharge period to children recently admitted with severe anaemia is superior to reduce all-cause readmission and mortality rates by 6 months compared with 2 weeks of post-treatment prophylaxis provided by the single course of oral artemether-lumefantrine when given as part of the standard in-hospital care around the time of discharge.

7. AIM AND OBJECTIVES

7.1. AIM

The study is designed to produce the necessary evidence required by WHO to review whether PMC should be recommended as a strategy for the post-discharge management of children with severe anaemia.

7.2. OBJECTIVES

7.2.1. Primary objective

To determine if 3 months of post-discharge malaria chemoprevention with monthly 3-day treatment courses of dihydroartemisinin-piperaquine (DP) (PMC-DP) is safe and superior to the standard single 3-day treatment course with artemether-lumefantrine provided as part of standard in-hospital care in reducing all-cause readmissions and deaths by 6 months in the post-discharge management of children less than 5 years of age admitted with severe anaemia.

7.2.2. Secondary objectives

To determine the cost-effectiveness of PMC-DP compared to current standard of care.

8. DESIGN AND METHODOLOGY

8.1. OVERVIEW STUDY DESIGN

This will be a multi-centre, parallel group, two-arm, placebo-controlled, individually randomized, superiority trial with 1:1 allocation ratio comparing the safety and efficacy of three courses of monthly PMC-DP or placebo post-discharge provided in addition to the standard single 3-day treatment course with artemether-lumefantrine provided as part of standard in-hospital care. Randomisation to PMC-DP or placebo will occur at 2 weeks after enrolment, and PMC-DP treatments will be administered at 2, 6 and 10 weeks. The primary outcome will be a composite of death or all-cause readmission between 2-26 weeks after enrolment. The study will be conducted in Uganda and Kenya, using randomisation stratified by age and study centre. The study will include a total of 1040 children (520 per study arm) less than 5 years of age who have been admitted for all-cause severe anaemia and have successfully completed the standard in-hospital treatment.

8.2. DESIGN CONSIDERATIONS

8.2.1. Rationale for choice of DP for PMC

In order to provide complete prophylaxis for the entire 2-3 month duration that it takes for full hematologic recovery, the optimal regimen would consist of a drug which is sufficiently long acting to prevent the need to administer more often than monthly, as a regimen requiring more frequent administration is likely to result in lower compliance. Of the available antimalarials, SP, mefloquine, and DP have sufficiently long half-lives to be considered.[19]There is high-grade resistance to SP in many parts of Africa and Asia, precluding its use for this purpose in malaria endemic areas in east

and southern Africa. Both piperaquine (PQ) and mefloquine have long half-lives in children, thus providing approximately two weeks longer post-treatment prophylaxis (minimum of 28 days) than artemether-lumefantrine (minimum of 14 days)[20,21] or amodiaquine-artesunate.[19]Furthermore, in an evaluation of amodiaquine (alone, or combined with SP) for IPTp in Ghana, one of the amodiaquine arms was stopped prematurely because the drug was not well tolerated.[22]Mefloquine is also not well tolerated and at treatment doses of 15 or 25 mg/kg is associated with frequent gastro-intestinal side effects, dizziness, and infrequently with serious neuro-psychiatric side effects, which is an important consideration when providing drugs for malaria prevention to asymptomatic and otherwise healthy children.[23,24] Since DP is very effective, well tolerated, and provides 4 to 5 weeks of post-treatment prophylaxis, it is currently the superior candidate drug for IPTp and malaria chemoprevention.

8.2.2. Why in this study population?

The primary study population involves children with all-cause severe anaemia, rather than children with severe malarial anaemia, which was the study population in the previous trial in Malawi.[7]The rationale for this is that subsequent observational analysis in Uganda and western Kenya of data of post-discharge follow-up showed that children admitted with severe anaemia appear to be at increased risk of readmission and death regardless of whether they have evidence of malaria infection at the time of admission. Second, the differentiation between severe anaemia and severe malarial anaemia is not always feasible as it is common practice in many hospitals in sub-Saharan Africa to start parenteral treatment with antimalarials before the laboratory diagnosis of malaria is available. Furthermore the interpretation of malaria diagnostic tests on admission may be complicated in children with a history of recent antimalarial treatment in the days just prior to admission.

8.2.3. Efficacy vs effectiveness

This is an efficacy trial, rather than an operational effectiveness study and each treatment course will be provided by study staff directly or by village based community health volunteers(CHVs) coordinated by study staff. As a minimum the first dose of each course will be observed. Where feasible, doses on day 2 and 3 will also be given under supervision, or compliance will be verified by home visits or contacting caretakers and/or CHVs by mobile phone.

8.2.4. Why this composite primary outcome?

Use of clinical malaria as primary outcome would require a smaller study, however the composite severe outcome is used because it is more likely to drive policy. A composite outcome rather than a single severe outcome, such as death, is used to reduce sample size requirements.

8.2.5. Rationale for assessment by 6 months after enrolment

The period 2-26 weeks, instead of 0-26 weeks is used for the primary efficacy analysis because children will only be randomized at 2 weeks. Prior to 2 weeks, all children, including those in the placebo arm, receive a 3-day course of AL as part of standard in-hospital care, which will be started while they are still admitted, and completed at home after discharge. The duration of post-treatment prophylaxis with AL is about 2 weeks, thus no differential effect is anticipated until children receive their first study specific intervention at 2 weeks post-discharge. A total of 26 weeks

(6 months) follow-up is included to capture whether the benefits achieved by 14 weeks (if any), when protective drug levels have waned in most children are sustained for a further 3 months.

8.2.6. Rationale for testing of malaria and anaemia associated genes

The study includes pheno/genotyping for the main polymorphisms that are known to provide protection against severe forms of malaria, including the haemoglobinopathies sickle cell trait/disease and thalassaemia, as well as G6PD deficiency, a hereditary enzyme deficiency. These are among the most common polymorphisms known. The carriers of these genes are partially protected from the adverse events associated with malaria, and are less likely to develop severe malaria illness, but some children may be more prone to develop chronic or severe anaemia. Unequal distribution by chance at randomisation of children with or without these genes could result in important bias of the study results (confounding). Similarly, the response to the treatment groups may differ between children with normal haemoglobin or normal enzyme-activity and children who carry the gene for haemoglobinopathies or G6PD deficiency. This can result in effect modification. These polymorphisms are common (>20%) and because the strength of the protective effect against severe malaria is considerable (70-90%), even small difference in distribution of these factors, or small modifications of the treatment effect, may have major implications on the observed effect. For a correct interpretation of the study results it is thus important to know the distribution of these polymorphisms between the study groups. For a description of the laboratory techniques see section 9.4.1, page 48.

8.2.7. Rationale for testing parasite genetics and resistance-associate genes

This study also includes genetic examination of the Plasmodium falciparum parasites infecting the enrolled patients. Parasite genetic material will be obtained from the same blood sample as those required for the clinical follow-up. Whole blood will be stored on filter paper blood spots for later processing. Genetic evaluation of the parasite can provide important insight into the degree of infection and importantly monitor for developing resistance due to ongoing drug pressure. First by examining the parasite genetic diversity, comparing baseline parasite composition to treatment and post-treatment complexity of infection (number of strains). The complexity of infection as well as the presence or absence of infection provides a gross measure for the efficacy of the PMC regimen in reducing the parasite burden. Second we will focus our genetic studies to monitor for signs of drug resistance. Artemisinin-based combination therapies have demonstrable parasite resistance in South East Asia and there is great concern that resistance will spread to or develop within Africa.[25] Importantly we will monitor for signs that drug pressure is selecting for resistant parasite strains. Genes associated with parasite drug resistance will be sequenced including those implicated in artemisinin resistance (kelch K13), piperaquine (pvcrt), and lumefantrine (pfmdr1). Compared to baseline parasites we will determine if any mutations within these known drug resistance candidates have an increased frequency within the treated individuals. For a description of the laboratory techniques see section 9.4.1, page 48.

8.3. STUDY SETTINGS

The study will be conducted in approximately 4 hospitals in western Kenya and at least 3 in Uganda, each located in areas with moderate to intense malaria transmission.[5,6]Below is a list of suggested first choice and back-up hospitals where the study will be conducted. The list is not exhaustive and

subject to change following exploration and review of the above mentioned eligibility criteria and participant recruitment rates.

8.3.1. Kenya

Approximately eight hospitals in western Kenya will be chosen from a list of Government or private (e.g. mission) hospitals in the previous Western and Nyanza Provinces, based on patient numbers, available infrastructure and malaria transmission and planned malaria control interventions in the catchment area.

8.3.1.1. Suggested primary hospitals and neighbouring satellite clinics

1. *Siaya County Referral Hospital:* Siaya Town, Siaya County, western Kenya. The 240-bed hospital is located in an area of high perennial malaria transmission in the lake region and serves mainly a rural population. This s the main public hospital in the district, with a total of 260 beds, including 60 beds in the paediatric unit.[26]
2. *Kisumu County Referral Hospital:* 195-bed government facility located in Kisumu county, in Kisumu town, in an area of moderate
3. *Jaramogi Oginga Odinga Teaching and Referral Hospital:* Government owned 467 bed capacity hospital located in Kisumu County in Kisumu town in an area of moderate transmission of malaria.
4. *Migori County Referral Hospital:* Is a 180 bed capacity hospital 3 hours away from Kisumu located in Migori county in Migori town, South western part of Kenya in an area with moderate –high transmission of malaria
5. *Ahero County Hospital:* A 50 bed government facility located in Ahero town, Kisumu County, western Kenya, located 20 Km, approximately 30 min drive from Kisumu.
6. *Rabuor sub County Hospital:* A 30 bed government facility located in Rabuor township, Kisumu County, western Kenya, located 10 Km, approximately 15 min drive from Kisumu.
7. *Kombewa County Hospital:* A 90 bed government facility located in Kombewa township, Kisumu County, western Kenya, located 30 Km, approximately 40 min drive from Kisumu.
8. *Pastor Machage Memorial Hospital, Migori:* A 115 bed Private facility located in Migori town, Migori County (formerly South Nyanza). The facility is ~200Km (3-hour drive from Kisumu)

8.3.1.2. Backup Hospitals and neighbouring satellite clinics

9. *Homa Bay District Hospital:* This is a 280 bed government facility in Homa Bay County, appr. 2 hours drive (110km) from Kisumu, in an area of intense malaria transmission
10. *St Mary's Hospital, Mumias*: This is a 200 bed mission hospital located in Mumias town, 1.5 hours drive from Kisumu, in an area with moderate malaria transmission
11. *Mukumu Mission Hospital:* This is a 228-bed mission hospital located on the road between Kisumu and Kakamega town in an area with moderate malaria transmission
12. *Kisumu District Hospital:* 195-bed government facility located in Kisumu town, in an area of moderate malaria transmission.
13. *Busia District Hospital:*185-bed government facility, located in Busia town at the Kenyan-Ugandan border, Busia County, Western Province. Distance to Kisumu 110km, appr. 2 hours drive. Intense malaria transmission.
14. *Nyamira District Hospital:* 203-bed government facility, located in Nyamira County, 1.5 hours drive from Kisumu.

15. *Rachounyo sub county hospital:* Government owed 80 bed capacity hospital located in Homabay County, in Oyugis town, in an area of moderate malaria transmission. Distance ~80 KM (1 hour drive from Kisumu)
16. *Bondo District Hospital:* This 50-bed hospital is located in Bondo town, Siaya County, western Kenya, located 50 kilometres west of Kisumu in an area with intense malaria transmission.
17. *St. Joseph's Mission Hospital-Ombo:* A 150 bed Private facility, owned by the Catholic diocese of Homa Bay, located in Migori town, Migori County (formerly South Nyanza). The facility is ~200Km (3-hour drive from Kisumu)
18. *Nyakach sub County Hospital:* A 40 bed government facility located in Pap Onditi township, Kisumu County, western Kenya, located 35 Km, approximately 45 min drive from Kisumu.

8.3.2. Uganda

8.3.2.1. Primary hospitals and neighbouring satellite clinics

1. *Tororo general Hospital*: A 200-bed public hospital located in Tororo district in the eastern part of Uganda, and area with intense perennial malaria transmission.
2. *Hoima Regional Referral hospital:* A Government owned regional referral hospital located in the mid-western part of the country serving an area of intense malaria transmission.
3. *Jinja Regional Referral Hospital*: A regional referral hospital located in the central region serving an area along the shores of Lake Victoria with moderate and seasonal malaria transmission.
4. *Masaka Regional Referral Hospital*: A 350=bed hospital located in the south-western part of the country. It serves the people living along the shores of Lake Victoria; this is an area of seasonal malaria transmission.

8.3.2.2. Backup hospitals and neighbouring satellite clinics

5. *Lira Regional Referral Hospital*: A 350-bed referral hospital in the northern part of Uganda serving 8 districts in the Lango sub-region including the Apac district. This is an area with intense malaria transmission. Recruitment will be expanded to this hospital if recruitment in the other hospitals is slower than anticipated.
6. *Soroti Regional Referral Hospital*: Soroti Hospital is a 200+ bed hospital in the town of Soroti, in Soroti District, in eastern Uganda.
7. *Gulu Regional Referral Hospital*: Gulu Hospital is a 250-bed hospital in Gulu town, northern Uganda.
8. *Mubende Regional Referral Hospital*: A regional referral hospital located in the central region, an area with moderate and seasonal malaria transmission
9. *Arua Regional Referral Hospital*: A regional referral hospital located in West-line sub-region, an area with high to moderate malaria transmission
10. *Iganga general Hospital*: A 200-bed public hospital located in Iganga district in the eastern part of Uganda, along the shores of Lake Victoria an area with high to moderate malaria transmission.
11. *Kamuli Mission Hospital*: A 200-bed catholic- mission hospital located in Kamuli district in the eastern part of Uganda, along the shores of Lake Kyoga an area with moderate malaria transmission.

8.4. ELIGIBILITY CRITERIA

For a description of the pre-study screening procedures see section 8.7.2, 'Visit 1: Pre-screening', page 37.

8.4.1. Eligibility criteria for Pre-Study Screening

8.4.1.1. Inclusion criteria for enrolment into pre-study screening period

1. Haemoglobin <5.0 g/dl or PCV < 15%, or requirement for blood transfusion for other clinical reasons on or during admission to the hospital
2. Aged less than 59.5 months
3. Body weight ≥5 kg
4. Resident in catchment area

8.4.1.2. Exclusion criteria for enrolment into pre-study screening period

1. Recognised specific other cause of severe anaemia (e.g. trauma, haematological malignancy, known bleeding disorder)
2. Known sickle cell disease
3. Anticipated to reach the 5th birthday (60 months of age) within 2 weeks from enrolment (i.e. prior to randomization)
4. Child will reside for more than 25%of the 6 months study period (i.e. 6 weeks or more) outside of catchment area

- Hereditary polymorphisms, other than sickle cell disease, that are known to provide protection against severe forms of malaria but may also cause severe anaemia, including thalassaemias and G6PD deficiency are not exclusion criteriae as no specific malaria control policies are recommended for these groups (by contrast to sickle cell disease).
- HIV infection and cotrimoxazole prophylaxis are not exclusion criteria.

8.4.2. Eligibility criteria for enrolment into study

For a description of the study procedures criteria see section 8.7.3 , 'Visit 2: Screening interview and consent', page 38.

8.4.2.1. Inclusion criteria for enrolment:

1. Fulfilled the pre-study screening eligibility criteria
2. Aged < 59.5 months
3. Clinically stable, able to take oral medication
4. Subject completed blood transfusion(s) or became clinically stable without transfusion
5. Able to feed (for breastfeeding children) or eat (for older children)
6. Absence of know cardiac problems
7. Provision of informed consent by parent or guardian

8.4.2.2. Exclusion criteria for enrolment:

10. Previous enrolment in the present study
11. Known hypersensitivity to study drug
12. Sickle cell disease

13. Use or known need at the time of enrolment for concomitant prohibited medication (see section 8.5.7.2, page 35) during the 14 weeks PMC treatment period (see section 8.5.7.2, page 35).
14. Ongoing or planned participation in another clinical trial involving ongoing or scheduled treatment with prohibited medicinal products or active follow-up during the course of the study (6 months from enrolment)
15. A known need at the time of enrolment for scheduled surgery during the subsequent course of the study (6 months from enrolment)
16. Suspected non-compliance with the follow-up schedule
17. Know heart conditions, or family history of congenital prolongation of the QTc interval.
18. Taking medicinal products that are known to prolong the QTc interval[1]

8.4.3. Eligibility criteria for randomisation into study (at 2 weeks)

8.4.3.1. Inclusion criteria for randomisation:

1. Fulfilled enrolment eligibility criteria and was enrolled during recent admission
2. Aged <60 months
3. Still clinically stable, able to take to oral medication, able to feed (for breastfeeding children) or eat (for older children) and able to sit unaided (for older children who were already able to do so prior to hospitalisation)

8.4.3.2. Exclusion criteria for randomisation:

1. Used DP since enrolment
2. Use or known need at the time of randomisation for concomitant prohibited medication (see section 8.5.7.2, page 35) during the 14 weeks PMC treatment period (see section 8.5.7.2, page 35).
3. Enrolled, or known agreement to enrol into another clinical trial involving ongoing or scheduled treatment with medicinal products during the course of the study (6 months from enrolment)
4. A known need at the time of randomisation for scheduled surgery during the subsequent course of the study (6 months from enrolment)
5. Suspected non-compliance with the follow-up schedule
6. Withdrawal of consent since enrolment

- Readmission or re-transfusion in the 2 week period since enrolment or on the day of randomisation screening is not an exclusion criteria for randomization but require a delay of randomisation so that a minimum of 12 days and a maximum of 21 days have passed since the most recent discharge if artemether-lumefantrine was provided around the time of (re)discharge, provided the patient has clinically recovered and is stable and fulfils the randomisation eligibility criteria on the re-scheduled day of randomisation.

[1] See Section 8.5.7.2 “Concomitant and prohibited Medications”, page 34

- Fever (≥37.5°C) on the day of randomisation screening is not an exclusion criteria for randomization but requires a delay of randomisation. Febrile children will be screened for malaria using RDTs or malaria smears, and RDT or smear positive children will be treated with a repeat course artemether-lumefantrine. They will be eligible for randomisation when a minimum of 12 days and a maximum of 21 days have passed since the first day of the repeat course of artemether-lumefantrine, provided the patient has clinically recovered and is stable and fulfils the randomisation eligibility criteria on the re-scheduled day of randomisation.

8.5. INTERVENTIONS

8.5.1. Standard in-hospital and post-discharge care (not study specific or study related)

All care provided prior to and following enrolment of the patient in the study (at convalescence) will not be part of the study and will be per local (hospital) or national guideline with the exception of the full 3 day course of artemether-lumefantrine (AL) as described below. All details of non-study specific care provided by the hospital staff will be recorded in the study forms, including all drugs and dosages prescribed. The hospital teams providing care will be provided with updates of the latest national and local guidelines for in-hospital care.

Blood transfusion and malaria treatment: The standard in-hospital care for severe anaemia includes a blood transfusion (20mL/Kg whole blood or 10 mL/Kg Packed Red cells). In addition, patients suspected to have severe malarial anaemia also receive antimalarial treatment. In patients not able to take oral medication this consists of parenteral artesunate (2.4 mg/Kg at time 0, 12 and 24 hours), followed by a full 3 day course of artemether-lumefantrine (AL) once the patient is able to switch to oral medication. In some instances, when artesunate is not available, patients may receive parenteral quinine (10mg/kg every 8 hours) or i.m artemether. In those children who are able to take oral medication throughout, the 3-day course of AL is given without prior parenteral treatment with artesunate.

Antibiotics are commonly used during the in-hospital period and will be documented, but will not be part of the study intervention.

Iron and folate supplementation will not be part of the medication provided by the study until the time of randomisation at 2 weeks post-discharge. Until then, the use of haematinic supplementation is at the discretion of the treating clinicians. However, the hospital staff will be reminded that iron supplementation is not required during the first two weeks because the blood transfusion provided as part of the standard in-hospital care will contain adequate amounts of iron for the initial post-discharge period. See section 8.5.2.7, page 31 for details about iron supplementation during the PMC intervention period (i.e. from the first PMC dose at 2 weeks onwards).

HIV testing, see section 8.7.3.5, page 38.

8.5.2. Trial Medication and Interventions

8.5.2.1. PMC intervention schedule

Children will be randomised to one of the 2 treatment groups: Children in both arms will receive standard in-hospital care for severe (malarial) anaemia (blood transfusion, with or without antimalarial treatment with artesunate IV/IM). They will then all receive a 3-day course of artemether-lumefantrine (Coartem®, Novartis Pharmaceuticals) (AL) regardless of whether they were admitted with severe malarial anaemia or severe anaemia without evidence of malaria. At 2 weeks post-discharge, surviving children will be randomized to receive a standard 3-day course of DP at 2, 6, and 10 weeks post-discharge or an identical placebo regimen (Table 1, page 14). If children receive AL for malaria during any unscheduled out-patient visit or during a readmissions, the PMC schedule will be adapted accordingly such that any next course of DP is given at least 2 weeks after the start of the last course of AL; e.g. if a child was due to receive the 2nd course of DP at 6 weeks post-discharge, but is treated for malaria with AL during an unscheduled visit at 5 weeks, then the 6-week course of DP (or placebo) is provided in week 7 instead of week 6, and the subsequent course in week 11 instead of week 10.

8.5.2.2. Artemether-lumefantrine

The study will use the GMP formulation of Artemether-lumefantrine: Coartem®, Novartis Pharmaceuticals. Artemether-Lumefantrine will be dosed along with a small amount of food as per national guidelines. The recommended treatment is a 6-dose regimen over a 3-day period. The standard tablet or the dispersible tablets containing 20 mg of artemether and 120 mg of lumefantrine will be used and doses according to bodyweight following WHO dosing recommendations as provided in the latest WHO malaria treatment guidelines. The dose regimen will be reviewed and potentially updated if and when WHO updates their dose recommendations. The dosing is based on the number of tablets per dose according to pre-defined weight bands and the current recommendations is 5–14 kg: 1 tablet; 15–24 kg: 2 tablets; 25–34 kg: 3 tablets; and > 34 kg: 4 tablets, given twice a day for 3 days (Table 3). This extrapolates to 1.7/12 mg/kg body weight of artemether and lumefantrine, respectively, per dose, given twice a day for 3 days, with a therapeutic dose range of 1.4–4 mg/kg of artemether and 10–16 mg/kg of lumefantrine.

Table 3: Artemether Lumefantrine weight based dosing schedule

**Weight in Kg**	**Number of paediatric tablets of Coartem® per dose**					
	**Day 1**		**Day 2**		**Day 3**	
	1st dose	8 hours	24 hours	36 house	48 hours	60 hours
**5 to <15**	1	1	1	1	1	1
**15 to<25**	2	2	2	2	2	2
**25 to ≤34**	3	3	3	3	3	3
**>34**	4	4	4	4	4	4

In some children, artemether-lumefantrine will have been provided by the hospital staff prior to enrolment into the study. This is likely in children who are severely anaemic, yet are able to take oral medication and do not require parenteral antimalarials. The number of doses and the brand of artemether-lumefantrine received prior to enrolment will be documented by the study staff on

enrolment to the study (section 8.7.3, page 38), and the subsequent number of doses of trial artemether-lumefantrine will be adjusted accordingly to ensure that the child receives a cumulative total of six doses over 3-days (section 8.7.4, page 39).

8.5.2.3. Dihydroartemisinin-piperaquine for post-discharge malaria chemoprevention

The study will use the following formulation of Dihydroartemisinin-piperaquine: Eurartesim® from Sigma Tau, Italy, another brand of DP similar to the Duo-Cotecxin brand approved by the Kenyan and Ugandan regulatory authorities. Eurartesim is a co-formulated tablet containing 40 mg dihydroartemisinin and 320 mg piperaquine phosphate or as 20/160 (paediatric formulation).

8.5.2.4. Dihydroartemisinin-piperaquine dosing regimen

Dosing will be by bodyweight according to the following schedule recommended by WHO's Guidelines for the treatment of malaria, 3rd edition from April 2015 (Table 4).

Table 4: Dihydroartemisinin-piperaquine weight-based dosing schedule

**weight in Kg**	Daily dose (mg)		Tablet strength and number of tablets per dose
	Piperaquine	DHA	
**5 to <8**	160	20	1 x 160 mg / 20 mg tablet <u>or</u><br>½ x 320 mg / 40 mg tablet
**8 to <11**	240	30	1.5 x 160 mg / 20 mg tablet <u>or</u><br>¾ x 320 mg / 40 mg tablet
**11 to <17**	320	40	1 x 320 mg / 40 mg tablet
**17 to <25**	480	60	1.5 x 320 mg / 40 mg tablet
**25 to<36**	640	80	2 x 320 mg / 40 mg tablet

If the paediatric strength tablets are not available, the full tablet strength can be used to provide the equivalent dose in mg (i.e. ¼ tablet and ½ tablet of 320 / 40 mg tablets for the first two weight bands).

8.5.2.5. Dihydroartemisinin-piperaquine administration

The first dose will be administered crushed and dissolved in water in the clinic or the home of the participant under direct observation. The remaining two doses will be given to take at home during the following two consecutive days. Participant's caretakers will be advised to give the drug to the child at about the same time each day with water. Should a caretaker forget to give the drug dose at the set time, they will be advised to take it as soon as this is realised and to continue the recommended regimen until the dose is completed.

Participants who take DP or placebo in the clinic will be observed for at least 60 minutes. Should a participant vomit within 30 minutes of receiving the treatment drug, the full dose will be re-administered. Should a participant vomit within 30-60 minutes of drug intake, half dose will be re-administered. Repeat dosing will be attempted once and if the second dose is vomited, the participant will receive artemether-lumefantrine. Caretakers will be instructed to inform the study team the same day for a replacement dose if a participant vomits the treatment at home

To minimise QTc prolongation, Sigma Tau, Italy, the manufacturer of this brand of DP (Eurartesim®), advises patients to take the first day's dose approximately three hours after meals as fatty food can increase the absorption of piperaquine. However, overall studies show that DP when given at the

standard dose over 3 days is well tolerated and can be given safely with small amount of food, such as a biscuit.

Recently a review was completed for WHO to evaluate the safety of DP when used for IPT.[27] Across all studies, including 7 trials [28,29,30,31,32,33,34], monthly DP was associated with a significantly lower risk of SAEs compared to placebo, daily trimethoprim-sulfamethoxazole, or monthly SP. Overall 12 deaths were reported among those exposed to DP and 16 among those exposed to comparator therapies, with no studies reporting any sudden or unexplained deaths.[27]

One trial in children 6m to 24m of age in Uganda, included monthly DP for up to 18 monthly courses.[34] As part of this trial detailed sub-studies of the effect of DP on cardiac repolarization were conducted in 26 children.[34] A total of 183 ECGs were conducted; all of the follow-up ECGs had a QTc < 450msec with a mean QTc = 396msec (range 278-444, SD 31.3). There were no differences in the mean (SD) QTc intervals measured 4-6 hours after the 3rd dose (i.e. peak piperaquine levels) for children who had been prescribed 3-5, 6-10, or 11-18 prior doses of DP: Mean (SD)QTc 405 (26), 388 (33) and 396 (33), respectively. Thus this nested cardiac monitoring study shows that monthly courses of DP is not associated with a trend toward increasing QTc prolongation with increasing number of DP courses. DP also provided superior efficacy compared to placebo or comparators in preventing any parasitaemia in participants (pooled Incidence Rate Ratio 0.22 (95% confidence interval 0.11-0.33).[27]These limited data on repeat DP exposures suggest that repeat 3-day courses of DP given at monthly intervals over 3 month may be safe and effective and a good option for IPT.

8.5.2.6. Placebo DP

Placebos for DP will be manufactured by Sigma Tau, Italy. The dosage regimen for DP-placebo will be identical in number of tablets per day and timing of the dose to that of the active DP product. The drug administration procedures will also be identical to that for the active drugs.

8.5.2.7. Iron and folate supplementation

All children in both arms, regardless of Hb level, will receive a standardized prophylactic dose of iron supplementation (about 2 mg/kg) from the time of randomisation onwards for a period of about 4 weeks (i.e. until the next dose of PMC-DP is due at 6 weeks). Iron can be given as mono-therapy or as part of the fixed-dose formulation with folic acid.

8.5.3. Procedures for Drug handling & Accountability

8.5.3.1. Preparation and packaging

All subject specific study drugs will be crushed and stored in small opaque medicinal containers provided by the sponsor, according to the subject's bodyweight recorded at randomisation. This will be done by a pharmacy assistant who will be unblinded to the study. The full course will be prepared in one session, i.e. 3 containers for a full course of DP or Placebo and will be kept in a subject specific study drug box after preparation until dispensing. Packaging used for all treatment arms will be identical and labelled blinded to the contents.

Just prior to each drug administration, the drugs will be suspended in the opaque container by adding flavoured syrup.

8.5.3.2. Labelling of trial drug

Labelling will be in English and the local language and in accordance with local regulations for each participating country, which will include the name of the study, name and study identification number of the participant, drug dose and may also include usage directions and staff contact number, precautionary measures to be observed when taking the drug.

8.5.3.3. Product Storage

All study drugs will be stored in a secure area with access limited to Investigator and authorised study site personnel, and under appropriate storage conditions. A description of the appropriate investigational product-specific storage conditions are specified on the investigational product pack label.

8.5.3.4. Product accountability

The site-PI will be responsible for establishing a system for the correct handling of study drug to ensure that:

1. Deliveries of study drug from the sponsor are correctly received by a responsible person (e.g. pharmacist assistant)
2. Accurate records are maintained for the receipt of study drug, for the dispensing of study drug to subjects and for returned drug.
3. Certificates of delivery and return must be signed preferably by the investigator or authorised personnel and copies retained in the investigator file.
4. Study drug is to be handled and stored safely and properly and in agreement with the given storage instructions.
5. The study drug is to be prescribed only by the principal investigator, co-investigators or study site personnel authorised to do so by the principal investigator.
6. Study drug is dispensed only to study subjects in accordance with the protocol.
7. Subjects must return all unused medication and empty containers to the investigator.
8. At the end of the study, delivery records must be reconciled with records of usage and returned stock. Any discrepancies must be accounted for in writing.
9. Once accounted for any returned and unused study treatment at the site will be returned to the sponsor for destruction or destroyed locally upon agreement with the sponsor. Drug destruction certificates will be issued that refers to the subject study numbers for subject specific medication that was destroyed.

8.5.3.5. Pharmacist assistant/dispenser

All efforts will be made for the preparation, packaging and labelling of the blinded study drug to be performed and documented in accordance with Good Manufacturing Practice (GMP). The sponsor will provide the pharmacist assistant with written instructions and GMP training on the preparation, packaging and labelling procedures.

8.5.4. Removal of Patients from Therapy or Assessment

Patients can discontinue from the study for any one of the following reasons.

1. Screening error resulting in incorrect enrolment (found that subject did not meet required inclusion / exclusion criteria)

2. Withdrawal of consent at any stage or subject not willing to continue in the study / voluntary discontinuation by the subject
3. Suspected or confirmed allergic reaction to the study drug (removal from therapy only)
4. Safety reasons as judged by the investigator, study safety monitor or Data Monitoring and Ethics Committee (DMEC) (removal from therapy only)
5. [Cardiac monitoring sub-study] At randomization, clinically significant ECG abnormality, defined as QTc interval of >450 ms or evidence of other clinically significant ECG abnormalities, including arrhythmias, ischemia, or evidence of heart failure.
6. [Cardiac monitoring sub-study] After randomization in children who have started receiving PMC; onset of grade 2-4 ECG abnormalities, confirmed after repeat testing.
7. Other

The parents or guardian of subjects who discontinue from the study treatment or from the study entirely will always be asked about the reason(s) for their discontinuation and the presence of adverse events. If a subject discontinues it should be established whether the subject:

1. Discontinues the study treatment, but continues their consent for the data capture up to that point, and to continue follow-up. These subjects will be considered 'off study drug/on study' and where feasible will follow the same schedule of events as those who continue the study intervention, except any adherence assessment. All of these children will be followed until study end at 6 months from enrolment.
2. Discontinues all future activities in the study, but continues their consent for the data captured up to that point to be used in the research
3. Discontinues all future activities in the study and withdraws consent for any data captured to be used for the research

Every effort will be made to follow-up patients who discontinue due to drug related adverse events in order to determine the final outcome. If a subject discontinues due to drug-related adverse events, all the assessments will be conducted that would have been carried out at the next scheduled visit at 6 month (unless consent is withdrawn). This will be recorded in the Case Record Forms (CRFs). The study drug will be returned by the subject. Subjects that have discontinued the study prematurely will not be replaced.

8.5.5. Discontinuation from storage of blood for future studies

If a subject discontinues it will also be established whether the subject:

1. Continues their consent for long term storage of the blood sample
2. Withdraws consent for long-term storage (for future studies and for genetic testing that is part of the main study protocol, but that may not yet have been conducted for that individual child) before anonymization of the dataset has occurred.

When a subject's consent for long-term storage is withdrawn, the stored sample will be destroyed and the withdrawal noted in the CRF. If the request is received after the dataset has been anonymised, the stored sample can no longer be withdrawn.

8.5.6. Adherence to study intervention protocol and strategies for retention

8.5.6.1. Adherence to study protocol and medication

At 2, 6 and 10 weeks children will be visited at home or asked to come to the clinic to receive the PMC with DP or placebo. For the home-treatment courses, drug administration of the first daily dose will be given by a study staff or home visitor under direct supervision (day 0). The study staff will instruct the mother to administer the second and third dose around the same time the next two days (days 1 and 2). Care takers of study participants and home visitors will also be reminded through mobile phone contact to take the assigned tablets for the second and third dose. On the last day, some caretakers will be revisited at home by spot checks and asked whether and when the second and third dose was administered. All information will be recorded on the appropriate sections of the CRF.

Subjects judged to be non-compliant may continue in the study but should be counselled on the importance of taking their study medication as prescribed.

8.5.6.2. Strategies for retention

During screening, parents will be asked whether the child will travel out of the study area for an extended period during the follow-up period. Those who plan 1 month or more away from the study catchment area will be excluded from enrolment. The 'study catchment area' will be defined for each study site before the start of the study. All participants will be reimbursed for transportation costs to and from the clinic.

Detailed directions to the children's homes as well as contact information, including cell phone information, will be recorded prior to discharge. At 18 weeks after enrolment, parents or caretakers will be called or visited at home if no mobile phone contact is possible, to find out about the wellbeing of the study subject as well as remind them to come to the study clinic for the last scheduled visit at week 26. If children do not return for scheduled follow-up visits, the study team will call them and ask them to come to the clinic for evaluation, offering transport reimbursement, or may visit their house to help arrange transport to the clinic if they are willing to come to the clinic, or, alternately, a study staff may go to their home for clinical evaluation and to assess if they still wish to participate in the study.

The caretaker and participant's travel costs will be reimbursed as described in more details in section 11.9, Expenses reimbursement and incentives, page 61.

8.5.7. Prior and concomitant therapy

All concomitant medications taken during the study will be recorded in the appropriate sections of the CRF with indication, dose information, and dates of administration.

8.5.7.1. Permitted Medications during follow up period

During the treatment and follow-up phase of the study if a subject is diagnosed with malaria the investigator will prescribe antimalarial treatment based on the severity of the malaria illness as indicated in Table 1: Study treatment and phases, page 14.

Use of cotrimoxazole (which has some antimalarial properties) treatment or prophylaxis is not considered prohibited as short-courses are commonly prescribed for the treatment of bacterial infection. Daily cotrimoxazole prophylaxis is used by HIV-infected and exposed children.

8.5.7.2. Concomitant and prohibited Medications

Participants will be counselled to avoid concomitant and prohibited medications, specifically antimalarial drugs not prescribed within the trial protocol, or drugs that may be associated with QTc prolongation.

Prohibited medication

- Antimalarials not prescribed by the study: Chloroquine, halofantrine, mefloquine
- Diuretics (hydrochlorothiazide, furosemide)
- Drugs known to prolong the QT interval
    - Antimicrobials:
        - macrolides (e.g. erythromycin, clarithromycin, azithromycin, roxithromycin),
        - fluoroquinolones (e.g. ciprofloxacin, norfloxacin, levofloxacin, moxifloxacin, sparfloxacin)
        - pentamidine
    - Antifungals: ketoconazole, fluconazole, Itraconazole, posaconazole, voriconazole, caspofungin
    - Antiretrovirals: ARVs, specifically: indinavir, nelfinavir, atazanavir, saquinavir
    - Antiarrhythmic agents (e.g. amiodarone, sotalol)
    - Non-sedating antihistamines (astemizole, terfenadine)
    - Antipsychotics (neuroleptics): Haloperidol, Thioridazine
    - Antidepressants: Imipramin, Citalopram, Escitalopram
    - Antiemetics: Domperidone, Chlorpromazine, Ondansetron

Randomised participants who take prohibited medications resulting in the premature cessation of the study intervention, will remain in the trial and will be included in the primary, intention-to-treat analysis, but excluded from the per-protocol analysis.

8.6. OUTCOMES

8.6.1. Primary efficacy outcome

All-cause deaths or all-cause re-admissions by 26 weeks from randomization (composite primary outcome).

8.6.2. Key secondary efficacy outcome

8. Readmission due to severe malaria (defined as any treatment with parenteral quinine or artesunate, or presence of severe anaemia and treatment with oral antimalarials) by 26 weeks from randomization
9. Readmissions due to severe anaemia (defined as Hb <5g/dL or PCV <15% or requirement for blood transfusion based on other clinical indication) by 26 weeks from randomization
10. Readmission due to severe malarial anaemia (severe anaemia plus parenteral or oral antimalarial treatment) by 26 weeks from randomization

11. Readmission due to severe anaemia or severe malaria (composite outcome) by 26 weeks from randomization
12. All-cause mortality by 26 weeks from randomization
13. All-cause hospital readmission by 26 weeks from randomization
14. Clinic visits because of smear of RDT confirmed non-severe malaria by 26 weeks from randomization

8.6.3. Other secondary efficacy outcomes

15. Readmission due to severe malaria-specific anaemia (severe anaemia plus parenteral or oral antimalarial treatment and parasite density >5000/microlitre) by 26 weeks from randomization
16. Readmission due to severe disease other than severe anaemia and severe malaria by 26 weeks from randomization
17. Non-severe all-cause sick-child clinic visits by 26 weeks from randomization
18. Non-malaria sick child clinic visits by 26 weeks from randomization
19. Malaria infection at 6 month
20. Hb at 6 months
21. Any anaemia (Hb<11 g/dL), mild anaemia (Hb 8.0-10.99 g/dl) moderate anaemia (Hb 5.0-7.99 g/dL) and severe anaemia (Hb<5 g/dL) at 6 months
22. Weight-for-age, height-for-age, and height-for-weight Z-scores (standard deviation [SD] scores of reference population) at 6 months

8.6.4. Tolerability and safety

6. Serious adverse events, excluding primary and secondary efficacy outcomes, by 26 weeks from randomization
7. Serious adverse events within 7 days after the start of each course of PMC, excluding primary and secondary efficacy outcomes.
8. Adverse events by 26 weeks from randomization
9. Adverse events within 7 days after start of each course of PMC.
10. QTc prolongation measured by electro cardio gram (ECG)4-6 hours after 3rd dose of each course

8.6.5. Economic evaluation outcomes

1. Patients costs of receiving the intervention
2. Patients costs related to treatment of the primary disease, readmission or death
3. The costs of the health care system of providing the intervention
4. The costs of the health system of treating the primary disease and anaemia, as well as treatment of readmissions or costs related to fatalities

For more detailed definitions of intervention costs outcomes, see section 9.8, 'Economic Evaluation sub-Study', page 55.

8.7. PARTICIPANTS TIMELINE

8.7.1. Overview Study Phases

The study plan and schedule of assessment is provided in Table 1: Study treatment and phases, Page 14 and Table 2: Study Design and Schedule of Assessment, Page 15, and Figure 1: Flow of Participants, page 17.

It consists of an in-patient pre-study screening period while the patient is acutely ill (approximately -4 to 0 days before enrolment) followed by a screening and enrolment visits during the convalescence phase in the hospital, just prior to discharge. It will be during this enrolment visit when the children become formal study participants (t=0). They will also be provided with artemether-lumefantrine (AL) (Coartem®) during this time (both arms) as soon as the child can take oral medication, taking any pre-enrolment doses of AL into account that may already have been provided by the hospital as part of standard care. The child will then be seen again at 14 days, when they will be allocated to one of the two study arms and receive their first course of active PMC or placebo. The subjects will be visited again at home at 6 and 10 weeks after enrolment to be administered the second and third course of PMC. These home visits will be for drug administration purposes and vital registration only, not for clinical assessment. The PMC period ends at 14 weeks, i.e. 4 weeks after the third PMC course. They will then be followed up for an additional 12 weeks through passive follow-up and then seen at 26 weeks for an end of study assessment. Subject's parent or guardian will be instructed to return his/her child to the study clinic for evaluation free of charge at any time their condition warrants medical attention during the 26 weeks follow-up period after discharge.

8.7.2. Visit 1: Pre-screening

Parents / guardians of children who fulfil the pre-screening eligibility criteria (see section 8.4.1, page 26) will be informed about the study by the hospital or study staff. Consent will not be obtained at the acute stage of the illness but a few hours or days later when the child has recovered and is able to take oral medication (see section8.7.3, below). This provides a time window for the parents or guardian to reflect on the study and discuss it with family members and study staff.

Pre-study screening will be done by hospital staff or study staff. No study specific information or study samples will be collected in this pre-study screening period. However, study staff will be asked to keep any leftover blood volume of routine samples that would otherwise be discarded (e.g. for blood-group typing and cross-matching) in the fridge until consent is obtained in visit 2 (see below), including malaria smears taken on enrolment. The role of the study team during this period is to review the diagnosis and ensure that the potential study participants get standard care for severe malarial anaemia.

Each pre-screened subject will be assigned a pre-screening number in sequential order by the hospital regardless of whether they fulfil the pre-screening eligibility criteria. Data will be recorded on a pre-screening log that will be kept in the investigator's site file. One pre-screening log will be kept per hospital. This record will be used to report how many patients were pre-screened and how many were eventually recruited in the study to establish that the study population was selected without bias. This screening log will not contain names or other identifying information.

8.7.3. Visit 2: Screening interview and consent & Enrolment

8.7.3.1. Screening & Consent

The parents of the children will be approached for a screening interview as soon as the child is sufficiently recovered to take oral medication (i.e. after completion of the blood transfusion and the standard parenteral artesunate medication [if any is indicated]). This is typically within 24 to 48 hours following admission to the hospital. During this interview, consent will be sought from the parents or guardian and if consent is granted, the eligibility criteria for inclusion into the study will be assessed (for the criteria, see section 8.4.2, page 26). For further details regarding the consent procedure see also section on consent procedure, section11.4, page 57.

8.7.3.2. Screening log

The investigator will keep a subject screening log for all subjects considered for enrolment regardless of whether they were enrolled, which, combined with the pre-screening log will be used to establish that the study sample was selected without bias. This screening log will not contain names or other identifying information.

8.7.3.3. Assignment of study IDs

Screened subjects who meet all eligibility criteria will be issued a study subject number during this visit. Once issued the study subject number they will be considered as 'enrolled.' This number is the subject's unique identifier and used to identify the subject on the CRFs. Subject numbers will be assigned strictly sequentially as subjects enter the study. Once a number has been assigned no attempt will be made to use that number again, for example if a subject discontinues or is a screening failure.

8.7.3.4. Clinical assessment:

After consent is obtained and the subject's eligibility is confirmed, the subject's demographic data will be recorded in the CRF, and all relevant clinical information, including the previous and current medical history, and laboratory information copied from the hospital's clinic and laboratory notes to the CRF (see CRF for details). A further clinical examination (including anthropometrics) will be performed and a medical history taken that will serve as the baseline examination and captured on the CRF.

8.7.3.5. Baseline Laboratory Measurements:

As part of standard of care: Children with severe anaemia are expected to have the following tests done as standard of care before blood transfusion: Malaria smear or RDT, haemoglobin concentration, blood smear to type the anaemia, blood cultures (if patient is febrile and facilities are available), complete blood count haemogram (subject to availability of automated analysers). In addition they are expected to have a blood group and cross match test with donor blood as part of the blood transfusion process. After completion of the blood transfusion, a post transfusion haemoglobin concentration is taken. If the patient was suspected to have malaria, a repeat malaria smear or RDT is often taken to evaluate whether the parasitemia has been cleared.

We shall record all these laboratories results and any others tests results such as urinalysis and radiological results that might have been done as part of standard of care. During the pre-study screening period the study team shall support the hospital to facilitate the routine use of these tests

and the recording of clinical and laboratory information on the clinical and laboratory forms and hospital registers to ensure they are available to the study team once the patient is enrolled into the study.

Study specific samples: In addition to the standard of care tests, a venous blood sample will be taken (3 ml) through the existing cannula where possible (in which case no additional 'prick' will be required). This will be used for red cell morphology, and human and parasite genetics including pheno/genotyping for polymorphisms selected for malaria or other causes of severe anaemia. The pheno/genotypes of primary interest are those for hemoglobinopathies (sickle cell disease and thalassemia) and G6PD deficiency. Blood will also be stored for genotyping of the malaria parasite (and again at initiation of PMC [visit #4, section 8.7.5, page 39] and during unscheduled sick child clinic visits for clinical malaria episodes, and the final post-PMC [visit #7, section 8.7.7, page 40]).

HIV testing or collection of information on HIV-exposure (i.e. maternal HIV status) will not be done as a study specific procedures, but the information will be copied from the clinical and laboratory hospital records of patients, including information on the maternal HIV status, where available, or the HIV-status will be obtained following provider initiated testing and counselling (PITC) procedures that are part of routine in-hospital care. Where required the study will support the hospital infrastructure needed to generate this information for the study to ensure this information is available for all study participants.

8.7.3.6. Location of household and issuance of study ID card

A detailed route description to locate the participant's household, including sketching a map, if needed, will be recorded, to aid the planned PMC treatment follow up visits at home. Lastly, a subject's identification card will be issued.

8.7.4. Visit 3: AL treatment in hospital/discharge

The first study dose(s) of the first course of AL drugs will be administered to the child whilst in hospital. These will be directly supervised by study staff. Any AL doses provided as part of routine in-patient care by hospital staff prior to study enrolment will also be recorded to ensure that children do not receive more than 6 cumulative doses of AL over 3 days. Some questions about patient's costs will also be asked during this visit (see section 9.8, 'Economic Evaluation sub-Study', page 55)

8.7.5. Visit 4: Randomisation and 1st PMC treatment visit

Randomisation will take place during visit 4, 2 weeks post enrolment, by opening a single concealed envelope containing the study drugs which will be pre-packed by the study pharmacist (for full details about the randomisation and blinding, see section8.10, 'Assignment of interventions and blinding', page 42. The first PMC treatment visit can take place at home or in the clinic, subject to local circumstances. The first dose of medication will be observed, and the family will be given the study drug to administer at home on the subsequent 2 days. Prior to drug administration, a finger prick will be taken for Hb determination and a filter paper blood spot for parasite genetics.

8.7.6. Visits 5 and 6: 2nd and 3rd PMC treatment visits

The 2nd and 3rd PMC treatment will be given 6 and 10 weeks following enrolment. These treatment visits will take place at home by a home visitor who will administer the first day of PMC study drugs, and will give the family the rest of the dose to be administered at home. If children are found to be

ill during these home-visits they will be referred to the study clinic for further evaluations as part of the unscheduled visits described below (section 8.7.8, page 40). The presence and use of insecticide treated nets will be recorded during these home visits.

8.7.7. Visit 7: 6-month scheduled follow-up

Study participants will be asked to return to the study clinic at the hospital at 6 months. Phone reminders or home visits reminders will take place prior to this visit to enhance the uptake assessment. At this visit a history will be taken and examination performed. A blood sample will be taken for malaria slides, parasite genetics and full blood count. Additional treatment for malaria and / or anaemia will be given if required. Study staff will visit the house if the family fails to present for this visit at the clinic.

8.7.8. Unscheduled visits (passive follow-up)

8.7.8.1. Intercurrent illness

A passive surveillance system will be used to monitor intercurrent illnesses through study-clinics. Parents will be instructed to bring their child to the study clinic for any suspected illness. The ‘chief complaint’ (reason for attending the clinic during passive surveillance) and ‘diagnosis’ will be recorded using a standard list based on the International Classification of Disease (ICD-10) for children.[35] Blood samples for malaria smears, parasite genetics (filter paper dried blood spots) and haemoglobin will be taken if clinically indicated (e.g. documented fever ≥ 37.5 °C axillary or >38.0 °C rectal, or a history of fever in the last 24 hours).

8.7.8.2. Verbal autopsy visit

All parents of children who die will be visited at home as soon as possible for a detailed verbal autopsy interview using standardized questionnaires that will be used to categorize the potential cause of death as probable, possibly, or not malaria related.

8.8. SAMPLE SIZE

8.8.1. Trial

8.8.1.1. Sample size re-estimation

Following recommendations from the DMEC and TSC, a blinded interim sample size re-estimation was conducted to take the lower than expected rate of loss to follow-up into account and the higher than expected pooled event rate of the composite primary endpoint (death or all-cause readmission) across both arms. This was favoured over an interim analysis, because the available funding did not allow and extension of the recruitment period, even if the results of any interim analysis would suggest this was required.

The revised sample size calculations were conducted in PASS (v15) software using a tests for the ratio of two Poisson rates. A total sample size of 1040 children (520 per arm) is required to detect a 25% reduction in the incidence of the composite primary outcome from 1,152 per 1000 child years (530 events per 1000 children during the 24 weeks from randomization at 2 weeks to the end of follow-up at 26 weeks) in the control arm to 864 per 1000 child years (398 per 1000 children over 24 weeks) in the intervention arm (power 80%, α=0.05), allowing for 10% loss to follow-up. The same

sample size would also provide 90% power to detect a 28.7% reduction in the primary endpoint from 1,152 to 822 events per 1000 children years.

8.8.1.2. Original sample size estimation

Sample size calculations were conducted using EAST (v6.2) Software. The total sample size will be 2212 children (1106 per arm) across both countries pooled. A sample size of 1858 is required to allow detection of a 30% reduction in the mean incidence rate (incidence density) of the composite primary outcome (death or all-cause readmission) from 469 per 1000 child years(216 events per 1000 children during the 24 weeks from randomization at 2 weeks to the end of follow-up at 26 weeks) in the control arm to 328 per 1000 child years (151 per 1000 children over 24 weeks) in the intervention arm (power 90%, α=0.05).A single child can have multiple (repeated) primary events. 1865 children are required to allow for 1 interim analysis using the Lan-DeMets spending function with O'Brien-Fleming type boundaries to preserve the overall two-sided type I error rate for efficacy (see.9.5.1.2, page 49). To allow for 15.7% loss to follow-up a total of 2212 children will be conducted.

The impact estimate of 30% is based on the previous trial in Malawi where the protective efficacy (PE) with monthly AL was 30% (and 31% for death or admission for malaria or anaemia). The incidence rate of 469 per 1000 child years is the average incidence rate observed during the 2-26 weeks post-discharge period in the control arm in the previous trial in Malawi[7] (536 per 1000 child years) and the rate in western Kenya in 2013 (402 per 1000 child years). The estimated rate of loss to follow of 15.7%is conservative and three times the rate observed in our previous studies that involved 6 months follow-up of children admitted for severe malaria or severe anaemia: this was 7.4% in the earlier IPTpd/PMC trial in Malawi across 4 hospitals,[36] and 3.5% and 5.0% in two previous observational studies in Uganda.[37,38] However the study will involve a total of 7 hospitals, only 3 of which are experienced research hospitals. We have therefore allowed for 15.7% loss to follow-up, which is the average of 10% in the 3 experienced sites and 20% in the remaining 4 sites.

8.8.2. Cardiac monitoring study

A sample size of 26 children is required to achieve 90% power to detect a difference of 20.0 ms in QTc time between the mean QTc observed 4 to 6 hours after the 3rd dose of the first 3-day course of DP at 2 weeks and the last dose of the last 3-day course of DP at 10 weeks, assuming an estimated standard deviation of 31.0 (two-side alpha 0.05). The SD values are based on observations from previous studies in Uganda with monthly IPT with DP in children 6 to 24 month of age.[27,34]To account for 20% loss to follow-up or missed ECGs, 33 children will be recruited. Because the study is placebo controlled 33 children will be recruited per arm; i.e. 66 in total.

8.9. RECRUITMENT STRATEGIES FOR ACHIEVING TARGET SAMPLE SIZE

The enrolment of the target sample size is scheduled to be completed in a 24-month period, requiring an average of 316 children per site (for 7 hospitals), or 158 per site per year. The study sites in each country will be chosen based on their potential to recruit at least 200 children, based on the number of cases receiving blood transfusion within the given age range. The presence of other studies that could potentially restrict the number eligible children will also be taken into consideration. Recruitment will be competitive between sites in each country to enhance the

chances of completion within 2 years. Recruitment will start in approximately 7 hospitals and can be expanded to approximately 10 hospitals if low recruitment rates are encountered. We have therefore included additional backup hospitals (see 8.3, 'Study settings', page 23).

8.10. ASSIGNMENT OF INTERVENTIONS AND BLINDING

8.10.1. Allocation

8.10.1.1. Allocation sequence generation

Eligible children will be randomly assigned to either PMC or placebo group with a 1:1 allocation as per a computer generated randomisation schedule stratified by age group and site using permuted blocks of random sizes. The length of each block will be randomised and will not be disclosed to ensure blinding is fully maintained.

The randomisation schedule will be drawn-up in advance by the study statistician. Recruitment will be 'competitive' between the sites within a country (i.e. the number of subjects recruited at each site may differ depending on the respective rates of admission). To allow for this, 600 individual randomisation envelopes will be prepared for each site to ensure equal distribution across study arms within each site.

8.10.1.2. Allocation concealment mechanism

The active PMC and placebo drugs for each participant will be pre-packed in opaque sealed envelopes containing 3 other envelopes (one for each PMC course) or a sealed box with three bottles. The envelopes or bottles containing active DP or placebo will look identical, and the appearance and consistency of the tablets will also be identical. Each envelop or box will be prepared with the sequential numbers, but not yet with the study subject number supplied. The preparation and packing of the study drugs will be performed by a dedicated pharmacist assistant who will remain unblinded during the study in order to package the blinded subject-specific study treatment, in accordance with the randomisation scheme. He/she will not be involved in any other component of the study. Because the master code and individual codes are kept in sealed envelopes in a central location off site (see section 8.10.2), neither the caretakers, study staff, nor investigators will be able to know the next study group assignment in the allocation sequence.

8.10.1.3. Allocation implementation

The allocation will occur at 2 weeks post-discharge, during the first PMC treatment visit, just prior to the first dose of PMC. On the day of randomization for each child, the child's study identification number will be recorded against each envelope/box number by writing the study ID on the envelope or box prior to it being opened. Once the participant's study ID has been written on the envelope/box, the child will be considered randomized.

No subject will be randomised into the study more than once. If a subject number is allocated incorrectly, the subject number should not be reassigned and randomisation should continue with the next sequential number. The sponsor should be informed immediately and the investigator will be instructed on other appropriate procedures to be followed.

All enrolled subjects who subsequently do not meet eligibility requirements for randomisation at visit 4, 2 weeks later, will be considered screening failures and will not be randomized, with the exception of children that fulfil the criteria to delay randomisation as described in section 8.4.3, 'Eligibility criteria for randomisation into study (at 2 weeks)', page 27.

8.10.2. Blinding

Neither the caretakers, study staff, nor investigators will know which arm a child is in. Thus the treatment group may only be determined by comparing the child's study id to the blinded list of envelope/box numbers. The master code to randomisation code will be kept in a sealed envelope. Only the study pharmacist (on site), and the trial statistician and the DMEC statistician (both off-site) will have access to master-code until the study is completed or stopping rules are reached and unblinding is required. The randomisation scheme will also be available to the principal site investigator and the study safety monitor, but only as individual treatment code envelopes, indicating the treatment for each randomised subject. The international collaborators, clinical monitors, or other personnel from any Contract Research Organization (CRO) handling the data on behalf of the sponsor, will have no access to the randomisation scheme. The treatment allocations will be disclosed after the data is locked and a statistical analysis plan has been submitted to the DMEC.

All laboratory tests will be conducted by subject study number, date of birth and date of sample. Laboratory personnel will be unaware of the randomisation group of the subject. The study statistician conducting the interim analysis will remain blinded throughout the analysis.

8.10.2.1. Emergency unblinding

The individual treatment code must not be broken by the investigator, except in medical emergencies necessitating the immediate identification of the treatment randomisation for the appropriate management of subject. The date, reason and name of person breaking the code must be documented and reported to the sponsor. If so clinically indicated, the subject will be withdrawn from receiving further study drug. Because the code is kept in individual envelopes, the treatment code can be maintained for the other subjects.

8.10.2.2. Unblinding at the end of study

Before the treatment code is broken for statistical analysis, the code for each participant will be returned to the sponsor with a documented explanation for each episode where the code was broken. Any master code supplied (e.g. to the pharmacy) will be returned to the sponsor. No copies of the code will be taken by any of the investigators involved in the field work of the study. When the validation and editing process is concluded the formal 'locking' of the database will be documented. Data for each individual participant will be classified and coded with respect to its inclusion in the various statistical analyses planned in the study and the code entered into the data base. After the above actions have been documented the treatment code can be broken and included in the data base for each individual participant. Copies of the treatment code will be available to the investigator at the end of the study after the database is 'locked'.

9. DATA COLLECTION, MANAGEMENT AND ANALYSIS

9.1. DATA COLLECTION METHODS

Data will be collected and recorded at the point of contact; i.e. the health facility or at the study participants home by one of the trained study staff. Site supervisors will check data collection forms at the end of each day for completeness and accuracy of recording.

The study will use electronic care records forms to be entered using laptops, computer tablets and smartphones, and/or paper-based data collection forms and optical character recognition (OCR) software (e.g. HP TeleForm) for creating and scanning the forms. Completed clinical record forms and relevant source documents will be scanned on site or in a central location in each country and images will be transferred (encrypted) to a central server where they will be processed into databases via OCR.

9.1.1. Data management

Original data collection forms will be handled only by study staff and kept under locked storage until completely coded, checked and transported for data entry. Once data entry and cleaning are complete any hard copies of CRFs will be stored for at least 3 years at secure storage facilities in Kenya and Uganda as per local storage policies and guidelines. After that CRFs will be shredded and copies of source data will be kept electronically in compliance with prevailing laws on data storage. If the study site is located in an area where KEMRI-CDC, Makerere University or other research groups are collecting demographic and health surveillance data will be linked using he unique identifiers for the individual or for the household of the individual to obtain household level demographic, educational and socio-economic data. After completion of the study, country specific data will be stored in each country, thus Kenyan data will be stored at KEMRI and the Ugandan specific data will be store at Makerere University College of Health Sciences. The pooled data will be stored by the sponsor of the study, the Liverpool School of Tropical Medicine (LSTM), UK and the Norwegian Social Science Data Services (NSD, http://www.nsd.uib.no/nsd/english/index.html), as per requirements of the Research Council of Norway, who fund this trial. The electronic data will be kept for at least 15 years.

9.2. STATISTICAL METHODS

A detailed study statistical analytical plan for the final analysis, that will supersede the study protocol, will be drawn up during the course of the study before the unblinding of data at database lock.

9.2.1.Trial profile and flowchart

A trial profile will be developed and presented as a flow chart following CONSORT guidelines, consisting of the number of participants screened, eligible, enrolled, randomized, and followed to 6 months post-discharge, number contributing to primary efficacy outcomes. It will also include the number of participants who withdrew or were lost to follow-up.

9.2.2.Baseline characteristics

Descriptive statistics of baseline characteristics, overall and by treatment group will be provided in a table consisting of parameters collected prior to randomisation. No statistical comparisons

will be made between the groups, but any differences between groups at baseline which are also associated with the outcome variable will be taken into account in subsequent analysis.

9.2.3. Analysis Populations

9.2.3.1. Screening failures

If a subject gives informed consent and is provided with a study ID, but then is lost from follow up, dies or withdraws before randomisation at 2 weeks from enrolment or does not fulfil the randomisation eligibility criteria, they will be classified as a randomisation screening failure and excluded from the ITT and the ATP analysis, but they contribute to a separate analysis of the risk and determinants of the different outcomes in the first 2 weeks since enrolment prior to randomisation.

9.2.3.2. Intention to treat population

The Intention-to-treat population is defined as all randomized subjects with a valid informed consent.

9.2.3.3. Per protocol population

The per-protocol population is a subset of the ITT population. Subjects with major protocol deviations will be excluded from PP population. Major protocol deviations will be defined in the Statistical Analysis Plan (SAP)

9.2.3.4. Safety population

All children who were randomized, received the first dose of study intervention and were followed up; i.e. provided information on potential adverse events.

9.2.4. Missing Data

Every effort will be made to minimise the amount of missing data in the trial. Whenever possible, information on the reason for missing data will be obtained. No adjustments will be made for missing outcome data, but missing data may be imputed for co-variates.

9.2.5. Assessment of efficacy

9.2.5.1. Primary analysis

Primary analysis will be by intention to treat, and include all primary endpoint events to capture the potential effect of PMC on reducing first and repeat events. The associated statistical null hypothesis is that there is no difference between the treatment groups in the distribution of the incidence rate of the primary endpoint, and the alternative hypothesis is that there is a difference between treatment groups.

The follow-up time will be measured as the time in days from the date of randomisation to the end of follow-up, death or drop-out. The incidence rate will be calculated per arm and the rate ratio (RR, PMC to placebo) and 95% confidence intervals (CI) estimated using Poisson regression models, stratified by country, with treatment (as randomized) as the only co-variate. The results will be expressed as the relative rate reduction (RRR) (95% CI) calculated as 100 multiplied by (1–RR).

In addition to the final analysis, the primary statistical hypothesis will be tested in an interim analysis when approximately half of patients are recruited as described in more detail in section 9.5.1.2, page 49.

9.2.5.2. Subgroup analysis

We will use stratified analysis to assess to what extent the effect of the intervention on the primary outcome is influenced by the following potential effect modifiers:

Demographic modifiers

1. Age: Infants vs older children. PMC is hypothesized to be more effective in older children based on results from the previous trial in Malawi.[7]
2. Socio-economic and/or educational status: Quintiles based on SES-index rank score. PMC is hypothesized to be more effective in children from poorer and less educated households as they may have more, and more severe post-discharge events because of barriers resulting in delays in seeking appropriate care.
3. Distance to nearest study clinic: terciles. Distance from health facilities has been associated with increased mortality, and can interfere with event capture.

Clinical modifiers

4. Syndrome on admission: non-malaria severe anaemia vs severe malaria anaemia. PMC is hypothesized to be more effective in children with severe malaria anaemia on enrolment as they represent a selected subgroup that is more exposed to malaria for environmental, behavioural or host-genetic reasons.
5. HIV status: HIV-infected vs HIV-uninfected. PMC is hypothesized to be more effective in HIV uninfected children as HIV-infected children may have high rates of readmissions due to other causes and may benefit from antimalarial prophylactic properties of daily cotrimoxazole.
6. Hb on randomization: Terciles. PMC is hypothesized to be more effective in children with the lowest Hb as they may be at highest risk of rebound severe anaemia without an intervention.
7. Previous hospital admittance: lowest 50% vs highest 50%. PMC is hypothesized to be more effective in children with a history of previous admissions as they represent a selected subgroup at highest risk of severe anaemia or malaria for environmental, behavioural or host-genetic reasons.

Transmission variables

8. Malaria transmission intensity: moderate vs high. PMC is hypothesized to be more effective in children living in high transmission areas as the relative contribution of malaria to post-discharge morbidity may be higher and thus more malaria related events can be prevented.
9. Residence: Urban vs rural. PMC is hypothesized to be more effective in children living in rural areas which have higher malaria transmission.
10. Season: Terciles, based on average rainfall during the 6 month study period for each child. PMC is hypothesized to be more effective in malaria transmission season when more potential malaria related events can be prevented.
11. ITN use vs non-use. PMC is hypothesized to be more effective in non ITN users as more potential malaria related events can be prevented.

12. Study Site: about 7 sites, as used in the stratification. PMC is hypothesized to be more effective in children living in high transmission sites as the relative contribution of malaria to post-discharge morbidity may be higher and thus more malaria related events can be prevented.

Intervention modifiers and time of assessment

13. Period of assessment: extended follow-up period 15-26 weeks vs PMC period 2-14 weeks. PMC is hypothesized to be much more effective during the PMC period when drug levels remain above the minimum inhibitory concentrations.
14. Dose in mg/kg received: Terciles. It is hypothesized that variations in dose received in mg/kg due to natural variation in bodyweight within the fixed weight bands, are small and will not affect efficacy.

Because the study is not designed to have sufficient power for subgroup analysis, we will interpret the results of subgroup analysis cautiously. No adjustment will be made for multiple comparisons.

9.2.5.3. Sensitivity analysis

A number of sensitivity analyses will be conducted to assess the robustness of the primary endpoint analysis. These include un-stratified Poisson regression models, analysis of the per-protocol subject population, and covariate adjusted analysis. Other regression methods including negative binomial regression and models for recurrent event time data will also be explored. Additional post-hoc analyses may also be conducted if deemed necessary. In addition, the results of the statistical models with and without imputation for missing values for co-variates values will be compared. Covariates for co-variate adjusted analysis and subgroup analysis, and alternative statistical models, which will be specified in the statistical analysis plan (SAP).

9.2.6. Analysis of adverse events

Adverse reactions will be reported and tabulated for each treatment arm, overall and per body system and adverse event, on an intention to treat basis. Treatment emergent adverse events are defined as adverse events that had an onset day on or after the day of the first dose of study medication. Adverse events that have missing onset dates will be considered to be treatment emergent. No formal statistical testing will be undertaken. All laboratory data will be listed.

9.3. PROCEDURES FOR ASSESSING EFFICACY PARAMETERS

9.3.1. Primary outcome

All-cause mortality will be assessed during the visits at 2, 6, and 10 weeks to administer PMC and the end of study visit at 6 months.

All-cause and disease specific re-admissions will be assessed through passive case detection and through a questionnaire administered during the visits at 2, 6, 10 weeks and 6 months. Details of the admission and treatment provided will be recorded, where available, on special study forms including RDT or malaria smear results and use of antimalarials, to allow for differentiation between malaria, severe anaemia, and other syndromes.

9.3.2. Secondary outcomes

All-cause and malaria specific clinic visits will be assessed through passive case detection and through questionnaires administered during the visits at 2, 6, 10 weeks and 6 months. In order to facilitate passive surveillance, all participants will be provided with unique study ID card for identification of study participants during unscheduled visits to outpatient departments or hospitals. Caretakers will be encouraged to seek care from the study clinics. Details of clinic visits will be recorded on special study forms including RDT or malaria smear results to allow for differentiation between malaria and non-malaria clinic visits.

9.4. LABORATORY METHODS

9.4.1. Techniques for host and parasite genetic assays

Sample aliquots or DNA aliquots will be sent to the laboratories of Dr Bailey, University of Massachusetts, and Dr Juliano, University of North Carolina, allowing for efficient well-controlled and cost-effective genetic analysis. We will be using deep sequencing techniques using massively parallel sequencing with which both Dr Bailey's and Dr Juliano's laboratories have extensive experience in terms of all laboratory and computational aspects [40,41,42]. High-throughput sequencing allows for the rapid analysis of multiple loci and multiple samples. Molecular Inversion Probe techniques [43,44]will be used that will allow the testing of multiple loci for each participant within a single tube and batch analysis of hundreds of participants within a single sequencing run [40,41,42]. A strength of molecular inversion probes is that it affords single tube testing per sample for a panel of genes and allows for the rapid addition of additional genes as they are discovered, which is important given the ongoing concern about artemisinin resistance in Asia and Africa. A comprehensive panel for all known drug resistance loci in *P. Falciparum* developed in the laboratory of Dr Bailey (unpublished) will be used for the parasite genetics.

Because the high-throughput sequencing techniques are most amenable to being able to batch hundreds of samples (if not thousands) at a time, we expect to perform the majority of the analysis towards the end of the field work when all or the majority of the samples have been collected in order to provide cost-effective and well controlled analysis.

9.5. MONITORING

9.5.1. Data Monitoring

9.5.1.1. Data Monitoring and Ethics Committee (DMEC)

Since the study is a clinical trial in a potentially vulnerable patient population, an independent Data Monitoring and Ethics Committee (DMEC) will be set up. The DMEC will be critical to ensure that the subjects are protected from harm, while also ensuring that the study integrity is not compromised. The DMEC will consist of 3 or 4 independent members knowledgeable in the conduct of clinical trials. They will meet regularly (e.g. twice yearly or more frequent if so required) during data collection period to provide a review of blinded (and if requested unblinded) data to ensure the safety, rights and well-being of trial participants. The trial statistician could also be asked to attend the meetings. The role and membership of the DMEC is described in more detail in Appendix III. Terms of Reference Oversight committees, page 113.

9.5.1.2. Interim analyses and criteria for termination of the trial

An interim analysis will be conducted on the primary endpoint when 50% of participants have completed the 6 months follow-up. The interim-analysis will be performed by an independent statistician, blinded for the treatment allocation. The statistician will report to the independent DMEC. The DMEC will have unblinded access to all data and will discuss or report the results of the interim-analysis with the Trial Steering Committee (TSC), e.g. in a joint meeting. The TSC decides on the continuation of the trial and will report to the ethics committees.

The Lan-DeMets spending function with O'Brien-Fleming type boundaries will be employed and the sample size inflated (see 8.8.1, page 40) to preserve the overall one-sided type I error rate for efficacy at the α=0.05 level at the final analysis. If the stopping boundary is crossed at the interim analysis and the RR is less than 1, i.e. the observed incidence rate in the PMC arm is less than the expected incidence rate under the null hypothesis, it will be concluded that the study has demonstrated that the efficacy of PMC is superior to that of placebo in the prevention of death or all-cause readmission. The trial recruitment can then be stopped unless the DMEC advises otherwise. Statistics will not be the sole basis for the decision to stop or continue and the DMEC can advise to continue recruiting in the trial, or stop recruiting but continue to complete the intervention as per randomization in the remaining active children, even if statistically the stopping boundary is crossed, e.g. in order to continue collecting more safety information or data for further sub-group analyses etc. The trial will not be stopped in case of futility, unless the DMEC during the course of safety monitoring advises otherwise.

A detailed plan for interim analysis, the provisional stopping rules and how the stopping rules will be applied, will be drawn up prior to the start of the interim analysis and documented in the study statistical analysis plan.

In addition, regular review of the quality of the study data will be conducted at each meeting of the DMEC.

The sponsor reserves the right to temporarily suspend or prematurely discontinue this study at any time for reasons including, but not limited to, safety or ethical issues or severe non-compliance. If the sponsor determines such action is needed, it will discuss this with the investigator. When feasible, the sponsor will provide advance notification to the investigator of the impending action prior to it taking effect. The sponsor will promptly inform the IEC/IRB and provide the reason for the suspension or termination.

9.6. SAFETY MONITORING AND REPORTING

9.6.1. Cardiac Monitoring

To assess the impact of monthly dosing with DP on QTc prolongation, a sub-group of 66 children (33 in each arm) will be selected to participate in cardiac monitoring sub-study at the beginning of the trial using convenience sampling. Separate written informed consent will be sought for inclusion in this sub-study.

A baseline ECGs will be done prior to the start of the first dose of the first course of DP at 2 weeks post-discharge, and again 2 days later, 4-6 hours after the 3rd dose of DP, and before each

subsequent first dose and after each subsequent 3rd dose of each next course (i.e. about 6 ECGs assessments in total per child). Each ECG will be taken in triplicate. Information on food intake and co-medication will be collected by questionnaire. A 200 micro litre blood sample will be taken for piperaquine drug levels at the same time that the ECGs are taken. Piperaquine levels will be measures in a specialised laboratory from the Mahidol Oxford Tropical Medicine Research Unit (MORU), in Bangkok, Thailand which is one of the few laboratories globally that have this capacity.

ECGs will be read on site by trained local staff, and forwarded electronically to Makerere University and KEMRI Kisumu for quality control. All abnormal ECGs (see grading below) and a sub-set of other ECGs will be read by a Paediatric Cardiologist fellow. QT intervals will be adjusted for heart rate prior to subsequent analysis using the Fridericia formula (QTc=QT times the cube-root of the RR interval on the ECG).

All ECGs will be graded based on the QTc interval as mild, moderate, severe and potentially life threatening as per international guidelines from the National Institute of Health (NIH): Division of AIDS (DAIDS) Table for Grading the Severity of Adult and Pediatric Adverse Events.[45]

- Grade 1 (mild): Asymptomatic, QTc interval 450 –470 ms
- Grade 2 (moderate): Asymptomatic, QTc interval 470-500 ms
- Grade 3 (severe): Asymptomatic, QTc interval >500 ms OR ≥60 ms above baseline (pre-course level)
- Grade 4 (potentially life threatening): Life-threatening consequences, e.g. Torsade de pointes or other associated serious ventricular dysrhythmias

For all children with QTC ≥450 ms a repeat ECG will be performed within 30 minutes. Children with confirmed baseline QTC ≥450 ms will be excluded from the main trial. If QTC value ≥450 ms are found during follow-up ECGs, the ECG will also be repeated within 30 min. If findings are confirmed the next dose of DP or placebo will be withheld and the local study medical officer notified. In all cases with a confirmed grade 2-4 ECG abnormality, the next course of DP will also continue to be withheld. The decision to stop or continue the DP/Placebo in these children will be made in consultation with the DMEC. This will be reported as an SAE to the ethics committees.

At completion of the Cardiac monitoring study an analysis will be performed by an independent statistician, blinded for the treatment allocation. The statistician will report to the independent DMEC. The DMEC will have a meeting during which they will have access to the unblinded data. The following approach will be taken in terms of stopping rules:

- If there is no evidence of a clinically relevant increase in QTc prolongation when comparing the QTc prolongation observed after the 3rd dose of the first course (relative to baseline), and after the 3rd dose of the 3 course (as per the previous trial in children 6 to 24 of age in Uganda),[27,34] they will inform the Trial Steering Committee (TSC) for the study to continue as per the approved protocol. The ethics committees will be informed.
- If there is evidence that the QTc prolongation increases with each course, the DMEC will share the results of the interim-analysis with the Trial Steering Committee (TSC) in a joint meeting. The TSC decides on the continuation of the trial and if needed can suggests amendments to the protocol, including screening for QTc prolongation in all remaining children in the trial, and/or recommend alternative strategies for PMC, e.g. 6-weekly instead

of monthly DP, or use of alternative drugs. The results and any amendments to the protocol will be submitted to the ethics committees for approval.

9.6.2. Definitions

The principles of ICH GCP require that both investigators and sponsors follow specific procedures when notifying and reporting adverse events or reactions in clinical trials.

The following definitions apply to this protocol:

9.6.2.1. Adverse Event (AE)

Any untoward medical occurrence in a patient or clinical trial subject administered a medicinal product and which does not necessarily have a causal relationship with this treatment.

9.6.2.2. Adverse Reaction (AR)

Any untoward and unintended response to an investigational medicinal product related to any dose administered.

Comment: All adverse events judged by either the reporting investigator or the sponsor as having a reasonable causal relationship to a medicinal product would qualify as adverse reactions. The expression 'reasonable causal relationship' means to convey, in general, that there is evidence or argument to suggest a causal relationship.

9.6.2.3. Serious Adverse Event (SAE) or Serious Adverse Reaction (SAR)

Any adverse event or adverse reaction that results in death, is life-threatening*, requires hospitalisation or prolongation of existing hospitalisation, results in persistent or significant disability or incapacity, or is a congenital anomaly or birth defect.

Comment: Medical judgement should be exercised in deciding whether an adverse event/reaction should be classified as serious in other situations. Important adverse events/reactions that are not immediately life-threatening or do not result in death or hospitalisation, but may jeopardise the subject or may require intervention to prevent one of the other outcomes listed in the definition above, should also be considered serious.

*Life-threatening in the definition of a serious adverse event or serious adverse reaction refers to an event in which the subject was at risk of death at the time of the event. It does not refer to an event which hypothetically might have caused death if it were more severe.

9.6.2.4. Suspected Unexpected Serious Adverse Reaction (SUSAR)

An adverse reaction that is both unexpected (not consistent with the applicable product information) and also meets the definition of a Serious Adverse Event/Reaction.

9.6.3. Reporting adverse event procedures

All SAEs will be reported to the in country principal investigator or an assigned representative within 24 hours of the staff becoming aware of it, using an SAE form, which should be completed, scanned and sent electronically. The SAE form asks for nature of event, date of onset, severity, corrective therapies given, outcome and causality (i.e. unrelated, unlikely, possible, probably, definitely). The responsible study clinician should assign the causality of the event.

9.6.3.1. Expedited reporting

SAEs that are unexpected and are at least ‘possibly related’ to the study drug require expedited reporting within 24 hours of the country principal investigator or assigned representative becoming aware of it (e-mail notification); i.e. this will be a maximum of 48 hours after the event occurred (including the 24 hours required for the field staff to report to the principal investigator / representative). Additional information will be sent within 14 additional days (full SAE report) if the reaction had not resolved at the time of e-mail notification.

9.6.3.2. Annual reporting

Other SAEs and AEs will be reported annually in an aggregated report. AEs that will not be reported include common childhood illnesses that do not result in hospitalization, including but not limited to clinical malaria, respiratory, gastrointestinal, and skin diseases, unless they are considered at least possibly related to the intervention.

9.6.3.3. Other reporting

In addition to the annual reported, an interim report will be prepared early on in the study shortly following the completion of the cardiac monitoring sub-study (see 9.6.1, page 49).

9.6.3.4. Recipients of reports

The study will comply with local regulations pertaining to reporting of SAEs to their local Research Ethics Committee and/or Research & regulatory offices. In addition to the primary ethics committees, we will report safety data to the DMEC, the sponsor and to Sigma Tau, the manufacturer of DP who in turn will report to the European regulator EMA. A copy of the final study report will be provided to all RECs, DMEC, local regulator, and Sigma Tau.

Figure 2: Safety reporting assessment flowchart[46]

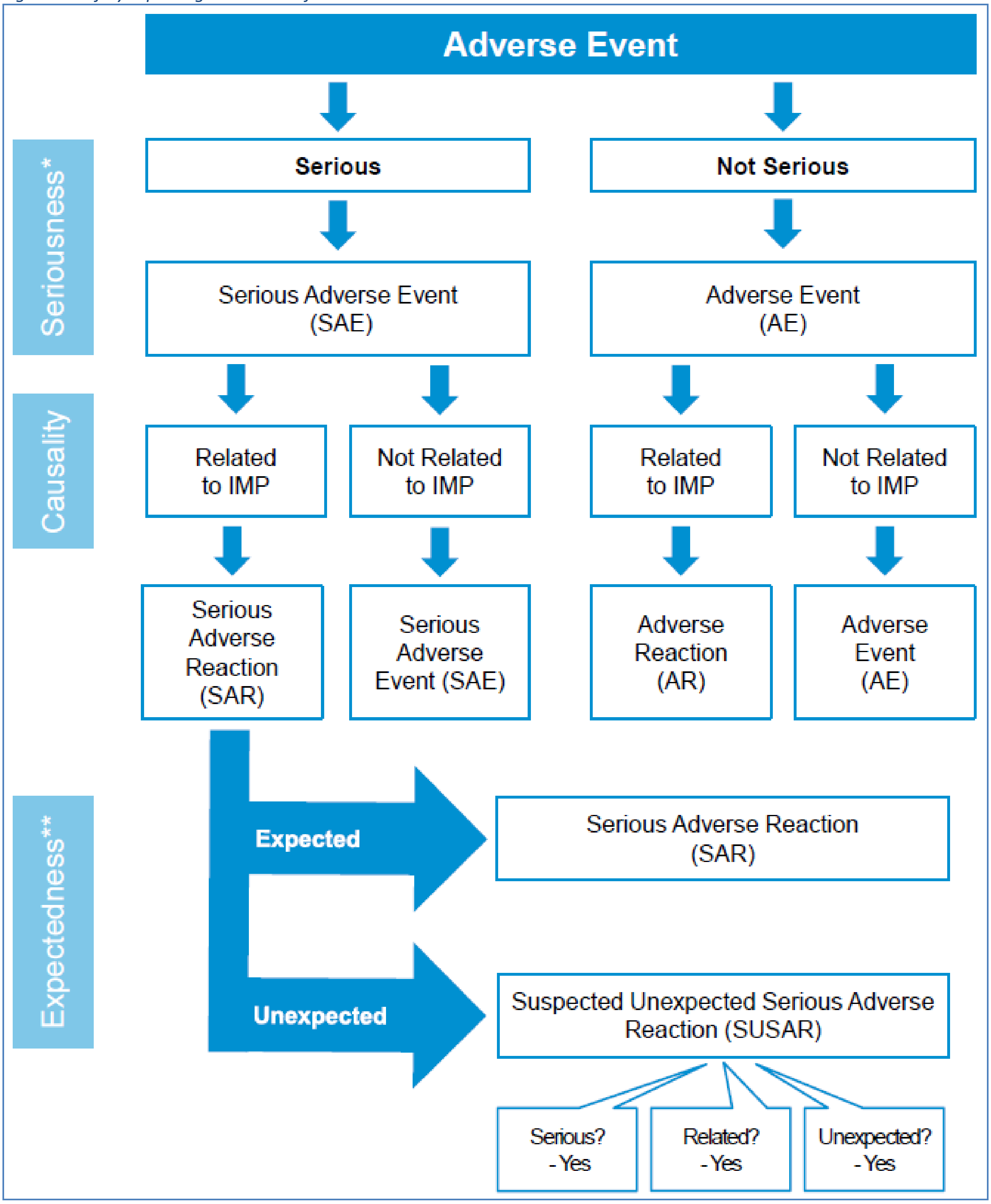

IMP: Investigational Medicinal Product
*See definition of SAE in section 9.6.2.3, page 51
**Assessed in line with the current approved Investigator's Brochure (IB)

9.7. QUALITY ASSURANCE

9.7.1. Clinical monitoring and auditing

9.7.1.1. Clinical monitoring

Monitoring of this trial will be conducted to ensure compliance with Good Clinical Practice and scientific integrity will be managed and oversight retained, by the sponsor. Clinical monitoring will be sub-contracted to an independent clinical monitor; at least 3 visits are planned including a study initiation visit in the first year, and then half-way and at trial close out.

Prior to subject enrolment, the monitor will visit the study site to determine the adequacy of facilities, review the protocol and data collection procedures and discuss the responsibilities of the investigator and other study site personnel.

During the study, the monitor will have regular site contacts, including conducting on-site visits to:

1. Confirm that the study is being performed according to the protocol, ICH GCP and applicable regulations, data are being accurately recorded in the CRFs and that investigational product accountability is being performed.
2. Conduct source data verification
3. Confirm facilities remain acceptable
4. Provide information and support to the investigators
5. Evaluate study progress

Upon completion of the study the monitor will visit the study site to verify that all CRFs are completed and collected, all data queries have been resolved and filed, conduct final accountability, reconciliation and arrangements for investigational product and verify all study site records are complete.

The PI and relevant staff will be available at monitoring visits and agree to allocate sufficient time to the monitor to discuss any issues and address their resolution.

9.7.1.2. Auditing

The independent clinical monitoring process will be audited by a study staff from the sponsor's research office at LSTM in Liverpool, UK. The auditor will accompany the clinical monitor during at least one of the site visits. After this visit it will be determined by the sponsor if more auditing visits are required.

9.7.2. Training

The country principal Investigators are responsible for the conduct of the study at the study sites, including delegation of specified study responsibilities, and training of study staff. Each site in Kenya and Uganda will maintain a record of all individuals involved in the study (medical, nursing and other staff) and will ensure that all persons assisting with the trial receive the appropriate training about the protocol, the investigational product(s) and their trial-related duties and functions, including formal certified GCP training. During the study the regular spot checks will be conducted to assess the performance of study site staff members and re-training provided where necessary.

9.7.3. Quality assurance/control of laboratory tests

Regular audits of laboratory performance will be completed by experienced supervisors according to standard operating procedures. All malaria blood smears will be read by two different microscopists blinded to the RDT and each other's results, any significantly discordant results based on positive/negative results or difference in parasites above a defined threshold will be verified by a third expert microscopist. All RDTs will be stored and transported at the recommended temperatures and testing for malaria parasites will be done as per the instructions of the manufacturer.

9.8. ECONOMIC EVALUATION SUB-STUDY

The economic evaluation will provide information about incremental costs, incremental health benefits and cost-effectiveness of PMC compared to the current standard of care in Kenya and Uganda. The overall aims are to inform decision makers whether PMC as implemented in this trial is likely to be cost-effective if routinely implemented.

A novel decision-model (Figure 3) will be developed to capture aspects relating both to protective efficacy and to country specific implementation characteristics including different implementation costs. Since recurrent events are common, and since previous health events are risk factors for new events, a micro-simulation model is adequate to capture costs and health outcomes. The influence of single parameters will be analysed with one-way sensitivity analyses, while overall decision uncertainties will be estimated utilising probabilistic sensitivity analysis. Incremental costs will be calculated per case averted of severe anaemic malaria, per death averted and per disability adjusted life year (DALY) averted. Treatment efficacy will be based on the results of the proposed trial in Kenya and Uganda and on the pooled results from this trial and the previous trial in Malawi[7]. DALYs will be calculated using standard assumptions from the recent Global Burden of Disease Study.

Figure 3 Micro-simulation model (M)

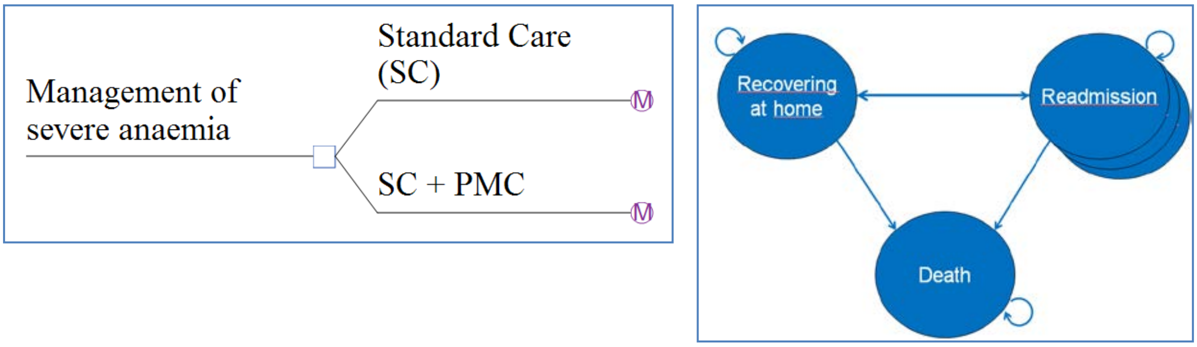

The interventions will be costed prospectively alongside the trial from the perspectives of both the health care provider and the patients/families. Costs will be calculated from a societal perspective, but results will be presented disaggregated for the health care provider and the patients/families. We will use an ingredients approach, in which resources in separate processes are identified, quantified and valued.

The economic evaluation requires collection of some data in addition to the main trial data. The families of the affected children face cost related to receiving the PMC treatment (intervention costs), and in addition they are subject to costs related to the primary disease, readmissions or

death (disease costs). Likewise, the health care providers need resources to provide the intervention, which in turn will affect future cases of disease.

9.8.1. Patient intervention costs

The patients' costs of receiving the intervention will be considered in visit #7, which is the end of study assessment (see Table 2). A number of questions will be asked to assess the additional direct and indirect costs associated with receiving the PMC intervention, including travel expenses, treatment expenses and time use for care-takers. Only costs occurring post-discharge will be considered as intervention costs. Time use will be valued assuming that national minimum wage rates represent the alternative cost of the care-takers time.

9.8.2. Patient disease costs

Patients face costs relating to treatment of the primary disease and anaemia, as well as re-admissions or death. Patients' expenses and time use related to the primary disease will be assessed during visit #3 at discharge from hospital, while their costs related to recurring cases of disease will be considered during the readmissions. Similar assessments will be done in the out-patient clinics for non-severe events occurring post-discharge. Both tradable and non-tradable cost items will be identified, measured and valued. Household economic outcomes will be estimated as in the Living Standard Measurement Surveys (worldbank.org/lsms).

9.8.3. Provider intervention costs

Provider costs of implementing PMC will utilize information from project accounts, as well as interviews with program managers and health care staff. The data collection will be done retrospectively towards the end of the clinical trial, and efforts will be made to separate intervention costs from costs related to research activities.

9.8.4. Provider disease costs

The assessment of disease treatment costs from the perspective of the health care provider will combine data from facility records and accounts, interviews with health care workers in the hospital and patient cards, and will capture direct and indirect health care provider, capital and recurrent costs. The primary disease and anaemia will be costed prospectively for at least 10% random sample of the recruited children, while re-admissions will be costed prospectively after discharge of patients for all cases.

10. TIMEFRAME AND DURATION OF THE STUDY

The total duration of the study is 4 years, including 12 months of study preparation, approximately 30 months of field work and 6 months of data analysis and report writing. The study will include 1 interim analysis of safety data when 50% of the patients have completed 26 weeks follow-up.

11. ETHICAL CONSIDERATIONS & REGULATORY APPROVALS

11.1. DECLARATION OF HELSINKI

The trial will be conducted in compliance with the principles of the Declaration of Helsinki (1996), the principles of GCP and in accordance with all applicable regulatory requirements in Uganda and Kenya.

11.2. RESEARCH ETHICS APPROVAL

11.2.1. Review process

This protocol, the informed consent document, patient information sheets will be reviewed and approved by all the appropriate Institutional Review Boards. We shall seek ethical approval from all the relevant ethical committees including: the KEMRI Ethical Review Committee, the Makerere University School of Medicine Research and Ethics Committee, as well as Uganda National Council of Science and Technology (UNCST).In addition ethical approval (or deferral to the local ethical institution) will be sought from LSTM, Liverpool, UK, the US-based Centres for Disease Control and Prevention (CDC), Atlanta, USA, and the Regional Committee for Medical and Health Research Ethics, western Norway (REK Vest), for the University of Bergen.

11.2.2. Protocol amendments

No change will be made to the approved protocol without the agreement of the sponsor.

If it is necessary for the protocol to be amended, the protocol amendment will be submitted to the IRB/IEC for approval before implementation. Any protocol amendments will be submitted to the primary ethics committees in each country before implementation. Any change to the informed consent form must also be approved by the sponsor and the primary ethics committee in each country IRB/IEC, before the revised form is used.

The sponsor will distribute amendments to each principal investigator, who in turn is responsible for the distribution of these documents to the staff at his/her study site.

11.3. REGULATORY APPROVAL AND TRIAL AUTHORISATION

Since the trial is conducted outside the EU, no authorisation from a European regulator is required. Trial authorisation will be sought from the Kenyan regulator (the Kenyan Pharmacy & Poison Board) and Ugandan National Drug Authority (NDA).

11.4. INFORMED CONSENT PROCEDURES

Written, informed consent will be obtained in the local language from the caretakers of all children that will participate in the study. Additional separate consent will be sought for research samples used for patient care vs de-linked samples used for research purposes that have no immediate clinical relevance and require short or long-term storage and shipment.

The consent process shall be initiated at the time of enrolment into the study and shall continue throughout the child's participation. Caregivers of children meeting the eligibility criteria for pre-

screening will have the study explained to them by a member of the study team.

Once the patient has stabilised and meets the study enrolment criteria, the full consent process will follow (screening phase), with a written consent form provided.

For illiterate participants, an independent witness will be present during the informed consent process and will sign the consent form as a witness. The caregivers may withdraw consent at any time throughout the course of the study, and this will be made clear in the informed consent process. A copy of the informed consent document will be given to the caregiver for their records, unless they state that they do not wish to have a copy. All individuals will be informed that there is no requirement to join the study and that standard medical care will remain the same regardless of study enrolment.

If the caregiver chooses not to have their child participate in the study, the case will be turned over to the attending physician currently on duty for routine care of their condition.

11.4.1. Consent forms

The consent form will include two parts. This first part gives permission for all study related procedures, including copying all relevant information from the hospital and clinic records and laboratory registries and the collection of biological samples. The second part will give permission for long-term storage of the blood for future studies as well as for genetic studies on the patient's blood sample related to malaria and anaemia. For this test the samples shall be stored as frozen venous blood samples and in filter papers and shipped for analysis to the respective laboratories.

11.5. PROTECTION OF PRIVACY AND CONFIDENTIALITY

11.5.1. Privacy

Personal and medical information relating to research participants will be treated as confidential. The risk of disclosure will be minimized by secure storage of documents and use of linked data by replacing personal identifiers with a unique study code to conceal the identity of the patient.

11.5.2. Privacy of individual

Individual data such as tests for malaria and anaemia will be reported to the participant at point of care, to relevant study staff and where appropriate will be recorded in the patients' medical record book in addition to study CRFs.

11.5.3. Confidentiality of data

All information regarding the participants will remain confidential to the extent allowed by law. Unique numerical identifiers will be used for data entry. All screening forms and case report forms will be kept in a secured location with access limited to authorized study staff. Unique numerical identifiers will be used for the computer-based data entry and blood samples. Publications will contain only aggregate data. No identifying information will be included.

11.6. DECLARATION OF INTEREST

None of the chief or principal investigators have paid consultancies with the pharmaceutical companies involved in the trial, or other competing interest for the overall trial or in each study site. BR received Norwegian Kroner (NOK) 100,000 (~USD 15000) from GSK in August 2010 and January 2011 for an economic evaluation of second generation pneumococcal conjugate vaccines in Norway.[47,48]

11.7. ACCESS TO SOURCE DATA/DOCUMENTS

In addition to the clinical monitors, authorised representatives of the sponsor/CRO, an IEC/IRB or regulatory authority may visit the study site to perform audits or inspections, including source data verification. The investigator agrees to allow the sponsor and CRO representatives, including the monitor and study safety monitor, the DMEC, the IRB/IEC direct access to source data and other relevant documents.

11.8. RISKS AND BENEFITS

11.8.1. Risks

11.8.1.1. Blood sampling

Blood sampling may be inconvenient to the participants, and may cause minor discomfort and bruising and local infection if not conducted adequately. The volume of blood collected from each participant will be small, not more than 5 ml each time. Well-trained clinicians, nurses and laboratory staff employed on the trial will perform blood-sampling tasks. New and sterile disposable needles and lancets will be used for blood sample collection. Universal precaution measures for blood handling and disposal will be observed when performing the procedures and used needles and other waste will be safely discarded immediately after use.

11.8.1.2. Experience with DP as IPTp

Dihydroartemisinin-piperaquine (DP), the drug combination that will be used in this trial, is one of the artemisinin containing combination therapies (ACTs). ACTs are now the standard for treatment of *P.falciparum* malaria in both adults and children.[49] A systematic review of the efficacy and safety of ACTs for the treatment of malaria in children conducted to inform the 2nd edition of the malaria treatment guidelines by WHO showed that DP is very effective and provides a long duration of post-treatment prophylaxis, similar to mefloquine and longer than amodiaquine (AQ-artesunate, or AQ-SP) and artemether-lumefantrine (Coartem®) based antimalarial combinations.[19,50] Because of its long half-life, piperaquine (PQ) has great potential for use as the ACT of choice for malaria chemoprevention using monthly dosing as was shown in IPT studies in infants, children and adults.[18,34,51,52]

The trial with monthly DP in Thai adults showed it to be well tolerated, safe, and highly effective.[18] In that trial the most important determinant of protective efficacy was the trough plasma concentration of piperaquine, and this was determined by the dosing frequency. Compared with participants receiving monthly DP dosing, participants who received dosing every 2 months were 8 times more likely to get malaria (adjusted hazards ratio [AHR], 8.24; 95% CI, 3.25 to 20.9), and participants in the placebo group were 41 times more likely to get malaria within 9 months (AHR,

41.3; 95% CI, 16.6 to 102.8). Furthermore, a recent study in Ugandan school children also showed that monthly DP given for 12 months was much more effective than DP given once per school term.[33]This suggests that for effective prevention of malaria, DP should be given monthly in order to achieve steady state concentrations above the minimum inhibitory concentrations and sustained prophylactic levels.[18]

PQ is currently only available in the fixed dose combination with dihydro-artemisinin (DHA) as DP. The DHA component, eliminated within a few hours, is not expected to provide a significant contribution to the effect of IPTp, yet may provide a degree of protection against the development of PQ resistance in the parasite population. DP is registered in both Kenya and Uganda, initially as Duo-Cotecxin®, manufactured by Beijing HolleyCotec, and recommended by the Kenyan Ministry of Health as second-line treatment since 2009, and approved in March 2012 by the Ugandan National Drug Authority (NDA) for treatment of malaria and to comply with the Current Good Manufacturing Practice (cGMP) regulations enforced by the Uganda authorities.

Piperaquine is well tolerated. Side effects in adults include transient drops in haemoglobin by day 7 (seen with all artemisinins), headache, weakness and fever. The main safety concerns with piperaquine relate to its dose-dependent QTc prolongation. Transient QTc prolongation has been confirmed in clinical trials, but these were mild and similar to many other anti-malarials[53] and there is no indication from clinical data signalling that it is associated with clinically significant arrhythmias.[34,53,54]This is consistent with recent in-vitro models which confirmed that despite mild QTc prolongation, the potential cardiac proarrhythmic risk with piperaquine is low and similar to that observed with lumefantrine (the long-acting component in Coartem), and lower than for chloroquine. This study concluded that DP does not appear to induce potential torsadogenic effects in vitro (which could result in life threatening abnormality of heart rhythm).

11.8.2. Benefits

11.8.2.1. Anticipated benefits to study participants

By taking part in this trial, participants will be regularly monitored free of charge, improving the likelihood that any malaria infection or anaemia will be detected and treated. For expenses reimbursement and incentives see section 11.10, page 61

11.8.2.2. Benefit to the community

This project is designed to generate the information required to assess whether PMC should be recommended as a cost-effective strategy for the post-discharge management of children with severe anaemia in malaria endemic areas. It is anticipated the trial results will increase the effectiveness of the management of severely ill children and reduce the health care costs associated with high readmission rates post-discharge, thereby also reducing associated costs to poor households. Women, who are the primary caretakers and also key actors for household's economic development, will be relieved from having to take care of ill children and consequently improved welfare for households is expected. In the longer term, the ultimate beneficiaries of this research will be the children in sub-Saharan Africa, whose quality of life, health, welfare and creative output will be enhanced.

11.9. ANCILLARY AND POST-TRIAL CARE

11.9.1. Health care during the trial

All care directly related to the proper and safe conduct of the trial, and the treatment of immediate adverse events related to trial procedures will be provided free of charge by the local hospitals. The provision of ancillary care beyond that immediately required for conduct of the trial will not be covered by the trial. The use of a health passport or national equivalent will aid the identification of study children.

11.9.2. Trial insurance

The sponsor will take out trial insurance such that participants enrolled into the study are covered by indemnity for negligent harm and non-negligent harm associated with the protocol. This will include cover for additional health care, compensation or damages whether awarded voluntarily by the Sponsor, or by claims pursued through the courts. The liability of the manufacturer of the trial drug DP is limited to those claims arising from faulty manufacturing of the commercial product and not to any aspects of the conduct of the study.

11.9.3. Post-trial care

The study budget is not in a position to fund post-study care or implementation of PMC as policy. However, the investigators work in close collaboration with local and international policy makers (e.g. WHO) and funders(e.g. President Malaria Initiative) to ensure that policy makers and funders are informed early of germane research finding and can plan for the potential implementation of PMC as policy in the study areas and other relevant areas in Kenya and Uganda (see also section 12, Dissemination and application of the results, page 62).

11.10. EXPENSES REIMBURSEMENT AND INCENTIVES

The study will provide payment for all study drugs, study procedures, study-related visits and reasonable medical expenses that are incurred in study clinics or hospitals as a result of the study, including expenses for transport for any study related visits including unscheduled visits in between scheduled visits to study clinics. The study will not cover the costs of scheduled or unscheduled surgery or trauma related events (e.g. accidents, burns etc) if this is not deemed to be related to the study by the principal investigators or their representative.

Table 5: Reimbursement of expenses and incentives provided by the study

To Who	*What*	*Approximate Amount*	
		Kenya	Uganda
Hospital	Improvement of infrastructure where required	~$5-20,000/hospital	
	Training of routine staff paediatric ward	~ $2,000/hospital	
	Study procedure costs and study drugs	~ $100,000/hospital	
Caretakers	Travel expenses for caretaker & participant as per KEMRI guidelines*	Up to $5 (~500 Ksh) per person per round trip based on distance as per KEMRI guidelines	Up to ~10 USD (USH 25,000) per round trip based on distance*

Compensation for time as per Uganda National Council for Science and Technology (UNCST) guidelines		Up to ~4 USD (USH 10,000) per patient per scheduled follow-up visit
The study will cover the costs of accomodation and meals if they have to stay overnight, as per KEMRI guidelines	Up to $ 2.8 (about 250 KsH)) per person per day	
Free healthcare in study clinics for study child through provision of a health passport or equivalent, valid for 6 months	For 6 months	

*In exceptional cases higher amounts of travel expenses can be reimbursed if distance requires. This would need to be decided on a case by case basis, courtesy of the site PI/coordinator.

12. DISSEMINATION AND APPLICATION OF THE RESULTS

12.1.1. Result dissemination and publication policy

This study is part of the activities of the PMC Consortium funded through the Norwegian GLOBVAC programme to conduct this and these ancillary studies in Malawi, Uganda and Kenya aimed at generating the evidence needed by the World Health Organisation to consider PMC as a strategy to reduce post-discharge morbidity and mortality in malaria endemic areas in Africa.

At the end of the trials, the results will first be disseminated to national policy makers, government departments, academics from local research institutions and universities, and professional bodies in Kenya and Uganda at the national stakeholders' meeting to be held in each country. Subject to the findings of the study and based on consensus emerging at these meetings, project partners in Kenya and Uganda will support national policy makers to develop the necessary tools and guidelines to guide national and district level health providers to implement the PMC strategy within hospital services and the health system more broadly.

Research results will also be disseminated to the global malaria research community, technical agencies, and international government bodies via peer reviewed journals and at international scientific fora, including the annual American Society of Tropical Medicine and Hygiene (ASTMH) meeting, the annual GLOBVAC conference, and via meetings at WHO in Geneva comprised of leading scientists in the field of malaria.

We will also inform other international organisations and funders of large scale malaria control initiatives including DFID, USAID and the US President's Malaria Initiative (PMI) which aim to improve malaria at regional and local levels and are instrumental in supporting countries to implement malaria control policies in Africa.

12.1.2. Impact

This project is designed to generate the information required to assess whether PMC should be recommended as a cost-effective strategy for the post-discharge management of children with severe anaemia in malaria endemic areas. In the longer term, the ultimate beneficiaries of this research will be the children in Sub-Saharan Africa, whose quality of life, health and creative output will be enhanced.

WHO will be an important stakeholder as the body responsible for setting global health policy and priorities for future investments in health. The project will establish an PMC taskforce to liaise with the Global Malaria Programme (GMP) at WHO and produce the necessary evidence dossiers required by its standing Technical Expert Group (TEG) on malaria chemotherapy or an independent evidence review group (ERG) appointed by WHO. The TEG and ERGs are responsible for reporting its recommendations to the recently established Malaria Policy Advisory Committee (MPAC), which in turns provides independent strategic advice and technical input to WHO for the development of policies related to malaria control and elimination. The PMC dossier will be modelled on the dossier provided to WHO for the IPTc taskforce and will include reports of trial results, the meta-analysis and the mathematical modelling studies. This proposal reflects ongoing discussions with colleagues at GMP and WHO who will be kept informed of progress throughout the project.

12.1.3. Training, Fellowships and Capacity Building

Research capacity in research partner institutes in Kenya, Uganda will be enhanced by provision of training and mentorship for research staff. By running this trial, capacity in trial management will be enhanced. The research study will strengthen the clinical skills of health workers in managing patients. There will be 2 PhD candidates who will conduct their research as part of this project, one each from Uganda and one from Kenya. Partners from the different institutions forming this research network will jointly supervise them.

In addition, the two laboratories of Dr. Bailey and Dr. Juliano at the University of Massachusetts and University of North Carolina respectively have extensive experience in terms of all the novel laboratory and computational aspects described in section 8.7.3.5, page 38 and provide a great opportunity for training of a Kenyan or Ugandan scientist to further develop capacity in bioinformatics and genetic analysis techniques. This includes the next-generation sequencing sample preparation using the molecular inversion probes (MIP) technique initially developed for autism spectrum disorders in the Shendure and Eichler labs of the University of Washington, Seattle (O'Roak et al., 2012, Hiatt et al., 2013) and recently adapted to malaria parasite sequencing (Bailey, unpublished), and high throughput sequencing including deep sequencing techniques using massively parallel sequencing to conduct the molecular analysis of samples.

12.1.4. Authorship and publications

The study will have a publications committee that will be part of the PMC Consortium created by the partners funded through the Norwegian Globvac programme to conduct this trial and ancillary studies in Malawi, Uganda and Kenya aimed at generating the evidence needed by the World Health Organisation to consider PMC/IPTpd as a strategy to reduce post-discharge morbidity and mortality in malaria endemic areas in Africa. The core membership of the Publication Committee will consist of the Consortium grant holders (KP and BR), the Chief Investigator of this trial (FtK), country co-principal investigators (Kenya MD and SK, Uganda RO and RI) from each participating country and

the leads of the ancillary studies (BR and MC). For each manuscript a writing committee will be formed. Each participating country group will be requested to suggest and justify names for authors in addition to the CI and PIs, to be reviewed by the publications committee. Potential site authors could include all professionals that have participated in the trial for a minimum of one year. It is anticipated that for the publication of the results of the main trial, the CI will have last authorship and that a Phd student or otherwise one of the country Co-PIs will have lead authorship. It is also anticipated that the PI of an ancillary study should be considered for the first choice for lead or last author of material derived from this study. Authorship of any presentations or publications arising from this study will also be governed by the principles for authorship criteria of the International Committee of Medical Journal Editors has designed.[55]Disputes regarding authorship will be settled by the CI, PIs and chair of the publications committee of the PMC Consortium. The manufacturer of the study medication will be provided with a draft of the manuscript but will have no role in review, data interpretation, or writing of the article.

12.1.5. Data Sharing Statement

The full protocol will be available on request to any interested professional and may be published in a peer reviewed journals or deposited in an online repository. Individual, de-identified participant data will be made available for meta-analyses as soon as the data analysis is completed, with the understanding that results of the meta-analysis will not be published prior to the results of the individual trial without prior agreement of the investigators. No later than 5 years after the publication of the trial a fully de-identified data set will be available for sharing purposes. All requests for data for secondary analysis will be considered by the publication committee of the PMC Consortium.

13. REFERENCES

1. Calis JC, Phiri KS, Faragher EB, Brabin BJ, Bates I, Phiri AI, Malange P, Khoka M, Beld MG, Teo YY, Rockett KA, Richardson A, Kwiatkowski DP, Molyneux ME, van Hensbroek MB, 2008. Severe anemia in Malawian children. The New England journal of medicine 358: 888-99.
2. Phiri KS, Calis JC, Faragher B, Nkhoma E, Ng'oma K, Mangochi B, Molyneux ME, van Hensbroek MB, 2008. Long term outcome of severe anaemia in Malawian children. PloS one 3: e2903.
3. Bojang KA, Palmer A, Boele van Hensbroek M, Banya WA, Greenwood BM, 1997. Management of severe malarial anaemia in Gambian children. Trans R Soc Trop Med Hyg 91: 557-61.
4. Bojang KA, Milligan PJ, Conway DJ, Sisay-Joof F, Jallow M, Nwakanma DC, Abubakr I, Njie F, Greenwood B, 2010. Prevention of the recurrence of anaemia in Gambian children following discharge from hospital. PLoS One 5: e11227.
5. Zucker JR, Lackritz EM, Ruebush TK, 2nd, Hightower AW, Adungosi JE, Were JB, Metchock B, Patrick E, Campbell CC, 1996. Childhood mortality during and after hospitalization in western Kenya: effect of malaria treatment regimens. Am J Trop Med Hyg 55: 655-60.
6. Zucker JR, Ruebush TK, 2nd, Obonyo C, Otieno J, Campbell CC, 2003. The mortality consequences of the continued use of chloroquine in Africa: experience in Siaya, western Kenya. Am J Trop Med Hyg 68: 386-90.
7. Phiri K, Esan M, van Hensbroek MB, Khairallah C, Faragher B, Ter Kuile FO, 2012. Intermittent preventive therapy for malaria with monthly artemether-lumefantrine for the post-discharge management of severe anaemia in children aged 4-59 months in southern Malawi: a multicentre, randomised, placebo-controlled trial. Lancet Infect Dis 12: 191-200.

8. van Hensbroek MB, Jonker F, Bates I, 2011. Severe acquired anaemia in Africa: new concepts. British journal of haematology: 1365-2141.
9. Price RN, Simpson JA, Nosten F, Luxemburger C, Hkirjaroen L, ter Kuile F, Chongsuphajaisiddhi T, White NJ, 2001. Factors contributing to anemia after uncomplicated falciparum malaria. Am J Trop Med Hyg 65: 614-22.
10. Cisse B, Sokhna C, Boulanger D, Milet J, Ba el H, Richardson K, Hallett R, Sutherland C, Simondon K, Simondon F, Alexander N, Gaye O, Targett G, Lines J, Greenwood B, Trape JF, 2006. Seasonal intermittent preventive treatment with artesunate and sulfadoxine-pyrimethamine for prevention of malaria in Senegalese children: a randomised, placebo-controlled, double-blind trial. Lancet 367: 659-67.
11. White NJ, 2005. Intermittent presumptive treatment for malaria. PLoS medicine 2: e3.
12. ter Kuile FO, van Eijk AM, Filler SJ, 2007. Effect of sulfadoxine-pyrimethamine resistance on the efficacy of intermittent preventive therapy for malaria control during pregnancy: a systematic review. JAMA 297: 2603-16.
13. World Health Organization, 2012. Intermittent Preventive Treatment of Malaria in Pregnancy Using Sulfadoxine-Pyrimethamine (IPTp-SP); Updated WHO Policy Recommendation (October 2012). Updated WHO Policy Recommendation (October 2012). Geneva: World Health Organization.
14. Aponte JJ, Schellenberg D, Egan A, Breckenridge A, Carneiro I, Critchley J, Danquah I, Dodoo A, Kobbe R, Lell B, May J, Premji Z, Sanz S, Sevene E, Soulaymani-Becheikh R, Winstanley P, Adjei S, Anemana S, Chandramohan D, Issifou S, Mockenhaupt F, Owusu-Agyei S, Greenwood B, Grobusch MP, Kremsner PG, Macete E, Mshinda H, Newman RD, Slutsker L, Tanner M, Alonso P, Menendez C, 2009. Efficacy and safety of intermittent preventive treatment with sulfadoxine-pyrimethamine for malaria in African infants: a pooled analysis of six randomised, placebo-controlled trials. Lancet 374: 1533-42.
15. Wilson AL, Taskforce IP, 2011. A systematic review and meta-analysis of the efficacy and safety of intermittent preventive treatment of malaria in children (IPTc). PLoS One 6: e16976.
16. World Health Organization, 2013. Seasonal malaria chemoprevention with sulfadoxine–pyrimethamine plus amodiaquine in children: a field guide.: world Health Organization, Geneva.
17. Steketee RW, Slutsker L, 2011. Targeting of intermittent preventive treatment for malaria. Lancet Infect Dis 12(3): 168-9.
18. Lwin KM, Phyo AP, Tarning J, Hanpithakpong W, Ashley EA, Lee SJ, Cheah P, Singhasivanon P, White NJ, Lindegardh N, Nosten F, 2012. Randomized, Double-Blind, Placebo-Controlled Trial of Monthly versus Bimonthly Dihydroartemisinin-Piperaquine Chemoprevention in Adults at High Risk of Malaria. Antimicrob Agents Chemother: Mar;56(3):1571-7. Epub 2012 Jan 1.
19. Sinclair D, Zani B, Donegan S, Olliaro P, Garner P, 2009. Artemisinin-based combination therapy for treating uncomplicated malaria. Cochrane Database Syst Rev: CD007483.
20. Akpaloo W, Purssell E, 2014. Does the Use of Dihydroartemisinin-Piperaquine in Treating Patients with Uncomplicated falciparum Malaria Reduce the Risk for Recurrent New falciparum Infection More Than Artemether-Lumefantrine? Malar Res Treat 2014.
21. Zani B, Gathu M, Donegan S, Olliaro PL, Sinclair D, 2014. Dihydroartemisinin-piperaquine for treating uncomplicated Plasmodium falciparum malaria. Cochrane Database Syst Rev 1: CD010927.
22. Clerk CA, Bruce J, Affipunguh PK, Mensah N, Hodgson A, Greenwood B, Chandramohan D, 2008. A randomized, controlled trial of intermittent preventive treatment with sulfadoxine-pyrimethamine, amodiaquine, or the combination in pregnant women in Ghana. The Journal of infectious diseases 198: 1202-11.
23. Luxemburger C, Price RN, Nosten F, Ter Kuile FO, Chongsuphajaisiddhi T, White NJ, 1996. Mefloquine in infants and young children. Ann Trop Paediatr 16: 281-6.

24. ter Kuile FO, Nosten F, Luxemburger C, Kyle D, Teja-Isavatharm P, Phaipun L, Price R, Chongsuphajaisiddhi T, White NJ, 1995. Mefloquine treatment of acute falciparum malaria: a prospective study of non-serious adverse effects in 3673 patients. Bull World Health Organ 73: 631-42.
25. Ashley EA, Dhorda M, Fairhurst RM, Amaratunga C, Lim P, Suon S, Sreng S, Anderson JM, Mao S, Sam B, Sopha C, Chuor CM, Nguon C, Sovannaroth S, Pukrittayakamee S, Jittamala P, Chotivanich K, Chutasmit K, Suchatsoonthorn C, Runcharoen R, Hien TT, Thuy-Nhien NT, Thanh NV, Phu NH, Htut Y, Han KT, Aye KH, Mokuolu OA, Olaosebikan RR, Folaranmi OO, Mayxay M, Khanthavong M, Hongvanthong B, Newton PN, Onyamboko MA, Fanello CI, Tshefu AK, Mishra N, Valecha N, Phyo AP, Nosten F, Yi P, Tripura R, Borrmann S, Bashraheil M, Peshu J, Faiz MA, Ghose A, Hossain MA, Samad R, Rahman MR, Hasan MM, Islam A, Miotto O, Amato R, MacInnis B, Stalker J, Kwiatkowski DP, Bozdech Z, Jeeyapant A, Cheah PY, Sakulthaew T, Chalk J, Intharabut B, Silamut K, Lee SJ, Vihokhern B, Kunasol C, Imwong M, Tarning J, Taylor WJ, Yeung S, Woodrow CJ, Flegg JA, Das D, Smith J, Venkatesan M, Plowe CV, Stepniewska K, Guerin PJ, Dondorp AM, Day NP, White NJ, Tracking Resistance to Artemisinin C, 2014. Spread of artemisinin resistance in Plasmodium falciparum malaria. N Engl J Med 371: 411-23.
26. Obonyo CO, Vulule J, Akhwale WS, Grobbee DE, 2007. In-hospital morbidity and mortality due to severe malarial anemia in western Kenya. Am J Trop Med Hyg 77: 23-8.
27. Gutman J, Kovacs S, Dorsey G, Stergachis A, ter Kuile FO, 2015. The safety, tolerability and efficacy of repeated doses of dihydroartemisinin-piperaquine for the prevention and treatment of malaria: A systematic review and meta-analysis. Report prepared for Evidence Review Group (ERG) meeting, WHO Geneva, 13-16 July 2015.
28. Bojang K, Akor F, Bittaye O, Conway D, Bottomley C, Milligan P, Greenwood B, 2010. A randomised trial to compare the safety, tolerability and efficacy of three drug combinations for intermittent preventive treatment in children. PLoS One 5: e11225.
29. Cisse B, Cairns M, Faye E, O ND, Faye B, Cames C, Cheng Y, M ND, Lo AC, Simondon K, Trape JF, Faye O, JL ND, Gaye O, Greenwood B, Milligan P, 2009. Randomized trial of piperaquine with sulfadoxine-pyrimethamine or dihydroartemisinin for malaria intermittent preventive treatment in children. PLoS One 4: e7164.
30. Zongo I, Milligan P, Compaore YD, Some AF, Greenwood B, Tarning J, Rosenthal PJ, Sutherland C, Nosten F, Ouedraogo JB, 2015. Randomized non-inferiority trial of dihydroartemisinin-piperaquine compared with sulfadoxine-pyrimethamine plus amodiaquine for SMC in Burkina Faso. Antimicrob Agents Chemother.
31. Kamya MR, Kapisi J, Bigira V, Clark TD, Kinara S, Mwangwa F, Muhindo MK, Kakuru A, Aweeka FT, Huang L, Jagannathan P, Achan J, Havlir DV, Rosenthal PJ, Dorsey G, 2014. Efficacy and safety of three regimens for the prevention of malaria in young HIV-exposed Ugandan children: a randomized controlled trial. AIDS 28: 2701-9.
32. Lwin KM, Phyo AP, Tarning J, Hanpithakpong W, Ashley EA, Lee SJ, Cheah P, Singhasivanon P, White NJ, Lindegardh N, Nosten F, 2012. Randomized, double-blind, placebo-controlled trial of monthly versus bimonthly dihydroartemisinin-piperaquine chemoprevention in adults at high risk of malaria. Antimicrob Agents Chemother 56: 1571-7.
33. Nankabirwa JI, Wandera B, Amuge P, Kiwanuka N, Dorsey G, Rosenthal PJ, Brooker SJ, Staedke SG, Kamya MR, 2014. Impact of intermittent preventive treatment with dihydroartemisinin-piperaquine on malaria in Ugandan schoolchildren: a randomized, placebo-controlled trial. Clin Infect Dis 58: 1404-12.
34. Bigira V, Kapisi J, Clark TD, Kinara S, Mwangwa F, Muhindo MK, Osterbauer B, Aweeka FT, Huang L, Achan J, Havlir DV, Rosenthal PJ, Kamya MR, Dorsey G, 2014. Protective efficacy and safety of three antimalarial regimens for the prevention of malaria in young ugandan children: a randomized controlled trial. PLoS Med 11: e1001689.

35. World Health Organization, 2003. International Statistical Classification of Diseases and Related Health Problems, 10th Revision, Version for 2003: World Health Organization, Geneva.
36. Phiri K, Esan M, van Hensbroek MB, Khairallah C, Faragher B, Ter Kuile FO, 2011. Intermittent preventive therapy for malaria with monthly artemether-lumefantrine for the post-discharge management of severe anaemia in children aged 4-59 months in southern Malawi: a multicentre, randomised, placebo-controlled trial. Lancet Infect Dis.
37. Boivin MJ, Bangirana P, Byarugaba J, Opoka RO, Idro R, Jurek AM, John CC, 2007. Cognitive impairment after cerebral malaria in children: a prospective study. Pediatrics 119: e360-6.
38. Bangirana P, Opoka RO, Boivin MJ, Idro R, Hodges JS, Romero RA, Shapiro E, John CC, 2014. Severe malarial anemia is associated with long-term neurocognitive impairment. Clin Infect Dis 59: 336-44.
39. Rothman KJ, Greenland S, Lash TL, 2008. Modern Epidemiology, 3rd edition. Philadelphia: Wolters Kluwer, Lippincott Williams & Wilkins.
40. Bailey JA, Mvalo T, Aragam N, Weiser M, Congdon S, Kamwendo D, Martinson F, Hoffman I, Meshnick SR, Juliano JJ, 2012. Use of massively parallel pyrosequencing to evaluate the diversity of and selection on Plasmodium falciparum csp T-cell epitopes in Lilongwe, Malawi. J Infect Dis 206: 580-7.
41. Parobek CM, Bailey JA, Hathaway NJ, Socheat D, Rogers WO, Juliano JJ, 2014. Differing patterns of selection and geospatial genetic diversity within two leading Plasmodium vivax candidate vaccine antigens. PLoS Negl Trop Dis 8: e2796.
42. Taylor SM, Parobek CM, DeConti DK, Kayentao K, Coulibaly SO, Greenwood BM, Tagbor H, Williams J, Bojang K, Njie F, Desai M, Kariuki S, Gutman J, Mathanga DP, Martensson A, Ngasala B, Conrad MD, Rosenthal PJ, Tshefu AK, Moormann AM, Vulule JM, Doumbo OK, Ter Kuile FO, Meshnick SR, Bailey JA, Juliano JJ, 2014. Absence of Putative Artemisinin Resistance Mutations Among Plasmodium falciparum in Sub-Saharan Africa: A Molecular Epidemiologic Study. J Infect Dis.
43. O'Roak BJ, Vives L, Fu W, Egertson JD, Stanaway IB, Phelps IG, Carvill G, Kumar A, Lee C, Ankenman K, Munson J, Hiatt JB, Turner EH, Levy R, O'Day DR, Krumm N, Coe BP, Martin BK, Borenstein E, Nickerson DA, Mefford HC, Doherty D, Akey JM, Bernier R, Eichler EE, Shendure J, 2012. Multiplex targeted sequencing identifies recurrently mutated genes in autism spectrum disorders. Science 338: 1619-22.
44. Hiatt JB, Pritchard CC, Salipante SJ, O'Roak BJ, Shendure J, 2013. Single molecule molecular inversion probes for targeted, high-accuracy detection of low-frequency variation. Genome Res 23: 843-54.
45. US Department of Health and Human Services; National Institutes of Health; National Institute of Allergy and Infectious Diseases; Division of AIDS, 2014. Division of AIDS (DAIDS) Table for Grading the Severity of Adult and Pediatric Adverse Events, Version 2.0. [November 2014]. Available from: http://rsc.tech-res.com/Document/safetyandpharmacovigilance/DAIDS_AE_GRADING_TABLE_v2_NOV2014.pdf.
46. National Institute for Health Research - National Health Services UK (NHS), 2014. CT-toolokit. Available at: http://www.ct-toolkit.ac.uk/glossary/suspected-unexpected-serious-adverse-reactions-susar. Accessed 15 September 2014, 2014.
47. Robberstad B, Akselsen PE, Kvaerner KJ, Berstad AK, 2013. Response to "reply to: economic evaluation of second generation pneumococcal conjugate vaccines in Norway". Vaccine 31: 442-3.
48. Robberstad B, Frostad CR, Akselsen PE, Kvaerner KJ, Berstad AK, 2011. Economic evaluation of second generation pneumococcal conjugate vaccines in Norway. Vaccine 29: 8564-74.
49. World Health Organization, 2010. Guidelines for the treatment of malaria -- 2nd edition. Geneva, Switzerland.

50. World Health Organization, 2010. Guidelines for the treatment of malaria -- 2nd edition. Geneva, Switzerland: WHO Press, World Health Organization.
51. Cisse B, Cairns M, Faye E, O ND, Faye B, Cames C, Cheng Y, Simondon K, Trape JF, Faye O, JL ND, Gaye O, Greenwood B, Milligan P, 2009. Randomized trial of piperaquine with sulfadoxine-pyrimethamine or dihydroartemisinin for malaria intermittent preventive treatment in children. PloS one 4: e7164.
52. Cisse B, Cairns M, Faye E, O ND, Faye B, Lo AC, Trape JF, Faye O, Gaye O, Greenwood B, Milligan P, 2009. Randomized trial of piperaquine with sulfadoxine-pyrimethamine or dihydroartemisinin for malaria intermittent preventive treatment in children. PloS one 4: e7164.
53. Mytton OT, Ashley EA, Peto L, Price RN, La Y, Hae R, Singhasivanon P, White NJ, Nosten F, 2007. Electrocardiographic safety evaluation of dihydroartemisinin piperaquine in the treatment of uncomplicated falciparum malaria. Am J Trop Med Hyg 77: 447-50.
54. Keating GM, 2012. Dihydroartemisinin/Piperaquine: A Review of its Use in the Treatment of Uncomplicated Plasmodium falciparum Malaria. Drugs 72: 937-61.
55. 2010. Uniform requirements for manuscripts submitted to biomedical journals: Writing and editing for biomedical publication. J Pharmacol Pharmacother 1: 42-58.

14. FINANCIAL ASPECTS AND CONFLICT OF INTEREST

14.1.1. Funding of the trial

Funding to conduct the trial is provided by the University of Bergen, Norway through a grant from the Research Council of Norway, Global Health and Vaccination Research (GLOBVAC) programme, number 234487.The Liverpool School of Tropical Medicine is providing salary support for FtK. The US Centers for Disease control and Prevention (CDC) is providing salary support for MD, based in Kenya, and infrastructural support for the trial conduct in Kenya and centralised data management.

GLOBVAC had no role in the design of this trial and will not have any during the execution, analysis, interpretation of the data, or decision to submit the results

14.1.2. Provision of the study drugs

Dihydroartemisinin-piperaquine and potentially the placebo will be provided by Sigma Tau**,** the manufacturer. The study will provide copies of safety reports of SAEs and AEs to the manufacturer (expedited where required). The manufacturer will not be involved in the design of the trial.

15. BUDGET AND BUDGET JUSTIFICATION

See Appendix V. Budget, Page 119 and Appendix VI. Budget Justification, Page 119

16. APPENDICES

16.1. APPENDIX I. ROLE INVESTIGATORS AND NON-ENGAGED COLLABORATORS

16.1.1. Protocol development: authors' contributions

Feiko ter Kuile (FtK) and Kamija Phiri (KP) conceived the study. RI, RO, and CJ and FtK drafted the protocol. Bjarne Robberstad (BR), Richard Idro (RI), Robert Opoka (RO), Chandy John (CJ), Meghna Desai (MD), Simon Kariuki (SK), Azra Ghani (AG), Michael Boele van Hensbroek (MBvH) and FtK and KP, further development the study design during a protocol workshop. KP and BR are the GLOBVAC grant holders. Brian Faragher (BF) provided statistical expertise in clinical trial design. All authors contributed to the refinement of the study protocol and approved the final version.

16.1.2. Role Investigators

This multi-centre trial will have one chief-investigator and four co-principal investigators, 2 per country. Prof Feiko ter Kuile from Liverpool School of Tropical Medicine (LSTM), will be the Chief Investigator and carry overall responsibility for the coordination of the trial and for the linkages with the sponsor, funders and with WHO. The fieldwork in Kenya will be led by the co-Principal Investigators Dr Simon Kariuki, head Malaria Branch of the KEMRI–CDC Collaboration at KEMRI, Kisumu, and Dr Titus Kwambai a medical officer from the Ministry of Health, assigned to KEMRI for the duration of this study. In Uganda, the study will be led by Dr Richard Idro and Dr Robert Opoka, senior paediatricians from the College of Health Sciences at Makerere University in Kampala. They will be supported by Prof Chandy John, a paediatrician from the University of Minnesota.

In Uganda, Dr Richard Idro and Dr Robert Opoka will be co-supervisor for a Ugandan PhD student (Dr Aggrey Dhabangi) together with Prof Michael Boele van Hensbroek, Professor in Global Child Health, University of Amsterdam, and Prof Chandy John. Dr Tom Ediamu, Dr Sophie Namasopo, Dr Harriet Nambuya from Hoima, Jinja regional referral hospitals and Tororo hospital will be the link persons with the local hospital in Uganda.

In Kenya, the field work will be coordinated by a Kenyan PhD student (Dr Titus Kwambai) who will be supervised by Prof ter Kuile, and Dr Desai and Dr Kariuki. He/She will be supported by Dr Martina Oneke, a senior paediatrician at KEMRI. Dr Grace Nalwa, and Magdalene Kuria from Migori and Kisumu County referall hospitals will be responsible for the linkage with local hospital in western Kenya. The team will receive technical support from Dr Aaron Samuels, a senior clinical epidemiologist with many years of clinical research experience, from the Malaria Branch, CDC Atlanta and based in Kenya.

Statistical support will be provided by Prof Duolao Wang and Prof Brian Faragher, the trial statistician based at LSTM. Prof Jon Juliano will genotype key host genetic factors and examine parasite diversity along with drug resistance genes in their respective laboratories in the University

of Massachusetts and North Carolina, USA. This genetic data will be incorporated into the overall statistical framework.

Prof Kamija Phiri, a senior clinical epidemiologist from the College of Medicine (CoM) in Malawi with over 10 years' experience in clinical research and PI of the first IPTpd trial in Malawi[7] and PI of a GlobVac funded PMC delivery-mechanism trial in Malawi, will provide technical support to the design and conduct of the study. He will also co-chair the PMC Consortium meetings and coordinate linkages with the PMC Consortium's delivery trial in Malawi and with policy makers in Malawi, Kenya and Uganda. Prof Phiri, together with Prof Bjarne Robberstad from the University of Bergen will be the Project Owners and grant holder of the PMC Consortium Grant from the Research Council of Norway, Global Health and Vaccination Research (GLOBVAC) programme. Prof Robberstad will be responsible for health economics component of the trial.

16.1.3. Role Non-Engaged Collaborators

Non engaged collaborators are not classified as investigators and have an advisory role only and are not to be directly involved with the research activities.

16.2. APPENDIX II. PARTICIPANT INFORMATION SHEETS AND INFORMED CONSENT FORMS

16.2.1. Participant Information Sheet for main trial (English)

Post-Discharge Malaria Chemoprevention
PMC Study

Participant Information Sheet for trial

Title: Malaria Chemoprevention with monthly treatment with dihydroartemisinin-piperaquine for the post-discharge management of severe anaemia in children aged less than 5 years in Uganda and Kenya: A 3-year, multi-centre, parallel-group, two-arm randomised placebo controlled superiority trial

Investigators

Dr Richard Idro[3]
Dr Robert Opoka[3]
Dr. Aggrey Dhabangi[3]
Dr Tom Ediamu[4]
Dr Sophie Namasopo[5]
Dr Harriet Nambuya[5]
Dr Simon Kariuki[2]
Dr Titus Kwambai[2]
Dr Martina Oneko[2]
Dr Grace M Nalwa[7]
Dr Magdalene Kuria[8]
Dr Aaron Samuels[2,9]
Prof Kamija Phiri[12]
Prof Bjarne Robberstad[13]
Prof Brian Faraghar[1]
Prof Duolao Wang[1]
Prof Chandy John[14]
Prof Jonathan J. Juliano[15]
Prof Jeff Bailey[16]
Prof Feiko ter Kuile[1,2]

Institutions

1. Liverpool School of Tropical Medicine (LSTM), Liverpool, United Kingdom
2. KEMRI Centre for Global Health Research (CGHR), Kisian, Kenya
3. College of Health Sciences, Makerere University, Kampala Uganda
4. Hoima Regional Referral Hospital, Hoima, Uganda
5. Jinja Regional referral hospital, Uganda
6. Tororo Hospital, Tororo, Uganda
7. Migori County Referral Hospital, Migori, Kenya
8. Kisumu County Referral Hospital, Kisumu, Kenya
9. Division of Parasitic Diseases and Malaria, US Center for Disease Control and Prevention (CDC), Atlanta, GA, USA
10. National Malaria Control Program, Ministry of Health Kenya, Nairobi, Kenya
11. Ministry of Health, Siaya County, Siaya, Kenya
12. College of Medicine, University of Malawi, Blantyre, Malawi
13. Centre for International Health, &Department of Global Public Health and Primary Care, University of Bergen, Bergen, Norway
14. Ryan White Center for Pediatric Infectious Disease and Global Health, Indiana University School of Medicine, Indianapolis, IN, USA
15. Division of Infectious Diseases, School of Medicine, University of North Carolina, Chapel Hill, USA
16. Department of Medicine Division of Transfusion Medicine and Program in Bioinformatics and Integrative Biology, University of Massachusetts School of Medicine, Worcester, USA

Purpose of this study

KEMRI/CDC and Makerere University are working with the Kenyan and Ugandan Ministries of Health to conduct a malaria research project to find better ways to reduce the risk of children dying or being readmitted to hospital after treatment for severe lack of blood (severe anaemia).

What is the study about?

Children living in malaria regions who have been treated for severe anaemia have higher chances of falling sick again or even dying within three months after leaving the hospital.

The aim of this study is to find out the best way to prevent children who have been treated for severe anaemia from dying or having to be admitted again. We want to know if taking a new drug called DHA-piperaquine (DP) at 2, 6, and 10 weeks after leaving the hospital is better than taking Coartem® at discharge only.

Why has your child been chosen?

Your child was admitted and treated in hospital because he or she was very sick from lack of blood. Many children who are in this hospital with this same illness will also be invited to take part. In total we will ask 2212 children who are under 5 years of age to take part in the research.

What will happen to my child if he/ she takes part?

If you decide to take part, we will ask you some questions about your child's illness and the treatment he/she received before you came to hospital and how much it cost. We will also ask you questions about where you live, your travel time and how much it cost you to come to the hospital, the structure of your house, your education and the main sources of income for the family.

A study doctor or nurse will examine your child again. We will also copy the information about your child's illness from the hospital's clinic and laboratory notes.

What will happen to blood samples taken in this study?

We will draw a small amount of extra blood (1 teaspoonful) from the vein in the arm of your child. The blood sample will be used to test your child for malaria and lack of blood. This can be done on site and results and we can share the results with you.

In the future, we will also look at factors that protect against malaria and cause lack of blood, e.g. sickle cell disease and thalassemia. We will test the blood sample to see if the malaria drugs are still working. We will also test whether the malaria parasites have changed with time.

How about the study treatment?

For the purpose of this study we shall give all children Coartem once they are well enough to eat again, even if they were not found to have malaria on admission to hospital. Our study nurse will give your child the first dose of the Coartem here in the hospital. If your child is well enough to leave the hospital, he/she has to take the rest of medicine at home until 3 days' medicine is completed.

What will happen after I leave the hospital?

We will ask you to come back to the hospital after about 2 weeks for follow-up. At that time, we shall put your child on the study treatment.

How will they decide what treatment my child will get?

There are two types of treatments, DP and a placebo (a drug that looks exactly like the DP but does not contain any medicine). Your child will be assigned to receive one type. The choice of which study treatment your child will get will be by chance. The two study treatments look exactly the same so even the study clinicians treating your child will not know which of the two study medicine your child will be taking.

How is the study medicines taken?

The treatment will be given once a day for 3 days. Our study nurse will give your child the first dose of the treatment here in the hospital or at your home. All the children in each of the groups will receive treatment as crushed pills dissolved in syrup. The remaining 2 doses will be taken at home. The study nurse will call to remind you to give your child the medicine if necessary.

Will you do anything else during the week 2 visit?

We will take a small blood sample from your child’s heel or finger to test for lack of blood. In addition, we will also give your child iron tablets as treatment for lack of blood. S/he has to take every day for 1 month.

Do I have to bring my child back?

Yes we will need to see your child again at 6 weeks and again at 10 weeks to give more of the study treatment as described above. We will also call or visit you at 4 to 5 months to find out how your child is doing and to remind you of the last clinic visit at the end of 6 months (26 weeks).

6 month visit

We would like to see your child one last time after 6 month from today to see how well the medicines have worked. At that time, we will ask you questions on your child's health, examine and weigh the child and measure his/her temperature. We will also take a few drops of blood from your child by pricking his/her finger (less than half a teaspoon). The blood will be used to test for malaria and to measure the level of his/her blood.

What do I have to do?

If you decide to join the study we will expect you to be available for the whole period of 6 months from the day you join the study.

It is very important that the child takes all the medicine given by the study. You must never share the medicine with your other children, even if they are also in the study.

Children who take part in the study can only take certain medicines. We therefore ask you not to buy extra medicines for your child from shops during the study. If your child is ill at any time during the 6 months of the study, you can bring your child to the study clinic in the hospital. We shall pay for your transport ([Kenya Ksh 500]/[Uganda the amount will be left blank per local regulations] per trip). [Kenya: We will also cover the costs of accommodation and food for you and your child if you have to the stay overnight] / [Uganda: You will receive some money per visit for your time and food]. We will also pay for all hospital bills related to treatments and in hospital admission fee related to the study.

What are the side effects of the study treatments?
All medicines have side effects**.** There is a chance that your child will vomit after taking the medicines. Vomiting is common, especially when a child has fever. If a child vomits within half an hour another treatment dose will be given. If vomiting occurs after half an hour, half the dose will be repeated. If the vomiting occurs after 1 hour, no extra dose is needed. Please inform the study staff as soon as possible if your child has vomited the dose. For this purpose, we will ask you to remain in the hospital for at least 1 hour after the child has taken the first dose of the study medication.

Generally the study treatments are well tolerated by children. In a few cases children complain of headache, feeling dizzy, or nausea and tummy pain. In almost all children, this is mild. Many of these symptoms can also be caused by malaria.

DP may cause mild changes in heartbeat. We shall therefore monitor your child if s/he complains of pain in the chest or the heart beating fast. We shall do this by taking a heart test (ECG).

What are the risks of taking part in this study?
If you decide to join the study, the risks are minimal. A small bruise or mild pain on the finger or arm from where the blood is taken may develop. There is also a chance of infection at the site where blood is drawn from. This chance is very small because we always use clean materials. The study will require you to make more visits to the hospital than normal. This may be inconvenient. We will reimburse your transport costs.

Are there any benefits for my child?
There is no direct benefit to your child. However we shall pay for all the treatment for your child for the 6 months s/he will be in the study. This does not include surgery, accidents or any other illness not related to the study. Because your child will visit the clinic regularly during the course of the study, your child is likely to have malaria and other illnesses detected and treated more quickly than usual.

What if something goes wrong?
Your child will receive medical care at no cost for any injury or illness which occurs as a result of your child joining this study. In the course of the study, we will inform you if we find a disease or illness that may endanger your child.

Will my taking part in this study be kept confidential?
If you consent to take part in the research study we will keep the name of your child and all the information that we get from you as part of this study private to the extent allowed by the law. Only members of the study staff and people from the safety committee and Government authorities can review the records with your and your child's name on it. We will use the information you give to us only for research. The information collected may be shared with other people in other institutes and countries, but your and your child's name will not appear on any reports.

How much time does it take?
The total period of study participation for your child will be 6 months. This will include a 2 weeks, 6 weeks, 10 weeks, and 6 month visits, as explained above. The follow-up visits will take about 60 to 90 minutes.

Does my child have to take part?

It is up to you to decide if you want your child to take part or not. If you choose for your child to take part you will also be asked to sign a consent form. You will be free to stop at any time without giving a reason. If you do not want your child to take part now, or in the future, this will not affect the standard care your child will receive.

Contact for further information
[Kenya]

1. [If you have any questions about this study, or if you want your child to stop being part of the study, please contact Dr Simon Kariuki (Tel: 057 202 29 02) or Dr Titus Kwambai (Tel: 0723 354 238), KEMRI/CDC, P.O.Box 1578, Kisumu. You can also contact any of our study staff at the hospital.
2. If you have any questions about your rights as a study patient, or if you think your child has been injured because of this study, please contact The Secretary, KEMRI Scientific and Ethics Review Unit, Mbagathi Rd. Nairobi, Kenya, PO Box 54840-00200, Nairobi; Telephone numbers: 020-2722541, 020-2713349, or 0722-205901.]

[Uganda]

1. [If you have any questions about this study, or if you want your child to stop being part of the study, please contact Dr Richard Idro or Dr Robert Opoka on 0774 274173 or 0772996164. You can also contact any of our study staff at the hospital.
2. If you have any questions about your rights as a study patient, or if you think your child has been injured because of this study, please contact Prof James Tumwine, Chairman of the Makerere University School of Medicine Research and Ethics Committee (SOMREC) on 0414530020.]

We hope the results of this study will help to improve the treatment for severe anaemia in this area. Thank you very much for your time.

You will be given a copy of this information sheet and the signed consent form to keep for your records. One copy will be kept by the study staff.

16.2.2. Consent statement for main trial (English)

Post-Discharge Malaria Chemoprevention PMC Study

Consent Statement for trial

Title of Study: Malaria Chemoprevention with monthly treatment with dihydroartemisinin-piperaquine for the post-discharge management of severe anaemia in children aged less than 5 years in Uganda and Kenya: A 3-year, multi-centre, parallel-group, two-arm randomised placebo controlled superiority trial.

Participant ID: ______________________________ (copy assigned ID from the Pre-enrolment CRF)

The above has been explained to me and I agree for my child to take part in the study. I understand that I am free to choose for my child to be in this study and that saying "NO" will have no effect on my child. I agree to answer questions asked by the research team, to have my child examined and a small amount of blood to be drawn from my child's finger/heel and be tested for malaria, anaemia and traits (or genes) that may cause lack of blood or protect them from malaria**.** I understand that my child will participate in the study for a total period of 6 months. I agree to bring my child to the clinic for follow up or for the study staff to visit our home at the 2nd, 6th and 10th weeks and also at the end of the 6th month. I also agree to bring the child to the clinic whenever necessary or for study staff to visit our home or contact me on phone. I understand that relevant sections of my child's health records may be copied and facts collected during the study may be looked at by staff from KEMRI/CDC and/or Makerere University. I give permission for these persons to have access to my child's records and link them to other studies. I also give permission to share the facts collected through this study, without my child's name and address, with other study groups outside Kenya and Uganda.	If you agree circle "YES", if you do not agree circle 'NO'	
	YES	NO

	Name	Signature or left thumbprint	Today's date
Parent providing consent for child			
Witness*			
Study staff consenting participant			

*A parent/guardian can sign with witness, or verbally state his/her consent in the presence of a witness who will then sign.

16.2.3. Participant Information Sheet long-term storage/ future studies (English)

Post-Discharge Malaria Chemoprevention PMC Study

Participant Information Sheet for long term storage of blood samples and future studies

Title: Malaria Chemoprevention with monthly treatment with dihydroartemisinin-piperaquine for the post-discharge management of severe anaemia in children aged less than 5 years in Uganda and Kenya: A 3-year, multi-centre, parallel-group, two-arm randomised placebo controlled superiority trial

Investigators

Dr Richard Idro[3] *Dr Harriet Nambuya[5]* *Dr Magdalene Kuria[8]* *Prof Duolao Wang[1]*
Dr Robert Opoka[3] *Dr Simon Kariuki[2]* *Dr Aaron Samuels[2,9]* *Prof Chandy John[14]*
Dr. Aggrey Dhabangi[3] *Dr Titus Kwambai[2]* *Prof Kamija Phiri[12]* *Prof Jonathan J. Juliano[15]*
Dr Tom Ediamu[4] *Dr Martina Oneko[2]* *Prof Bjarne Robberstad[13]* *Prof Jeff Bailey[16]*
Dr Sophie Namasopo[5] *Dr Grace M Nalwa[7]* *Prof Brian Faraghar[1]* *Prof Feiko ter Kuile[1,2]*

Institutions

1. Liverpool School of Tropical Medicine (LSTM), Liverpool, United Kingdom
2. KEMRI Centre for Global Health Research (CGHR), Kisian, Kenya
3. College of Health Sciences, Makerere University, Kampala Uganda
4. Hoima Regional Referral Hospital, Hoima, Uganda
5. Jinja Regional referral hospital, Uganda
6. Tororo Hospital, Tororo, Uganda
7. Migori County Referral Hospital, Migori, Kenya
8. Kisumu County Referral Hospital, Kisumu, Kenya
9. Division of Parasitic Diseases and Malaria, US Center for Disease Control and Prevention (CDC), Atlanta, GA, USA
10. National Malaria Control Program, Ministry of Health Kenya, Nairobi, Kenya
11. Ministry of Health, Siaya County, Siaya, Kenya
12. College of Medicine, University of Malawi, Blantyre, Malawi
13. Centre for International Health, &Department of Global Public Health and Primary Care, University of Bergen, Bergen, Norway
14. Ryan White Center for Pediatric Infectious Disease and Global Health, Indiana University School of Medicine, Indianapolis, IN, USA
15. Division of Infectious Diseases, School of Medicine, University of North Carolina, Chapel Hill, USA
16. Department of Medicine Division of Transfusion Medicine and Program in Bioinformatics and Integrative Biology, University of Massachusetts School of Medicine, Worcester, USA

Introduction to long term storage of samples

In addition to the study procedures that have been explained to you already we request to store the small samples of blood that has been obtained from your child as part of this study. We ask that you read this form and ask any questions you may have before you decide whether you agree.

What will happen to the stored samples?

The samples taken at the start of the study (1 sample), at the end (1 sample), and at any time your child is ill during follow-up will be frozen and stored for an unknown period of time for future research studies. The sample will be stored at Kisumu KEMRI/CDC Centre for Global Health Research, Kenya and/or Makerere University, Kampala, Uganda, and later sent to Laboratories in Holland, Norway, England or America for future analysis. They may also be shared with investigators at other institutions carrying out similar research. They will not be sold or used for any commercial purpose.

Level of identification

The name of your child will not be present on these stored samples; instead they will bear anonymous numbers or codes. The name of your child will not appear in any result sheets or reports. These future test results about diseases or other traits which are passed on in families cannot be reported back to you.

Approval before use

Request and permission will be sought from the investigator's Research Ethics Committees (REC) before any use of these stored samples. The request will spell out clearly the intended use for these stored samples. The REC is a special committee that oversees medical research studies to protect the rights and welfare of the human subject volunteers.

What the stored samples may be used for

1. These blood samples will be used for future anaemia and malaria studies.
2. The tests will include traits (or genes) that may cause lack of blood in children or protect them from malaria, such as sickle cell disease and thalassemia. We will also examine the traits (genes) of the malaria parasites.

Results of any future testing will be presented in publications or other scientific meetings.

Risks

There are minimal risks to your child from having future research done on the stored blood samples, because the name of your child is not on the sample and the results cannot be put into your child's study records.

Benefits

There will be no direct benefit to you or your child from future research on your stored blood samples. However, from studying these samples we may learn more about malaria and anaemia which may benefit other children suffering similar problems in the future.

Freedom to refuse

It is up to you to decide if you want your child's blood to be stored for future studies or not. If you choose for your child's blood to be stored you will also be asked to sign a consent form. If you do not want your child's blood to be stored for future studies, now, or in the future, this will not affect the care you will receive for your child. It also does not affect your child's participation in the main PMC-study or future participation in other studies. You can also change your mind and withdraw your permission to store the sample while your child is in the study. Then these samples will no longer be made available for research and will be destroyed. Even if you withdraw your permission, your child can still continue in the main PMC study and your child will still get the same care as the other children in the study.

Contact for further information [For Kenya]/[for Uganda]

[Kenya]

1. [If you have any questions about this study, or if you want your child to stop being part of the study, please contact Dr Simon Kariuki (Tel: 057 202 29 02) or Dr Titus Kwambai (Tel: 0723 354 238), KEMRI/CDC, P.O. Box 1578, Kisumu. You can also contact any of our study staff at the hospital.
2. If you have any questions about your rights as a study patient, or if you think your child has been injured because of this study, please contact The Secretary, KEMRI Scientific and Ethics Review Unit, Mbagathi Rd. Nairobi, Kenya, PO Box 54840-00200, Nairobi; Telephone numbers: 020 2722541, 020 2713349, or 0722-205901.]

[Uganda]

1. [If you have any questions about this study, or if you want your child to stop being part of the study, please contact Dr Richard Idro or Dr Robert Opoka on 0774 274173 or 0772 996164. You can also contact any of our study staff at the hospital.

If you have any questions about your rights as a study patient, or if you think your child has been injured because of this study, please contact Prof James Tumwine, Chairman of the Makerere University School of Medicine Research and Ethics Committee (SOMREC) on 0414530020.]

16.2.4. Consent statement for long-term storage / future studies (English)

Post-Discharge Malaria Chemoprevention PMC Study

Consent statement for long term storage of blood samples and future studies

Title of Study: Malaria Chemoprevention with monthly treatment with dihydroartemisinin-piperaquine for the post-discharge management of severe anaemia in children aged less than 5 years in Uganda and Kenya: A 3-year, multi-centre, parallel-group, two-arm randomised placebo controlled superiority trial

Participant ID: _________________________ (copy assigned ID from the Pre-enrolment CRF)

<table>
<tr><td rowspan="2">The storage of blood was explained to me and I agree for KEMRI/CDC or Makerere University to store my child's blood sample for at least 15 years for future studies. I also understand and agree that my child's blood samples may be sent to Laboratories in Holland, Norway, England or America for future analysis. I understand that I can change my mind to not have my child's blood sample sent, stored or used for future research. To do this, I may tell [Dr Simon Kariuki or Dr Titus Kwambai of KEMRI/CDC (for Kenya),] /[Dr Richard Idro or Dr Robert Opoka of Makerere University (for Uganda)]</td><td colspan="2">If you agree circle "YES", if you do not agree circle 'NO'</td></tr>
<tr><td>YES</td><td>NO</td></tr>
</table>

	Name	Signature or left thumbprint	Today's date
Parent providing consent for child			
Witness*			
Study staff consenting participant			

*A parent/guardian can sign with witness, or verbally state his/her consent in the presence of a witness who will then sign.

16.2.5. Participant Information Sheet for ECG sub study (English)

Post-Discharge Malaria Chemoprevention
PMC Study

Participant Information Sheet
ECG sub study

Title of Study: ECG sub-study within the main trial of post-discharge Malaria Chemoprevention with monthly treatment with dihydroartemisinin-piperaquine

Investigators

Dr Richard Idro[3]
Dr Robert Opoka[3]
Dr. Aggrey Dhabangi[3]
Dr Tom Ediamu[4]
Dr Sophie Namasopo[5]
Dr Harriet Nambuya[5]
Dr Simon Kariuki[2]
Dr Titus Kwambai[2]
Dr Martina Oneko[2]
Dr Grace M Nalwa[7]
Dr Magdalene Kuria[8]
Dr Aaron Samuels[2,9]
Prof Kamija Phiri[12]
Prof Bjarne Robberstad[13]
Prof Brian Faraghar[1]
Prof Duolao Wang[1]
Prof Chandy John[14]
Prof Jonathan J. Juliano[15]
Prof Jeff Bailey[16]
Prof Feiko ter Kuile[1,2]

Institutions

1. Liverpool School of Tropical Medicine (LSTM), Liverpool, United Kingdom
2. KEMRI Centre for Global Health Research (CGHR), Kisian, Kenya
3. College of Health Sciences, Makerere University, Kampala Uganda
4. Hoima Regional Referral Hospital, Hoima, Uganda
5. Jinja Regional referral hospital, Uganda
6. Tororo Hospital, Tororo, Uganda
7. Migori County Referral Hospital, Migori, Kenya
8. Kisumu County Referral Hospital, Kisumu, Kenya
9. Division of Parasitic Diseases and Malaria, US Center for Disease Control and Prevention (CDC), Atlanta, GA, USA
10. National Malaria Control Program, Ministry of Health Kenya, Nairobi, Kenya
11. Ministry of Health, Siaya County, Siaya, Kenya
12. College of Medicine, University of Malawi, Blantyre, Malawi
13. Centre for International Health, &Department of Global Public Health and Primary Care, University of Bergen, Bergen, Norway
14. Ryan White Center for Pediatric Infectious Disease and Global Health, Indiana University School of Medicine, Indianapolis, IN, USA
15. Division of Infectious Diseases, School of Medicine, University of North Carolina, Chapel Hill, USA
16. Department of Medicine Division of Transfusion Medicine and Program in Bioinformatics and Integrative Biology, University of Massachusetts School of Medicine, Worcester, USA

Introduction

The study drug (DP) being given in this PMC study may cause mild and transient changes in heart-beat. We would like therefore to take a heart test (ECG) to evaluate the heart function of your child on top of the main PMC study procedures that have been explained to you already.

Why have I been chosen?
This ECG study is a small study nested within the bigger PMC- study. Only 66 children can take part. Your child has been chosen because this hospital was picked for this procedure.

What will happen if I want to take part?
The heart test (ECG) will be done in a special room in this Hospital and requires the child to lie flat on the back. The doctor or nurse will attach some small bandages to the arms, legs and chest and attach to an ECG machine that can measure the heartbeat. This procedure takes about 5 minutes and is painless, as the ECG bandages simply touch the skin surface without penetration. The results of the ECG will be read and explained to you immediately. If your child's heart function is found to be abnormal, he/she will be referred to a heart specialist for further assessment and treatment. This may result in delaying or withholding the rest of the study drug.

The schedule of the heart test will be as follows; one exam today before taking the study drug, and a second on the 3rd day, 4-6 hours after taking the 3rd dose. This will also be done on each next drug course at 6 weeks and 10 weeks. Thus the total will be six (6) ECG exams.

At the same time of doing the ECG exam, the doctor will take a small amount of blood (200ul) from your child (finger stick) to check drug blood levels. In total, six (6) finger sticks will be taken. These blood samples will be taken for analysis in a laboratory in Thailand. It is important that we ascertain whether the drug taken is reaching the required drug levels in the blood, as this impacts its effectiveness.

What if I don't want to be in the study?
If you decide not to be in this heart test study, it will neither affect the treatment and care of your child while here at hospital nor affect your participation in the main PMC-study or future participation in other studies. You can change your mind and withdraw from this ECG study at any time, and you do not have to give a reason. Even if you withdraw from the ECG study, you can still continue in the main PMC study. You will still get the same care as the other children in the study.

Contact for further information [For Kenya]/[for Uganda]
[Kenya]

1. [If you have any questions about this study, or if you want your child to stop being part of the study, please contact Dr Simon Kariuki (Tel: 057 202 29 02) or Dr Titus Kwambai (Tel: 0723 354 238), KEMRI/CDC, P.O.Box 1578, Kisumu. You can also contact any of our study staff at the hospital.
2. If you have any questions about your rights as a study patient, or if you think your child has been injured because of this study, please contact The Secretary, KEMRI Scientific and Ethics Review Unit, Mbagathi Rd. Nairobi, Kenya, PO Box 54840-00200, Nairobi; Telephone numbers: 020 2722541, 020 2713349, or 0722-205901.]

[Uganda]

1. [If you have any questions about this study, or if you want your child to stop being part of the study, please contact Dr Richard Idro or Dr Robert Opoka on 0774 274173 or 0772 996164. You can also contact any of our study staff at the hospital.

2. If you have any questions about your rights as a study patient, or if you think your child has been injured because of this study, please contact Prof James Tumwine, Chairman of the Makerere University School of Medicine Research and Ethics Committee (SOMREC) on 0414530020.]

16.2.6. Consent statement for ECG sub study (English)

Post-Discharge Malaria Chemoprevention PMC Study

Consent statement

ECG sub-study

Title of Study: ECG sub-study within the main trial of post-discharge Malaria Chemoprevention with monthly treatment with dihydroartemisinin-piperaquine

Participant ID: ______________________________________ (copy assigned ID from the Pre-enrolment CRF)

<table>
<tr><td rowspan="2">
I have been told about the ECG sub-study.
I understand this involves about 6 heart tests in total, including today and in 2 days, and again 2 ECGs next month and the month after that.
I understand that each heart test can take 5 to 10 minutes.
I understand that sometimes I have to wait for 1 hour before the doctor or nurse is free to make the heart test (ECG).
I understand that they will take a small amount of blood from my child to monitor drug levels at each time a heart test is taken.
I also understand and agree that my child’s blood samples may be sent to Laboratories in Thailand, England or America for analysis.
I understand that I can change my mind to not have my child’s blood sample sent, stored or used for future research. To do this, I may tell Dr Simon Kariuki or Dr Titus Kwambai of KEMRI/CDC (for Kenya), Dr Richard Idro or Dr Robert Opoka of Makerere University (for Uganda)
I have been told that it is up to me if I want to join this ECG study and that I can leave the ECG study any time I want, without consequences for my child to be in the main study
</td><td colspan="2">If you agree circle “YES”, if you do not agree circle ‘NO’</td></tr>
<tr><td>YES</td><td>NO</td></tr>
</table>

	Name	Signature or left thumbprint	Today’s date
Parent providing consent for child			
Witness*			
Study staff consenting participant			

*A parent/guardian can sign with witness, or verbally state his/her consent in the presence of a witness who will then sign.

16.2.7. Participant Information Sheet for main trial (DhoLuo)

Nonro mar PMC mar tiyo gi yath egeng'o malaria bang' nindo e od thieth

Oboke ma otingo weche jachiwre e nonro

Wi nonro: Gengo tuo mar Malaria gi thieth mar dwe ka dwe gi yath malaria ma iluongo ni dihydroartemisinin-piperaquine nerit mar nok mar remo bang nindo e od thieth ni nyithindo mahikgi tin ni 5 e Uganda kod Kenya: higni adek e okange ariyo mar thieth maonge yath maoyier radha radha gi migepe machalre, e girube maopogore

Jononro

Dr Richard Idro[3]
Dr Robert Opoka[3]
Dr. Aggrey Dhabangi[3]
Dr Tom Ediamu[4]
Dr Sophie Namasopo[5]
Dr Harriet Nambuya[5]
Dr Simon Kariuki[2]
Dr Titus Kwambai[2]
Dr Martina Oneko[2]
Dr Grace M Nalwa[7]
Dr Magdalene Kuria[8]
Dr Aaron Samuels[2,9]
Prof Kamija Phiri[12]
Prof Bjarne Robberstad[13]
Prof Brian Faraghar[1]
Prof Duolao Wang[1]
Prof Chandy John[14]
Prof Jonathan J. Juliano[15]
Prof Jeff Bailey[16]
Prof Feiko ter Kuile[1,2]

Kidienje

1. Liverpool School of Tropical Medicine (LSTM), Liverpool, United Kingdom
2. KEMRI Centre for Global Health Research (CGHR), Kisian, Kenya
3. College of Health Sciences, Makerere University, Kampala Uganda
4. Hoima Regional Referral Hospital, Hoima, Uganda
5. Jinja Regional referral hospital, Uganda
6. Tororo Hospital, Tororo, Uganda
7. Migori County Referral Hospital, Migori, Kenya
8. Kisumu County Referral Hospital, Kisumu, Kenya
9. Division of Parasitic Diseases and Malaria, US Center for Disease Control and Prevention (CDC), Atlanta, GA, USA
10. National Malaria Control Program, Ministry of Health Kenya, Nairobi, Kenya
11. Ministry of Health, Siaya County, Siaya, Kenya
12. College of Medicine, University of Malawi, Blantyre, Malawi
13. Centre for International Health, &Department of Global Public Health and Primary Care, University of Bergen, Bergen, Norway
14. Ryan White Center for Pediatric Infectious Disease and Global Health, Indiana University School of Medicine, Indianapolis, IN, USA
15. Division of Infectious Diseases, School of Medicine, University of North Carolina, Chapel Hill, USA
16. Department of Medicine Division of Transfusion Medicine and Program in Bioinformatics and Integrative Biology, University of Massachusetts School of Medicine, Worcester, USA

Gima omio itimo nonro
KEMRI/CDC kod mbalariany ma Makerere tiyo ka oriwore gi migao mar thieth ma Kenya kod Uganda e timo nonro mar yudo yore makare mar duoko chien chandruok mar thoe kod dok kendo nindo eod thieth nikech nok mar remo (bedo maonge gi remo ahinya).

Nonro en mar ang'o?
Nyithindo maodak kuonde ma tuo mar malaria ngeny mothieth ni nok mar remo ni okang' mamalo mar bedo matuo kendo kata tho e thuolo mar dweche adek bang' wuok e od thieth.

Nonroni dwaro yudo yo makare mainyalo geng'go tho kata dok kendo nindo e od thieth ni nyithindo maose yudo thieth mar nok mar remo. Wadwaro ng'eyo ka mwonyo yath manyien mailwongo ni DHA-piperaquine (DP) e jumbe 2,6,kod 10 bang wuok e od thieth nyalo ber moloyo coartem kende bang' wuok e od thieth.

Ang'o maomiyo nyathini oyier?
Nyathini ne onindo e od thieth nikech ne otuo ahinya nikech nok mar remo. Ng'eny nyithindo mantie ute thieth nikech tuoni ibiro kwa mondo obed jokanyo e nonro ni. Wabiro kwayo nyithindo 2212 ma higni ni piny mar higni 5 mondo obed jochiwre e nonro ni.

Ang'o mabiro timo nyathina kaoyie bedo jachiwre e nonro ni?
Ka iyie bedo jachiwre e nonroni, wabiro penji penjo moko ewi tuo nyathini kod kit thieth mane oyudo kapok nende ilimo od thieth kod chudo te.Wabiro penji bende kama iaye, seche mag wuoth kod kar chudo maitiyogo mondo ichop e od thieth, kit gedo mar odi , sombi kod, yo maduong' ma jo ot yutogo.

Jarit ngima/laktar mar jo nonro biro neno/pimo nyathini kendo bende wabiro kawo weche ewi tuo nyathini e andike mag od thieth kod kar pimo remo.

Ang'o mabotimore ni remo mokaw ei nonroni?

Wabiro golo kendo remo matin (kijiko achiel mar chae) e ler mar bad nyathini. Remono ibiro tigo e pimo malaria kod nok mar remo. Inyalo tim pimni ei od thieth to wanyalo nyisi duoko mar pimno.

E higni/kinde mabiro, wabiro ngiyo kido manyalo geng'o gi kuom yudo tuo mar malaria kod makelo nok mar remo kaka sickle cell kod thalassemia. Wabiro pimo remogo mondo wangi ka yedhe mag malaria pod tiyo. Bende wabiro pimo ka kido mar kute mag malaria olokre gi kinde.

Thieth mar nonro chal nade?
Nikech nonroni, wabiro miyo nyithindo tee coartem kagisebedo mangima manyalo chiemo kendo, kata ka gionge malaria ndalo maneginido e od thieth. Jarit ngima mar nonro biro miyo nyathini yath maokwongo e od thieth ka. Ka nyathini osebedo mangima manyalo wuok e od thieth, nyaka omwuny yien modong kuom ndalo adek nyaka yien rum.

Ang'o mabiro timre ka asewuok e od thieth?
Wabiro kwayi mondo idwog e od thieth bang jumbe ariyo mondo oneni kendo. E thuolono, wabiro keto nyathini e thieth mar nonro.

Ere kaka gibiro ng'ado yor thieth manyathina biro yudo?

Nitiere yore ariyo mag thieth, DP kod yath machal DP(yath machal kod DP to onge kod yath kuome). Nyathini ibiro yier mondo oyud acheil kuomgi. Yiero mar kit thieth ma nyathini biro yudo ok ochan. Kit thieth ariyogi chalre ma kata jarit ngima mabiro thiedho nyathini ok ong'eyo ni mane kuom kit thieth ariyogi ma nyathini biro yudo.

Yedhe nonro imwonyo nadi?
Thieth ibiro chiw diechiel e odioching' kuom ndalo adek. Jarit ngima mar nonro biro miyo nyathini yethe maokwongo mag thieth e od thieth kae kata e dalani. Nyithindo te e kidienjegi biro yudo thieth mar yedhe maoregi maoketi e yath mimadho. Dose ariyo maodong ibiro mwony e dala. Jarit ngima mar nonro biro paroni e ong'we yamo mondo imi nyathi yath kadwarore.

Be nitie gimachielo maubiro timo ekinde limbe mar jumbe ariyo?
Wabiro kawo remo matin e ombong tiend nyathini kata e lith lwete mondo wapim nok mar remo. Bende wabiro miyo nyathini yedhe remo kaka yo mar thiedho nok mar remo, nyaka omwonygi pile kuom dwe achiel.

Nyaka adwok nyathina kendo?
Eee, wabiro dwaro neno nyathini bang' jumbe auchiel kendo bang jumbe apar mondo omiye thieth mar nonro kaka oyang malo kacha.**Bende wabiro gochoni e dwe mari mar ang'wen gi abich mondo wang'eane kaka nyathini dhi to bende waparni limbe mogik mar klinik mari mogik mar dweche auchiel (jumbe piero ariyo gi auchiel)**

Limbe mar dweche auchiel
Wabiro dwaro neno nyathini dichiel mogik dweche auchiel bang' kawuono mondo wang'e ka yedhe osetiyo maber. E thuolono , wabiro penji penjo ewi ngima nyathini, nono kod pimo ratind nyathini kod pimo liet mar dende. Wabiro kawo remo martin e lith lwet nyathini (matin ni nus kijiko mar chae). Remono ibiro pimgo malaria kod kaka remo rom.

Ang'o manyaka atim?
Kaiyie bedo jachiwre e nonro nyaka bed ni inyalo yudori kuom thuolo mar dweche auchiel kochakore chieng' maidonjoe nonro.

En kare mondo nyathini omwony yedhe duto mag nonro. Kik ipog yedhegi ni nyithindi mamoko kata gibende gin e nonro.

Nyithindo maodonjo e nonro nyalo mana mwonyo kit yedhe moko. Kuom ma wakwayi mondo kik inyiew yedho moko ni nyathini e thuolo maen e nonro ei duka. Ka nyathini tuo e kinde moro a mora e thuolo mar dweche auchiel mar nonro, inyalo kelo nyathini kar thieth mar nonro e od thieth ka. Wabiro kawo ting mar yor wuoth ([siling mia abich]) [Uganda the amount will be left blank per local regulations] mar biro e od thieth gi dok. [Kenya: Wabiro kawo ting ei yor nindo e od thieth kod chiembi gi chiemb nyathini kaponi inindo e od thieth. [Uganda: ibiro miyi pesa kinde mibiro e limbe kuom seche ni, gi chiemo]. Bende wabiro chulo pesa e touche maluowore gi bedo ei nonroni kod pesa maibiro dwar kinindo e od thieth kaluwore gi nonroni.

Chandruoge mage ma yedho nonro kelo?
Yedhe duto nigi chandruoge magikelo. Nyathini nyalo ng'ok bang muonyo yedhe gi. .Ng'ok jabedoga mang'eny ahinya to ka nyathi nikod del maowore. Ka nyathi ong'ok e thuolo mar nyirri 30, yedhe moko mag thieth ibiro miye. Ka ng'ok obedoe e bang' mar nyirri 30 nus mar yedhe ibiro nwo miye. Ka ng'ok obedoe saa achiel, onge yedhe madwarore. Kiyie tim ler ni jarit ngima mar nonro maoiyo

kaka nyalre ka nyathi ong'ogo yedhe. Ka nyathi osemuony yath nonro wabokwayi mondo ibedo ei od thieth kuom saa achiel.

Kuom duto tee nyithindo dhi ga maber kod yedhe nonro. Kuom ndalo matin nyithindo ywago wich bar,wang'malil kata chuny malem kod ich maremo. Kuom nyithindo mang'eny mae bedoga matin. Ng'eny ranyisigi nyaloga bedoe nikech malaria.

DP nyalo kelo chandruok matin e kaka adundo gwecho. Wabiro timo rit/ngiyo ka nyathini ni kod chandruok mar kor maremo kata adundo magwecho matek. Wabiro timo mae kawapimo adundo (ECG).

Chandruoge mage mantie e chiwruok bet e nonroni?

Kaiyie bet e nonroni, chundruoge tin. Adhola matin kata rem matin nyalo betie bat maogolie remo. Thuolo bende nitie ni chandruok nyalo bet e kama ogolie remo. Thuoloni tin ahinya nikech watiyoga kod gik tich maler. Nonroni biro dwaro mondo ilim od thieth mang'eny moloyo mapile. Mae nyalo miyo iyud pek. Wabiro dwokoni chudo ma itiyogo e yor wuoth.

Be nitie ber moro a mora ni nyathina?

Onge ber ma nyathini biro yudo en owuon. Kata kamano wabiro kawo ting mar chudo mar thieth ni nyathini kuom dweche auchiel maobiro betie nonro. Mae ok oriwo yeng'o kod masira, kata tuo moro amora maokobedoe nikech bedo jachiwre e nonro makmana ka ma otudore gi nonro.Nikech nyathini biro limo od thieth maluwore e kinde ma nonro dhi nyime wabironyisi kawayudo tuo moro a mora manyalo kelo chandruok ni ngima nyathini kata thiethe mapiyo.

To kapo ni gimoro odhi marach?

Nyathini biro yudo thieth manono ni hinyruok kata tuo maobedoe nikech bedo jachiwre e nonro. E thuolo ma nonro dhi nyime, wabironyisi kawayudo tuo moro a mora manyalo kelo chandruok ni ngima nyathini.

Bende ibiro kan maling ling bedona jachiwre e nonroni?

Ka iyie bet e ononroni wabiro kano nying nyathini kod weche duto mawayudo kuomi kaluwore gi nonro maopondo kaluwore kod kaka chik dwaro. Mana jononro kod jotij sirikal moyang emanyalo neno wecho maondikie nyingi kod nying nyathini. Wabiro tiyo kod weche ma imiyowa e nonro kende. Weche ma ochoki inyalo tigo gi jomamoko mawuok e migepe kod pinje mamoko bende kata kamano nying nyathini ok bi ket e riport/dwoko moro a mora.

Okawo thuolo marom nadi?

Nyathini bobedo e nonro kuom deche auchiel chakre kawuono. Ibiro limi bang jumbe ariyo (2), auchiel (6), apar (10) kod giko mar dwe mar auchiel (6) kaka oseler malo kanyo. Limbe maluwe biro kawo kind dakika 60-90.

Ochuno ni nyathina nyaka bed jachiwre?

In thuolo mar yie ka idwaro mondo nyathini obed e nonro kata da. Kaiyie mondo nyathini obed e nonro ibiro kwayi mondo iket lweti e oboke mar ayie. Ibiro bedo thuolo mar weyo saa a saya maidwaro maok ichiwo pachi. Kaok adwar mondo nyathini o bed e nonro sani kat kinde mabiro,mae ok bi ketho ratiro mare mar yudo rit kaka owinjore.

Jok mainyalo tudrigo

[Kenya]

1. [Kain kod penjo moro a mora e wi nonroni, yie itudori kod Dr Simon Kariuki, KEMRI/CDC, P. O. Box 1578, Kisumu. Inyalo gochone e namba mar sim (Tel: 057 202 29 02) kata Dr Titus Kwambai (namba sim: 0723 354 238), KEMRI/CDC, P.O.Box 1578, Kisumu. KEMRI/CDC, P. O. Box 1578, Kisumu. Bende inyalo wuoyo gi jotich nonri e od thieth
2. Kain kod penjo kaluwore kod ratiro mari kaka jachiwre e nonro,kata nyathini ohinore nkech nonroni yie itudoro kod The Secretary, KEMRI Scientific and Ethics Review Unit, Mbagathi Rd. Nairobi, Kenya, PO Box 54840-00200, Nairobi; namba sim: 020 2722541,020 2713349, or 0722-205901.]

[Uganda]

1. [Kain gi penjo moro amora kaluowore gi nonroni kata idwaro ni nyathini owe noroni, yie itudri gi Dr Richard Idro or Dr Robert Opoka on 0774 274173 or 0772996164. Bende inyalo tudri gi jotich nonro e od thieth.
2. Kain kod penjo kaluwore kod ratiro mari kaka jachiwre e nonro,kata nyathini ohinore nkech nonroni yie itudoro kod Prof James Tumwine, Chairman of the Makerere University School of Medicine Research and Ethics Committee (SOMREC) on 0414530020.]

Wageno ni duoko wamayudo e nonroni bokele lokruok e thieth mar nok mar remo karu kae.

Ero kamano kuom secheni.

Ibiro miyi achiel kuom oboke mar weche mag nonro kod oboke mogo seyi mondo ikan. Oboke machielo ibokan gi jotij nonro.

16.2.8. Consent statement for main trial (DhoLuo)

Nonro mar PMC mar tiyo gi yath egeng’o malaria bang’ nindo e od thieth

Weche mane obokemar chiwo ayie

Wi nonro: Gengo tuo mar Malaria gi thieth mar dwe ka dwe gi yath malaria ma iluongo ni dihydroartemisinin-piperaquine nerit mar nok mar remo bang nindo e od thieth ni nyithindo mahikgi tin ni 5 e Uganda kod Kenya: higni adek e okange ariyo mar thieth maonge yath maoyier radha radha gi migepe machalre, e girube maopogore**.**

(Namba kata ranyisi mar ng’ama nitiere e norno) ________________________________ (Gol ranyisi mar ng’ama nitiere norno e Pre-enrolment CRF)

Osepimna kendo ayie mondo nyathina obed jachiwre enonro ni. Awinjo ni an thuolo mar yiero mondo nyathina obed e nonro kendo tamruok ok bi kelo lokruok ni nyathina. Ayie mondo onona gi jotich nonroni kendo ayie mondo ogol remb nyathina matin e lith lwete/ombong tielo mondo otigo e pimo malaria kod nok mar remo kod kido manyalo kelo nok remo kata geng’o malaria. Awinjo ni nyathina bobedo e nonro kuom dweche auchiel. Ayie kelo nyathina mondo onone kata mondo jotij nonroni olima e dala e jumbe mar ariyo (2), auchiel (6) kod apar (10) kendo bang dwe mar auchiel (6). Bende ayie kelo nyathina e od thieth kaka dwarore kata mondo jotij nonroni olima e dala ,**kata tudore koda e yor simu.** Awinjo ni kidienje ma otudore kod weche mag ngima nyathina inyalo ndiki kod adier [facts] ma ochok e nonroni inyalo ng’i kod jotich mowuok KEMRI/CDC kod mbalariany mar Makerere (kar tiegruok). Achiwo thuolo mondo jogo one andike go (records) kendo opimgi kod mar kuonde nonro mamoko. Ayie bende ni adier maochok ka owuok e nonro ni otigo gi migepe mag nonro mamoko mowuok oko mar Kenya gi Uganda maonge gi nying nyathina kod nying kama odakie.	Kaiyie lwor “Eee”, kaok iyie agree lwor “ooyo”.	
	EEE	OOYO

	Nying	seyi **kata lith lwedo maduong makoracham**	Tarik makawuono
Janyuol machiwo yie ni nyathine.			
Janeno*			
Jatij nonro machiwo ayie			

*Janyuol kata jarit nyalo keto seyi gi janeno, kata wach yie mare ka janeno ma bang’e biro keto seyi nitie.

16.2.9. Participant Information Sheet long-term storage / future studies (Dholuo)

Post-Discharge Malaria Chemoprevention PMC Study

Yie mar kano remo ni nonro mar kinde mabiro oboke maoting'o weche kano remo ni nonro mag higni makinde

Wi nonro: Gengo tuo mar Malaria gi thieth mar dwe ka dwe gi yath malaria ma iluongo ni dihydroartemisinin-piperaquine nerit mar nok mar remo bang nindo e od thieth ni nyithindo mahikgi tin ni 5 e Uganda kod Kenya: higni adek e okange ariyo mar thieth maonge yath maoyier radha radha gi migepe machalre, e girube maopogore*.*

Jononro

Dr Richard Idro[3], *Dr Harriet Nambuya[5]*, *Dr Magdalene Kuria[8]*, *Prof Duolao Wang[1]*
Dr Robert Opoka[3], *Dr Simon Kariuki[2]*, *Dr Aaron Samuels[2,9]*, *Prof Chandy John[14]*
Dr. Aggrey Dhabangi[3], *Dr Titus Kwambai[2]*, *Prof Kamija Phiri[12]*, *Prof Jonathan J. Juliano[15]*
Dr Tom Ediamu[4], *Dr Martina Oneko[2]*, *Prof Bjarne Robberstad[13]*, *Prof Jeff Bailey[16]*
Dr Sophie Namasopo[5], *Dr Grace M Nalwa[7]*, *Prof Brian Faraghar[1]*, *Prof Feiko ter Kuile[1,2]*

Kidienje

1. Liverpool School of Tropical Medicine (LSTM), Liverpool, United Kingdom
2. KEMRI Centre for Global Health Research (CGHR), Kisian, Kenya
3. College of Health Sciences, Makerere University, Kampala Uganda
4. Hoima Regional Referral Hospital, Hoima, Uganda
5. Jinja Regional referral hospital, Uganda
6. Tororo Hospital, Tororo, Uganda
7. Migori County Referral Hospital, Migori, Kenya
8. Kisumu County Referral Hospital, Kisumu, Kenya
9. Division of Parasitic Diseases and Malaria, US Centers for Disease Control and Prevention (CDC), Atlanta, GA, USA
10. National Malaria Control Program, Ministry of Health Kenya, Nairobi, Kenya
11. Ministry of Health, Siaya County, Siaya, Kenya
12. College of Medicine, University of Malawi, Blantyre, Malawi
13. Centre for International Health, & Department of Global Public Health and Primary Care, University of Bergen, Bergen, Norway
14. Ryan White Center for Pediatric Infectious Disease and Global Health, Indiana University School of Medicine, Indianapolis, IN, USA
15. Division of Infectious Diseases, School of Medicine, University of North Carolina, Chapel Hill, USA
16. Department of Medicine Division of Transfusion Medicine and Program in Bioinformatics and Integrative Biology, University of Massachusetts School of Medicine, Worcester, USA

Lero mokuongo mar keno mabudho mar gige pim

Komedore gi chenro mar nonro ma ose lerni ka, wakwayo ni wabiro kano remo matin mar pim mawa golo kuom nyathini kaka achiel mar nonro ni. Wakwayo ni isom obokeni kato ipenj penjo kapok ing'ado ka iyie

Ang'oma biro timorene gi pim ma okan

Gige pim ma okaw ka ochak nonro gi pim achiel to kendo giko nonro gi pim achiel kendo gi saa moro amora ma nyathi tuo kendo ndalo limbe ibiro ket mang'ich kendo ikano kuom ndalo maokongere kuom nonro makinde mabiro. Remo ibokan Kisumu KEMRI/CDC kar nonro mar piny ma weche thieth man Kenya to gi kar tiegruok ma malo mar Makerere man Uganda kato bang'e ibi orgi kar pim mantie Holland, Norway, England to gi America mar pim mabiro bang'e. Samoro bende inyal rang gi Jononro matimo nonro machak kama emi gepe moko. Ok bi us gi kata tiyo kodgi eyor ohala.

Kaka inyal ng'e

Nying nyathini ok bi bedo gige pim ma okan gi to gi biro bedo gi namba mopondo. Nying nyathini ok bi bedo eduoko moro amora kata ripot. Pim ma ibiro tim bang'e gi kuom tuoché gi kata kido ma iyudo koa kuom anyuola to ok bi miyi duoko

Puodho kapok otiyo

Ibiro kwa thuolo from Jatend nonro maduong'(Riwruok mar joma rango weche nonro) kapok oti gi gige pimgi mokan. Kwayono biro wacho malee gima ichan tim gi gige pim mokan gi. REC en riwruok makende mang'iyo weche nonro mag thieth mondo okony ratiro mag joma ochiwore enonro

Gima inyalo tim gi gige pim mokan

1. Gige pim mag remo ibi ti godo enonro mabiro mag tuo midusi gi tuo remo
2. Pim go biro rango ganyisi manyalo kelo onge mar remo enyithindo kata geng'o ne gi midusi, sickle cell to gi thalassemia.Wabiro kendo rango ranyisi mag malaria

Duoko mag pim mabiro ibi kete eboke kata buche mag Sayans.

Rach mag nonro

Onge rach wabiro bedo ne nyathini koa kuom timo nonro gi gige pimma ibiro kan nikech nying nyathini ok bi bedo egige pim go kendo duoko ok bi keti ericods mag nyathini

Ber mar nonro

Onge ber mabiro bedoni kata nyathini koa kuom nonro mibiro tim gi gige pim mokan gi. To kata kamano nonro mibiro tim ne gige pim gi biro wabiro ng'eyo mang'eny kuom tuo mar midusi gi tin mar remo manyalo konyo nyithindo ma touché gi chando e ndalo mabiro

Yiero mar tamruok

En yiero mari mondo iyie kata tamri mondo remb nyathini okan mar timo nonro endalo mabiro.Kiyie mondo remb nyathini okan to ibi kwayi mondo iket lueti eboke mar andika.ka ok iduar ni gige pim mar nyathini kik kan sani kata ndalo mabiroto ok bi mono nyathini yudo thieth mapile.bende ok obi mono nyathini bedo enonro maduong mar PMC kata nonro mabiro. Bende inyalo loko pachi kanyathini nie nonro, kendo gige pim gi ok bi ti godo enonro to ibikethgi. To kata ka igolo yie mar nyathini to pod onyalo bedo enonro maduong' mar PMC kendo nyathini biro yudo rit man aka nyithindo moko mantie enonro.

Tudruok ka in gi wach

[Kenya]

1. [ka in gi penjo moro amora kuom nonro ni, kata ka idwaro ni nyathini owe bedo enonro , yie itudri gi Dr Simon Kariuki (Tel: 057 202 29 02) or Dr Titus Kwambai (Tel: 0723 354 238), KEMRI/CDC, P.O.Box 1578, Kisumu. Kendo inyalo tudri gi jononro matiyo hospital.
2. Ka in gi penjo moro amora kuom ratiro marikata ka iparo ni nyathini ohiny nikech nonro, yie itudri The Secretary, KEMRI Scientific and Ethics Review Unit, Mbagathi Rd. Nairobi, Kenya, PO Box 54840-00200, Nairobi; Telephone numbers: 020 2722541, 020 2713349, or 0722-205901.]

[Uganda]

1. [ka in gi penjo moro amora kuom nonro ni, kata ka idwaro ni nyathini owe bedo enonro , yie itudri gi Dr Richard Idro or Dr Robert Opoka on 0774 274173 or 0772 996164. Kendo inyalo tudri gi jononro matiyo hospital.
2. Ka in gi penjo moro amora kuom ratiro marikata ka iparo ni nyathini ohiny nikech nonro, yie itudri Prof James Tumwine, Chairman of the Makerere University School of Medicine Research and Ethics Committee (SOMREC) on 0414530020.]

16.2.10. Consent statement long-term storage / future studies (Dholuo)

Nonro mar PMC mar tiyo gi yath egeng'o malaria bang' nindo e od thieth.

Yie mar kano remo ni nonro mar kinde mabiro

Wi nonro: Gengo tuo mar Malaria gi thieth mar dwe ka dwe gi yath malaria ma iluongo ni dihydroartemisinin-piperaquine nerit mar nok mar remo bang nindo e od thieth ni nyithindo mahikgi tin ni 5 e Uganda kod Kenya: hign adek e okange ariyo mar thieth maonge yath maoyier radha radhagi migepe machalre, e girube maopogore.

Namba kata ranyisi mar ng'ama nitiere e norno)________________________________ (Gol ranyisi mar ng'ama nitiere norno e Pre-enrolment CRF)

<table>
<tr><td rowspan="2">Kano remo ne olerna maber kendo ayie ni KEMRI/CDC kata Makerere University mondo okan gige pim mar remo mar nyathina kuom katin to higni apar gi abich kuom nonro mabiro.kendo ase winjo kendo ayie ni remb nyathina mondo oter kar pim ma Holland, Norway, England kata America mondo otim go pim mabiro. Ang'eyo ni anyalo loko pacha mondo kik remb nyathina okan kata oter oko mondo otim go nonro mabiro. Mondo atim ma anyalo nyiso[Dr Simon Kariuki or Dr Titus Kwambai of KEMRI/CDC (for Kenya),] /[Dr Richard Idro or Dr Robert Opoka of Makerere University (for Uganda)]</td><td colspan="2">Kaiyie lwor Eee, kaok iyie agree lwor ooyo.</td></tr>
<tr><td>EEE</td><td>OOYO</td></tr>
</table>

	Nying	Seyi **kata lith lwedo maduong makoracham**	Tarik makawuono
Janyuol mahiwo yie ni nyathi.			
Janeno*			
Janonro machiwo ayie			

*Janyuol kata jarit nyalo keto seyi gi janeno,kata wach yie mare ka janeno ma bang'e biro keto seyi nitie.

16.2.11. Participant Information Sheet for ECG sub study (Dholuo)

Nonro mar PMC mar tiyo gi yath egeng'o malaria bang' nindo e od thieth.

Oboke mar weche mag pim mag adundo e kidienje mar nonroni

Wi nonro: Pim mag adundo e kidienje mar nonro mar PMC mar tiyo gi yath egeng'o malaria bang' nindo e od thieth

Jononro

Dr Richard Idro[3] *Dr Harriet Nambuya[5]* *Dr Magdalene Kuria[8]* *Prof Duolao Wang[1]*
Dr Robert Opoka[3] *Dr Simon Kariuki[2]* *Dr Aaron Samuels[2,9]* *Prof Chandy John[14]*
Dr. Aggrey Dhabangi[3] *Dr Titus Kwambai[2]* *Prof Kamija Phiri[12]* *Prof Jonathan J. Juliano[15]*
Dr Tom Ediamu[4] *Dr Martina Oneko[2]* *Prof Bjarne Robberstad[13]* *Prof Jeff Bailey[16]*
Dr Sophie Namasopo[5] *Dr Grace M Nalwa[7]* *Prof Brian Faraghar[1]* *Prof Feiko ter Kuile[1,2]*

Kidienje

1. Liverpool School of Tropical Medicine (LSTM), Liverpool, United Kingdom
2. KEMRI Centre for Global Health Research (CGHR), Kisian, Kenya
3. College of Health Sciences, Makerere University, Kampala Uganda
4. Hoima Regional Referral Hospital, Hoima, Uganda
5. Jinja Regional referral hospital, Uganda
6. Tororo Hospital, Tororo, Uganda
7. Migori County Referral Hospital, Migori, Kenya
8. Kisumu County Referral Hospital, Kisumu, Kenya
9. Division of Parasitic Diseases and Malaria, US Center for Disease Control and Prevention (CDC), Atlanta, GA, USA
10. National Malaria Control Program, Ministry of Health Kenya, Nairobi, Kenya
11. Ministry of Health, Siaya County, Siaya, Kenya
12. College of Medicine, University of Malawi, Blantyre, Malawi
13. Centre for International Health, &Department of Global Public Health and Primary Care, University of Bergen, Bergen, Norway
14. Ryan White Center for Pediatric Infectious Disease and Global Health, Indiana University School of Medicine, Indianapolis, IN, USA
15. Division of Infectious Diseases, School of Medicine, University of North Carolina, Chapel Hill, USA
16. Department of Medicine Division of Transfusion Medicine and Program in Bioinformatics and Integrative Biology, University of Massachusetts School of Medicine, Worcester, USA

Lero mokuongo

Yath ma (DP) ma ichiwo enonro mar PMC nyalo kelo lokruok matin kaka adundo gocho. Wanyalo hero kawo pim mar adundo(ECG) mondo wang'e kaka adundo tiyo mar nyathini ka iweyo weche ma oselerni kuom nonro mar PMC

Ang'oma omiyo oyiera?
Nonro ma iluongo ni ECG en nonro matin ma oten kuom nonro maduong mar PMC. nyithindo madirom 65 nyalo ndonje enonro. Nyathini oyier nikech hospitandni ne okaw mondo otimie chenro mar pimni

Ang'oma biro timore ka adwaro donje enonro?
Pim mar ECG ibiro tim eot makende ei hospital ka kendo ibiro dwar ni nyathi onind piny ka oriere gi ng'eye. Daktari kata sista biro keto bandej, eluedo,kor to gi tielo to omako gi machine mar ECG manyalo pimo kaka adundo gocho. Chenro ni kawo madirom dakika abich kendo ok orem nikech bandages mako del gi oko maok donj oko. Duoko mar ECG ibiro som kato inyisi mapiyo. Ka adundo nyathini oyudi ni ok goch maber to ibiro ore ir Daktari mobuodhi eweche mag adundo mondo omed range gi dhiedhe. Manyalo kelo deko kata wweyo miye yedhe mag nonro moko

Chenro mar pimo adundo biro chalo kama, pim mokuongo kawuono kapok omuonyo yadh nonro, mar ariyo odiechieng' mar adek,ekind seche 4-6 bang' kawo yath mar adek. Ma biro timore elimbe mar wige 6 to gi apar. Koro koriwore to biro bedo pim auchiel(6) mar ECG

Esama itime pim mar ECG daktari biro kawo remo matin madirom (200ul) koa kuom luet nyathini mondo orang godo kar romb remo mar nyathini. Koriwore to ibi kaw remo elith lwedo diuchiel. Remo mar pim ni ibi ter kar pim ma Thailand. Bende ber kawang'eyo ni yath ma omuony chopo kaka owinjore eremo nikech makonyo tich mar nyadhni.

Ka ok adwar bedo enonro
Ka iyiero mondo kik ibed enonro mar pim mar adundo, ok bimono nyathini yudo thieth kata rich ka en e hospital ka kata moni bede enonro mar PMC kata bedoe enonro mabiro . Inyalo loko pachi ma iwuok enonro marECG saa asaya ma idwaro kata ok iwacho gima omiyo. Kata ka iwuok enonro mar ECG to pod inyalo dhi nyime gi bedo enonro mar PMC. Pod ibiro yudo rit makare mana ka nyithindo man enonro.

Tudruok ka in wach
[Kenya]
1. [ka in gi penjo moro amora kuom nonro ni, kata ka idwaro ni nyathini owe bedo enonro , yie itudri gi Dr Simon Kariuki (Tel: 057 202 29 02) or Dr Titus Kwambai (Tel: 0723 354 238), KEMRI/CDC, P.O.Box 1578, Kisumu. Kendo inyalo tudri gi jononro matiyo hospital.
2. Ka in gi penjo moro amora kuom ratiro marikata ka iparo ni nyathini ohiny nikech nonro, yie itudri The Secretary, KEMRI Scientific and Ethics Review Unit, Mbagathi Rd. Nairobi, Kenya, PO Box 54840-00200, Nairobi; Telephone numbers: 020 2722541, 020 2713349, or 0722-205901.]

[Uganda]
1. [ka in gi penjo moro amora kuom nonro ni, kata ka idwaro ni nyathini owe bedo enonro , yie itudri gi Dr Richard Idro or Dr Robert Opoka on 0774 274173 or 0772 996164. Kendo inyalo tudri gi jononro matiyo hospital.
2. Ka in gi penjo moro amora kuom ratiro marikata ka iparo ni nyathini ohiny nikech nonro, yie itudri Prof James Tumwine, Chairman of the Makerere University School of Medicine Research and Ethics Committee (SOMREC) on 0414530020.]

16.2.12. Consent statement for ECG sub study (Dholuo)

Nonro mar PMC mar tiyo gi yath egeng'o malaria bang' nindo e od thieth

Oboke mar ayie mag pim mag adundo e kidienje mar nonroni

Wi nonro: Pim mag adundo e kidienje mar nonro mar PMC mar tiyo gi yath egeng'o malaria bang' nindo e od thieth

(Namba kata ranyisi mar ng'ama nitiere e norno) ______________________________ (Gol ranyisi mar ng'ama nitiere norno e Pre-enrolment CRF)

<table>
<tr><td rowspan="2">• Ose nyisa kuom nonro mar ECG .
• Ang'eyo ni ibiro tim pim mar adundo diuchiel ka oriwore gi kawuono kendo bang' ndalo ariyo kendo 2 ECG dwe mabiro kendo dwe maluwo bang' mano.
• Ang'eyo ni pim mar adundo moro ka mora nyalo kawo ekind dakika abich nyaka apar.
• Ang'eyo ni samoro nyaka ariti kuom saa achiel kapok daktari kata sista obedo gi thuolo mar pimo adundo (ECG).
• Ang'eyo ni ibi kaw remo matin kuom nyathina mondo opim godo teko mar yath saa asaya mopim adundo
• Ang'eyo kendo ayie ni gipim mar remo mar nyathina inyal ter kar pim ma Thailand, England or America mondo otimne pim mamoko.
• Ang'eyo ni anyalo loko pacha mondo kik remb nyathinal kata kan ne nonro mabiro. Mondo atim ma abiro nyiso Dr. Simon Kariuki kata Dr Titus Kwambai ma KEMRI/CDC (ma Kenya), Dr Richard Idro ma Dr Robert Opoka of Makerere University (ma Uganda)
• Ose nyisa ni en hero mara mondo adonj enonro ECG kendo anyalo weyo nonroni ECG saa asaya maduaro maonge gima nyalo timo nyathina enonro maduong</td><td colspan="2">Kaiyie lwor" Eee", kaok iyie agree lwor "ooyo".</td></tr>
<tr><td>EEE</td><td>OOYO</td></tr>
</table>

	Nying	Seyi **kata lith lwedo maduong** makoracham	Tarik
Janyuol mahiwo yie ni nyathi.			
Janeno*			
Janonro machiwo ayie			

*Janyuol kata jarit nyalo keto seyi gi janeno, kata wach yie mare ka janeno ma bang'e biro keto seyi nitie.

16.2.13. Participant Information Sheet for main trial (Kiswahili)

Uchunguzi wa PMC ya kuzuia malaria kwa kutumia dawa baada ya kuondoko hospitalini

Fomu ya maelezo ya mshiriki ya utafiti

Kichwa: Kuzuia malaria kwa dawa na matibabu ya kila mwezi kwa kutumia dihydroartemisinin-piperaquine kwa udhibiti wa makali na upungufu wa damu kwa watoto chini ya miaka mitano walio ruhusiwa kurudi nyumbani nchini Uganda na Kenya: Majaribio ya miaka mitatu ya udhibiti ubora wa kipozaungo bila mpangilio kwa viungo viwili, kundi sambamba, vituo mabalimbali.

Wachunguzi

Dr Richard Idro[3]
Dr Robert Opoka[3]
Dr. Aggrey Dhabangi[3]
Dr Tom Ediamu[4]
Dr Sophie Namasopo[5]
Dr Harriet Nambuya[5]
Dr Simon Kariuki[2]
Dr Titus Kwambai[2]
Dr Martina Oneko[2]
Dr Grace M Nalwa[7]
Dr Magdalene Kuria[8]
Dr Aaron Samuels[2,9]
Prof Kamija Phiri[12]
Prof Bjarne Robberstad[13]
Prof Brian Faraghar[1]
Prof Duolao Wang[1]
Prof Chandy John[14]
Prof Jonathan J. Juliano[15]
Prof Jeff Bailey[16]
Prof Feiko ter Kuile[1,2]

Taasisi.

1. Liverpool School of Tropical Medicine (LSTM), Liverpool, United Kingdom
2. KEMRI Centre for Global Health Research (CGHR), Kisian, Kenya
3. College of Health Sciences, Makerere University, Kampala Uganda
4. Hoima Regional Referral Hospital, Hoima, Uganda
5. Jinja Regional referral hospital, Uganda
6. Tororo Hospital, Tororo, Uganda
7. Migori County Referral Hospital, Migori, Kenya
8. Kisumu County Referral Hospital, Kisumu, Kenya
9. Division of Parasitic Diseases and Malaria, US Center for Disease Control and Prevention (CDC), Atlanta, GA, USA
10. National Malaria Control Program, Ministry of Health Kenya, Nairobi, Kenya
11. Ministry of Health, Siaya County, Siaya, Kenya
12. College of Medicine, University of Malawi, Blantyre, Malawi
13. Centre for International Health, &Department of Global Public Health and Primary Care, University of Bergen, Bergen, Norway

14. Ryan White Center for Pediatric Infectious Disease and Global Health, Indiana University School of Medicine, Indianapolis, IN, USA

15. Division of Infectious Diseases, School of Medicine, University of North Carolina, Chapel Hill, USA

16. Department of Medicine Division of Transfusion Medicine and Program in Bioinformatics and Integrative Biology, University of Massachusetts School of Medicine, Worcester, USA

Madhumuni ya uchunguzi.

KEMRI/CDC na chuo kikuu cha Makerere wanafanya kazi na wizara ya afya ya Kenya na Uganda kufanya mradi wa utafiti wa malaria kutafuta njia bora ya kuzuia hatari ya watoto kufa au kurejeshwa hospitalini baada ya kutibiwa kwa ajili ya upungufu wa damu (makali ya upungufu wa damu).

Je, uchunguzi huu unahusu nini?

Watoto wanaoishi eneo la malaria ambao wametibiwa kwa ajili ya upungufu wa damu wana nafasi ya juu ya kuwa wagonjwa tena au kufariki katika miezi mitatu baada ya kuondoka hospitalini.

Madhumuni ya uchunguzi huu ni kutafuta njia ipi bora ya kuzuia watoto waliotibiwa kwa ajili ya makali ya upungufu wa damu kufa au kurejeshwa na kulazwa hospitalini tena. Tunataka kujua kama kuchukuwa dawa mpya inayoitwa DHA-piperaquine (DP) kwa wiki 2, 6, na 10 baada ya kutoka hospitalini ni bora kuliko kutumia Coartem® wakati wa kutoka hospitalini peke yake.

Mbona mtoto wako kachaguliwa?

Mtoto wako alilazwa na kutibiwa hospitalini kwa ajili alikuwa mgonjwa sana kutokana na upungufu wa damu. Wengi wa watoto walio katika hospitali hili wakiwa na ugonjwa sawa na hilo wataalikwa kushiriki. Kwa ujumla tutauliza watoto 2212 walio chini ya miaka 5 kushuriki katika uchunguzi huu.

Je, Ni nini litamtendekea mtoto wangu ikiwa atashiriki?

Ikiwa utaamua kushiriki, tutakuuliza maswali kuhusu ugonjwa wa mtoto wako na matibabu aliyoipata kabla ya kuja hospitalini na gharama yake. Tutakuuliza maswali kuhusu unapoishi, wakati wako wa kusafiri na inakugharimu pesa ngapi kuja hospitalini, muundo wa nyumba yako, masomo yako na chanzo kuu cha mapato kwa familiya.

Daktari wa uchunguzi au muuguzi atamchunguza mtoto wako tena. Tutanakili habari kuhusu ugonjwa wa mtoto wako kutoka kwa maelezo ya kliniki na maabara.

Ni nini kitafanyika na damu iliyochukuliwa katika utafiti huu?

Tutachukuwa kiasi kidogo cha damu zaidi (Kijiko kimoja cha chai) kutoka kwa vena ya mkono wa mtoto wako. Damu itapimwa kuchunguza malaria na upungufu wa damu. Upimaji huu unaeza kufanywa kwenye nyumba ya matibabu na tutaweza kukueleza majibu.

Kwa siku za usoni, tutaangalia mambo ambayo yanakinga dhidi ya malaria na ambayo yanasababisha upungufu wa damu kama ugonjwa wa sickle cell na thalassemia. Titaupima damu kuangalia kama madawa za malaria bado zinafanya kazi. Tutapima pia kama dudu za malaria zimebadilika kwa wakati.

Ni vipi kuhusu matibabu ya uchunguzi?

Kwa madhumuni ya uchunguzi huu tutawapa watoto wote Coartem mara watakapo kuwa sawa kula chakula tena, hata kama hawatakuwa wamepatikana na malaria wakati ambapo wamelazwa

hospitalini. Muuguzi wetu wa uchunguzi atampa mtoto wako dozi ya kwanza ya Coartem hapa hospitalini. Ikiwa mtoto wako yuko salama kutoka hospitalini itambidi achukuwe madawa mengine nyumbani hadi madawa ya siku 3 yamekamilika.

Je, ni nini litatendeka nikiondoka hospitalini?
Tutakuomba urudi hospitalini baada ya wiki 2 kwa ufwatilio. Wakati huo tutamweka mtoto wako kwenye matibabu ya uchunguzi.

Je, wataamua vipi matibabu ambayo mtoto wangu atapata?
Kuna matibabu aina mbili, DP na kipozaungo (tembe linalofanana na DP lakini halina dawa). Mtoto wako atapewa aina moja. Uchaguzi ambayo ni aina gani ya matibabu ya uchunguzi mtoto wako atapata ni kwa bahati. Matibabu ya uchunguzi hayo mawili yanafanana hata daktari wa uchunguzi anayemtibu mtoto wako hatajua ni aina gani kati ya madawa mawili ya uchunguzi mtoto wako atakuwa akichukuwa.

Je, dawa ya uchunguzi linachikuliwa vipi?
Matibabu litapeanwa mara moja kwa siku kwa siku 3. Muuguzi wetu wa uchunguzi atampa mtoto wako dozi ya kwanza ya matibabu hapa hospitalini au nyumbani. Watoto wote katika kila kikundi watapokea matibabu kama tembe lililo sagwa na kuyeyushwa na maji tamu. Dozi mbili inayo baki itachukuliwa nyumbani. Muuguzi wa uchunguzi atapiga simu kukukumbusha kumpa mtoto dawa kama italazimu.

Je, utafanya kitu chochote kingine katika wiki 2 ya matembezi?
Tutachukuwa damu kidogo kutoka kwa kisigino cha mtoto wako au kidole kupima upungufu wa damu. Kwa kuongezea tutampa mtoto wako tembe ya madini ya chuma kama matibabu ya upungufu wa damu mwilini. Nilazima achukuwe hizi kila siku kwa mwezi moja.

Je, nilazima nimrejeshe mtoto wangu?
Ndiyo, tutahitaji kumwona mtoto wako tena katika wiki 6 na tena wiki 10 kupeana matibabu zaidi ya uchunguzi kama ilivyo elezewa hapo juu. **Pia tutakupigia simu katika mwezi wako wa nne na tano ilikujua njisi mtoto wako anavyoendelea na kukumbusha ziara yako ya mwisho ya kliniki mwisho wa mwezi wako was sita (wiki ishirini na sita)**

Ziara ya mwezi 6
Tungependa kumwona mtoto wako mara ya mwisho baada ya miezi 6 kutoka leo kuona jinsi gani dawa imefanya kazi. Kwa wakati huo tutakuuliza maswali kuhusu afya ya mtoto wako, kuchunguza na kupima uzito na joto mwilini. Tutachukuwa damu kidogo kutoka kwa mtoto wako kwa kudunga kidole chake(Kiasi chini ya nusu kijiko cha chai). Damu litatumika kupima malaria na kupima kiwango cha damu yake.

Je, ni nini naweza kufanya?
Ikiwa utaamua kujiunga na uchunguzi tutakuhitaji uwepo kwa wakati wote kwa miezi 6 kutoka siku ulijiunga na uchunguzi.

Ni la muhimu mtoto kuchukuwa dawa zote alizopewa na uchunguzi. Usipeane dawa kwa watoto wako wengine hata kama wapo kwenye uchunguzi.

Watoto wanao shiriki katika uchunguzi wanaweza tu kupokea aina ya dawa fulani. Kwa hivyo tuna kuomba usinunue dawa ya ziada kwa mtoto wako kutoka madukani wakati wa uchunguzi. Ikiwa

mtoto wako ni mgonjwa wakati wowote katika miezi 6 ya uchunguzi unaweza kumleta mtoto wako katika kliniki ya uchunguzi hospitalini. Tutakulipia nauli ya shilingi 500([Kenya Ksh 500]/[Uganda the amount will be left blank per local regulations] kwa kila safari). [Kenya: tutalipa fedha za kulala hospitalini, chakula kwa wewe na mtoto wako mkilala hospitalini usiku] / [Uganda: utapokea fedha kwa kila matembezi kwa wakati wako na chakula]. Tutalipia fedha za hospitali zinazolingana na matibabu na fedha za kulazwa hospitalini zinazolingana na utafiti huu.

Je, ni nini madhara ya matibabu ya uchunguzi?

Madawa zote zina madhara. Kuna uwezekano mtoto wako atatapika baada ya kuchukuwa dawa. Kutapika ni kawaida, hasa mtoto akiwa na joto mwilini. Ikiwa mtoto atatapika ndani ya nusu saa, atapewa dosi ya matibabu nyingine. Ikiwa atatapika baada ya nusu saa, atarudia nusu ya dosi. Ikiwa atatapika baada ya saa moja, hakuna dosi ya ziada itahitajika. Tafadhali mweleze mfanyakazi wa uchunguzi haraka iwezekanavyo ikiwa mtoto wako ametapika dosi. Kwa sababu hii, tutakuuliza ubaki kwenye nyumba ya matibabu kwa muda usiopungua saa moja baada ya mtoto kutapika dosi ya kwanza ya dawa ya uchunguzi.

Ujumla matibabu ya uchunguzi inavumiliwa na watoto. Kesi chache watoto hulalamikia kuumwa kwa kichwa, kuhisi kisunzi, au kichefuchefu na kuumwa kwa tumbo. Kwa wengi wa watoto, hii siyo kali. Nyingi ya ishara hizi inaweza pia kusababishwa na malaria .

DP inaweza sababisha mabadiliko kidogo kwa jinsi moyo unavyopiga. Tutamwangalia mtoto wako ikiwa atalalamikia kuumwa na kifua au kupiga kasi kwa moyo. Tutafanya hivi kwa kupima moyo (ECG).

Je, ni madhara gani yapo kushiriki katika uchunguzi huu?

Ikiwa utaamua kujiunga na uchunguzi, madhara ni ya kiwango cha chini. Kunaweza kuwa na jeraha kidogo au maumivu kidogo kwenya kidole au mkono sehemu damu itachukuliwa. Aidha kuna nafasi ya kuambukizwa sehemu damu inachukuliwa. Nafasi hii ni kidogo kwa vile tunatumia vyombo visafi. Uchunguzi itakuhitaji uje hospitalini mara nyingi kushinda kawaida. Hii inaweza kuwa usumbufu. Tutakurejeshea nauli ya usafiri.

Je kuna mafao kwa mtoto wangu?

Hakuna mafao moja kwa moja kwa mtoto wako. Hata hivyo, tutalipia matibabu yote ya mtoto wako kwa miezi sita atakuwepo katika uchunguzi. Hii haitahusisha upasuaji au ajali au magonjwa yasiohusika na utafiti huu, isipokuwa kama inahusiana na uchunguzi. Kwa vile mtoto wako atatembelea kliniki mara kwa mara, wakati wa kipindi cha uchunguzi, kuna uwezekano wa malaria na magonjwa mengine kugunduliwa na kutibiwa haraka kuliko kawaida kwa mtoto wako.

Je, mambo yakienda mrama?

Mtoto wako atapokea huduma ya kimatibabu bila malipo kwa kuumia au magonjwa yatakayotokea kwa ajili ya mtoto wako kuwa katika uchunguzi huu. Katika wakati wa uchunguzi huu, tutakueleza kama tumekuta ugonjwa yoyote ambaya inayoweza hatarisha mtoto wako.

Je, kujihusisha kwangu katika uchunguzi huu litawekwa kwa usiri?

Ikiwa utatoa ridhaa kushiriki katika uchunguzi wa kiutafiti tutaweka jina la mtoto wako na habari yote tutakayopata kutoka kwako kama sehemu ya uchunguzi huu kwa siri kulingana na upeo wa sheria. Ni wafanyakazi wa uchunguzi na watu wa kamati ya usalama na mamlaka ya serikali wanaweza pitia rekodi iliyo na jina lako na la mtoto wako. Tutatumia habari uliyotupa kwa ajili ya

uchunguzi peke yake. Habari tutakayo kusanya inaweza tumiwa kwa pamoja na watu wengine kutoka taasisi na chi nyingine lakini jina lako na la mtoto wako halitaonekana katika ripoti yoyote.

Je itachukuwa muda gani?
Mtoto wako atashiriki katika uchunguzi katika muda wa miezi 6. Ziara hili la mrejeleo litajumuisha wiki 2, wiki 6, wiki 10 na mwisho wa mwezi wa sita kama ilivyoelezwa hapo juu. Ziara litachukuwa dakika 60 hadi 90.

Je, ni lazima mtoto wangu kushiriki?
Ni juu yako kuamua ikiwa unataka mtoto wako kushiriki au kutoshiriki. Ikiwa utaamua mtoto wako kushiriki utatia sahihi fomu ya ridhaa. Utakuwa na ruhusa ya kusitisha wakati wowote bila sababu. Ikiwa hutaki mtoto wako kushiriki sasa hivi, au siku za usoni, hili halita dhuru kiwango cha huduma mtoto wako atapokea.

Anwani kwa mawasiliano zaidi
[Kenya]
1. [Ikiwa unamaswali kuhusu uchunguzi huu, au unataka kuondoa mtoto wako kwenye uchunguzi, tafadhali wasiliana na Dr Simon Kariuki (Simu: 057 202 29 02) au Dr Titus Kwambai (Simu: 0723 354 238), KEMRI/CDC, P.O.Box 1578, Kisumu. Pia unaweza kuwasiliana na mfanyikazi wa uchunguzi hospitalini.
2. Ikiwa unamaswali kuhusu haki yako kama mshiriki wa uchunguzi huu, au unafikiri mtoto wako amepata madhara kwa ajili ya uchunguzi huu, tafadhali wasiliana na The Secretary, KEMRI Scientific and Ethics Review Unit, Mbagathi Rd. Nairobi, Kenya, PO Box 54840-00200, Nairobi; nambari ya simu: 020 2722541,020 2713349, au 0722-205901.]

[Uganda]
1. [Ikiwa unamaswali kuhusu uchunguzi huu, au unataka kuondoa mtoto wako kwenye uchunguzi , tafadhali wasiliana na Dr Richard Idro or Dr Robert Opoka on 0774 274173 or 0772996164. Pia unaweza kuwasiliana na mfanyikazi wa uchunguzi hospitalini.
2. Ikiwa unamaswali kuhusu haki yako kama mshiriki wa uchunguzi huu, au unafikiri mtoto wako amepata madhara kwa ajili ya uchunguzi huu, tafadhali wasiliana na Prof James Tumwine, Chairman of the Makerere University School of Medicine Research and Ethics Committee (SOMREC) kwa 0414530020.]

Tuna matumaini yakuwa majibu ya uchunguzi huu itasaidia kuboresha matibabu ya makali ya upungufu wa damu katika sehemu hii. Ahsante sana kwa wakati wako.

Utapewa fomu ya maelezo na fomu ridhaa yenye umetia sahihi ili uweke. Fomu moja nyingine itawekwa na mfanyikazi wa uchunguzi.

16.2.14. Consent statement for main trial (Kiswahili)

Uchunguzi wa PMC ya kuzuia malaria kwa kutumia dawa baada ya kuondoko hospitalini

Kauli ya ridhaa ya uchunguzi

Kichwa ya uchunguzi: Kuzuia malaria kwa dawa na matibabu ya kila mwezi kwa kutumia dihydroartemisinin-piperaquine kwa udhibiti wa makali na upungufu wa damu kwa watoto chini ya miaka mitano walio ruhusiwa kurudi nyumbani nchini Uganda na Kenya: Majaribio ya miaka mitatu ya udhibiti ubora wa kipozaungo bila mpangilio kwa viungo viwili, kundi sambamba, vituo mabalimbali.
(Nambari ya Mhusika)_______________________________ (Nakili Nambari ya Mhusika kutoka kwa Pre-enrollment CRF)

<table>
<tr><td rowspan="2">Nimeelezewa yaliyo hapo juu na ninakubali mtoto wangu kushuriki katika uchunguzi huu. Ninaelewa ya kuwa niko na uhuru wa kuchaguwa mtoto wangu kuwa katika uchunguzi huu na kwa kusema “HAPANA” haitakuwa na madhara yoyote kwa mtoto wangu. Ninakubali kuhojiwa na wafanyikazi wa utafiti huu na kwamba mtoto wangu achunguzwe na damu kidogo itolowe katika kidole au kisigino ili ichunguzwe kwa malaria, anemia, na jene ambazo yasababisha upungufu wa damu au kuwakinga dhidi ya malaria. Naeleawa ya kwamba mtoto wangu atashiriki kwa muda wa miezi 6. Nakubali kumleta mtoto wangu kwenye kliniki au wafanyi kazi wa uchunguzi huu waje kwangu kwa wiki 2, 6, 10 na kwa mwisho wa mwezi wa sita. Nakubali pia kumleta mtoto wangu kuwenye kliniki inapohitajika, au wafanyikazi wa uchunguzi waje kwangu au kuwasiliana nami kwa simu . Ninaelewa kuwa sehemu muhimu ya recodi ya afya ya mtoto wangu na ukweli yote yaliyo kusanyiwa inaweza tazamwa na wafanyakazi wengine kutoka KEMRI/CDC na chuo kikuu cha Makerere. Ninatoa ruhusa kwa watu hawa kufikia rekodi yangu na kuambatanisha na taasisi nyingine. Nina toa ruhusa kutumika kwa pamoja yaliyo kusanyiwa kupitia uchunguzi huu bila jina la mtoto wangu na anwani na wachunguzi wengine nje ya nchi ya Kenya na Uganda.</td><td colspan="2">Ikiwa unakubali tia mviringo “Ndiyo” Ikiwa umekataa tia mviringo “Hapana”</td></tr>
<tr><td>NDIYO</td><td>HAPANA</td></tr>
</table>

	Jina	Sahihi **au alama ya kidole cha gumba cha kushoto**	Tarehe ya leo
Mzazi anayetoa ridhaa kwa mtoto			
Shahidi*			
Mfanya kazi wa utafiti anayemshirikisha mshiriki			

* Mzazi au Mlinzi anaweza tia sahihi na shahidi, au kwa kutamka maneno ya ridhaa yake mbele ya shahidi ambaye ata tia sahihi.

16.2.15. Participant Information Sheet long-term storage / future studies (Kiswahili)

Uchunguzi wa PMC ya kuzuia malaria kwa kutumia dawa baada ya kuondoko hospitalini

Ridhaa ya kuhifadhi sampuli ya damu kwa muda mrefu kwa uchunguzi wa baadaye

Kichwa ya uchunguzi: Kuzuia malaria kwa dawa na matibabu ya kila mwezi kwa kutumia dihydroartemisinin-piperaquine kwa udhibiti wa makali na upungufu wa damu kwa watoto chini ya miaka mitano walio ruhusiwa kurudi nyumbani nchini Uganda na Kenya: Majaribio ya miaka mitatu ya udhibiti ubora wa kipozaungo bila mpangilio kwa viungo viwili, kundi sambamba, vituo mabalimbali.

Wachunguzi

Dr Richard Idro[3] *Dr Harriet Nambuya[5]* *Dr Magdalene Kuria[8]* *Prof Duolao Wang[1]*
Dr Robert Opoka[3] *Dr Simon Kariuki[2]* *Dr Aaron Samuels[2,9]* *Prof Chandy John[14]*
Dr. Aggrey Dhabangi[3] *Dr Titus Kwambai[2]* *Prof Kamija Phiri[12]* *Prof Jonathan J. Juliano[15]*
Dr Tom Ediamu[4] *Dr Martina Oneko[2]* *Prof Bjarne Robberstad[13]* *Prof Jeff Bailey[16]*
Dr Sophie Namasopo[5] *Dr Grace M Nalwa[7]* *Prof Brian Faraghar[1]* *Prof Feiko ter Kuile[1,2]*

Taasisi

1. Liverpool School of Tropical Medicine (LSTM), Liverpool, United Kingdom
2. KEMRI Centre for Global Health Research (CGHR), Kisian, Kenya
3. College of Health Sciences, Makerere University, Kampala Uganda
4. Hoima Regional Referral Hospital, Hoima, Uganda
5. Jinja Regional referral hospital, Uganda
6. Tororo Hospital, Tororo, Uganda
7. Migori County Referral Hospital, Migori, Kenya
8. Kisumu County Referral Hospital, Kisumu, Kenya
9. Division of Parasitic Diseases and Malaria, US Centers for Disease Control and Prevention (CDC), Atlanta, GA, USA
10. National Malaria Control Program, Ministry of Health Kenya, Nairobi, Kenya
11. Ministry of Health, Siaya County, Siaya, Kenya
12. College of Medicine, University of Malawi, Blantyre, Malawi
13. Centre for International Health, & Department of Global Public Health and Primary Care, University of Bergen, Bergen, Norway
14. Ryan White Center for Pediatric Infectious Disease and Global Health, Indiana University School of Medicine, Indianapolis, IN, USA
15. Division of Infectious Diseases, School of Medicine, University of North Carolina, Chapel Hill, USA
16. Department of Medicine Division of Transfusion Medicine and Program in Bioinformatics and Integrative Biology, University of Massachusetts School of Medicine, Worcester, USA

Utangulizi wa kuhifadhi sampuli kwa muda mrefu
Kuongezea juu ya mipangilio ya uchunguzi ambayo ushaelezewa, tunakuomba kuhifadhi sampuli kidogo ya damu ambayo imechukuliwa kwa mtoto wako kama sehemu ya uchunguzi huu. Tunakuuliza usome fomu hii na uulize maswali ambayo unaweza kuwa nayo kabla uamue kukubali.

Nini kitafanyikia sampuli zilizohifadhiwa?
Sampuli zitachukuliwa wakati uchunguzi utakapoanza (sampuli 1), mwisho (sampuli 1) na wakati wowote mtoto wako atakuwa mgonjwa wakati wa marejeleo itagandwa kwenye barafu na kuhifadhiwa kwa muda usiojulikana kwa uchunguzi wa siku za usoni. Sampuli itahifadhiwa Kisumu KEMRI/CDC Centre for Global Health Research, Kenya na/au Chuo Kikuu cha Makerere, Kampala, Uganda, na baadaye zitapelekwa kwenye maabara ya Uholanzi, Norway, Ulaya au Amerika kwa uchunguzi wa siku za usoni. Haitauzwa au kutumiwa kwa biashara.

Kiwango cha kitambulisho
Jina la mtoto wako halitakuwa kwa sampuli zitazohifadhiwa; baadala yake zitakuwa na nambari yasiyojulikana au nambari za siri. Jina la mtoto wako halitaonekana katika majibu yeyote au ripoti. Hautapewa majibu ya vipimo ya siku za usoni kuhusu magonjwa ama ama jene ambazo zinapitishwa kwa familia.

Ridhaa kabla ya kutumia
Ombi na ruhusa itachukuliwa kutoka kwa kamati ya utafiti ya wachunguzi (REC) kabla utumizi wa sampuli zilizohifadhiwa. Ombi litaeleza kwa makini madhumuni ya utumizi wa sampuli zilizohifadhiwa. REC ni kamati spesheli ambayo inaaangalia utafiti wa matibabu ili kukinga haki na wema wa binadamu waliyojitolea.

Kile kinachoweza kutumiwa kwa sampuli zilizohifadhiwa
1. Hii sampuli ya damu itatumiwa kwa uchunguzi wa upungufu wa damu na malaria
2. Vipimo hizi zitajumuisha jene yanayoweza kusababisha upungufu wa damu kwa watoto au kuwakinga na malaria kama ugonjwa waa sickle cell na thalassemia. Tutachunguza jene za dudu za malaria.

Majibu ya vipimo vyovyote za siku za usoni zitaandikwa na kuchapishwa kwa vitabu na kuwasilishwa au kwa mikutano za kisayansi.

Madhara
Madhara ni ya kiwango cha chini kwa mtoto wako kwa uchunguzi wa siku za usoni itakayofanywa kwa sampuli ya damu itakayohifadhiwa kwa sababu jina la mtoto wako haitakuwa kwa sampuli na majibu haitawekwa kwenye rekodi ya uchunguzi.

Mafao kwa mtoto wangu
Hakuna mafao moja kwa moja kwa kwako au kwa mtoto wako kutokana na utafiti wa siku za usoni kwa sampuli ya damu iliyohifadhiwa. Hata hivyo, kwa kuchunguza sampuli hizi tunaweza kujua/kujielimisha zaidi juu ya malaria na upungufu wa damu na hii inaweza kufaa watoto wengine ambao wanateseka na shida kama hizi kwa siku za usoni.

Haki ya kukataa
Ni juu yako kuamua ikiwa unataka damu ya mtoto wako kuhifadhiwa kwa uchunguzi wa siku za usoni au kutohifadhiwa. Ikiwa utaamua kuchagua damu ya mtoto wako ihifadhiwe utaulizwa utie sahihi

kwa fomu ya ridhaa. Ikiwa hautaki damu ya mtoto wako ihifadhiwe kwa uchunguzi wa siku za usoni, sasa, au kwa siku za usoni, hii haitadhuru kiwango cha huduma mtoto wako atapokea. Pia hii haitadhuru ushiriki wa mtoto wako katika uchunguzi kuu ya PMC au ushiriki kwa uchunguzi wa siku za usoni. Pia unaweza badili nia na kutoka kwa kuhifadhi sampuli wakati mtoto wako bado ako katika uchunguzi. Sampuli haitatumika kwa utafiti na itaharibiwa. Hata kama utatoa ridhaa mtoto wako bado atapokea huduma kama watoto wengine kwa uchunguzi.

Anwani kwa mawasiliano zaidi [ya Kenya]/[ya Uganda]
[Kenya]

3. [Ikiwa unamaswali kuhusu uchunguzi huu, au unataka kujiondoa kwenye uchunguzi , tafadhali wasiliana na Dr Simon Kariuki (Simu: 057 202 29 02) au Dr Titus Kwambai (Simu: 0723 354 238), KEMRI/CDC, P.O.Box 1578, Kisumu. Pia unaweza kuwasiliana na mfanyikazi yeyote wa uchunguzi huu kwenye hospitali.
4. Ikiwa unamaswali kuhusu haki yako kama mshiriki, au mtoto wako amepata madhara kwa sabau ya uchunguzi huu, au unataka kuwasiliana na mtu ambaye hahusiki na utafiti huu kwa karibu tafadhali wasiliana na The Secretary, KEMRI Scientific and Ethics Review Unit, Mbagathi Rd. Nairobi, Kenya, PO Box 54840-00200, Nairobi; nambari ya simu: 020 2722541, 020 2713349, au 0722-205901.]

[Uganda]

5. [Ikiwa unamaswali kuhusu uchunguzi huu, au unataka kuondoa mtoto wako kwenye uchunguzi, tafadhali wasiliana na Dr Richard Idro or Dr Robert Opoka on 0774 274173 or 0772 996164. Pia unaweza kuwasiliana na mfanyikazi yeyote wa uchunguzi huu kwenye hospitali.

Ikiwa unamaswali kuhusu haki yako kama mshiriki, au mtoto wako amepata madhara kwa sabau ya uchunguzi huu, au unataka kuwasiliana na mtu ambaye hahusiki na utafiti huu kwa karibu tafadhali wasiliana na Prof James Tumwine, Chairman of the Makerere University School of Medicine Research and Ethics Committee (SOMREC) kwa 0414530020.]

16.2.16. Consent statement for long-term storage / future studies (Kiswahili)

Uchunguzi wa PMC ya kuzuia malaria kwa kutumia dawa baada ya kuondoko hospitalini

Ridhaa ya kuhifadhi sampuli ya damu kwa muda mrefu kwa uchunguzi wa baadaye

Kichwa ya uchunguzi: Kuzuia malaria kwa dawa na matibabu ya kila mwezi kwa kutumia dihydroartemisinin-piperaquine kwa udhibiti wa makali na upungufu wa damu kwa watoto chini ya miaka mitano walio ruhusiwa kurudi nyumbani nchini Uganda na Kenya: Majaribio ya miaka mitatu ya udhibiti ubora wa kipozaungo bila mpangilio kwa viungo viwili, kundi sambamba, vituo mabalimbali.

(Nambari ya Mhusika)______________________________________ (Nakili Nambari ya Mhusika kutoka kwa Pre-enrollment CRF)

<table>
<tr><td rowspan="2">Nimeelezewa kuhusu kuhifadhiwa kwa damu ya mtoto wangu na ninakubali kuwa [KEMRI/CDC] au [chuo kikuu cha Makerere] kuhifadhi sampuli ya damu ya mtoto wangu angalau muda wa miaka 15 kwa ajili ya uchunguzi wa baadaye. Ninaelewa na ninakubali sampuli ya damu ya mtoto wangu kutumwa kwenya maabara ya Uholanzi, Norway, Uingereza au Marekani kwa uchambuzi baadaye. Ninaelewa kuwa ninaweza badilisha nia ili sampluli ya mtoto wangu isitumwe, usihifadhiwe na usitumike kwa uchunguzi siku za baadaye. Kufanya hivyo ninaweza mweleza [Dr Simon Kariuki ama Dr Titus Kwambai KEMRI/ CDC (kwa Kenya,)] / [Dr Richard Idro au Dr Robert Opoka [Chuo kikuu cha Makerere (Kwa Uganda)].</td><td colspan="2">Ikiwa unakubali tia mviringo “NDIYO” Ikiwa hukubali tia mviringo”HAPANA”</td></tr>
<tr><td>NDIO</td><td>HAPANA</td></tr>
</table>

	Jina	Sahihi **au alama ya kidole cha gumba cha kushoto**	Tarehe ya leo
Mzazi anayetoa ridhaa kwa mtoto			
Shahidi*			
Mfanya kazi wa utafiti anayemshirikisha mshiriki			

* Mzazi au Mlinzi anaweza tia sahihi na shahidi, au kwa kutamka maneno ya ridhaa yake mbele ya shahidi ambaye atatia sahihi.

16.2.17. *Participant Information Sheet for ECG sub study (Kiswahili)*

Uchunguzi wa PMC ya kuzuia malaria kwa kutumia dawa baada ya kuondoko hospitalini

Karatasi ya taarifa kwa mshiriki ECG utafiti ndogo

Kicwha cha Utafiti: Utafiti mdogo wa ECG kwenye utafiti mkubwa wa post-discharge Malaria chemoprevention kwa matibabu ya kila mwezi na dihydroartemisinin-piperaquine.

Wachunguzi

Dr Richard Idro[3]
Dr Robert Opoka[3]
Dr. Aggrey Dhabangi[3]
Dr Tom Ediamu[4]
Dr Sophie Namasopo[5]
Dr Harriet Nambuya[5]
Dr Simon Kariuki[2]
Dr Titus Kwambai[2]
Dr Martina Oneko[2]
Dr Grace M Nalwa[7]
Dr Magdalene Kuria[8]
Dr Aaron Samuels[2,9]
Prof Kamija Phiri[12]
Prof Bjarne Robberstad[13]
Prof Brian Faraghar[1]
Prof Duolao Wang[1]
Prof Chandy John[14]
Prof Jonathan J. Juliano[15]
Prof Jeff Bailey[16]
Prof Feiko ter Kuile[1,2]

Taasisi
1. Liverpool School of Tropical Medicine (LSTM), Liverpool, United Kingdom
2. KEMRI Centre for Global Health Research (CGHR), Kisian, Kenya
3. College of Health Sciences, Makerere University, Kampala Uganda
4. Hoima Regional Referral Hospital, Hoima, Uganda
5. Jinja Regional referral hospital, Uganda
6. Tororo Hospital, Tororo, Uganda
7. Migori County Referral Hospital, Migori, Kenya
8. Kisumu County Referral Hospital, Kisumu, Kenya
9. Division of Parasitic Diseases and Malaria, US Center for Disease Control and Prevention (CDC), Atlanta, GA, USA
10. National Malaria Control Program, Ministry of Health Kenya, Nairobi, Kenya
11. Ministry of Health, Siaya County, Siaya, Kenya
12. College of Medicine, University of Malawi, Blantyre, Malawi
13. Centre for International Health, &Department of Global Public Health and Primary Care, University of Bergen, Bergen, Norway
14. Ryan White Center for Pediatric Infectious Disease and Global Health, Indiana University School of Medicine, Indianapolis, IN, USA
15. Division of Infectious Diseases, School of Medicine, University of North Carolina, Chapel Hill, USA
16. Department of Medicine Division of Transfusion Medicine and Program in Bioinformatics and Integrative Biology, University of Massachusetts School of Medicine, Worcester, USA

Kuanzishwa

Madawa ya utafiti (DP) itakayotumika katika utafiti huu wa PMC inaweza kusababisha mabadiliko madogo na ya muda mfupi jinsi moyo unapika . Tungependa kuchunguza jinsi moyo unapika (ECG) ili kutathmini utendakazi wa moyo wa mtoto wako kwenye utafiti huu wa PMC kulingana na taratibu ambayo ilielezwa kwenu tayari.

Kwa nini mimi amechaguliwa?
Utafiti wa ECG ni utafiti mdogo ukioko ndani ya kubwa utafiti mkubwa wa PMC. Watoto sitini na sita 66 tu wanaweza kushiriki. Mtoto wako amechaguliwa kwa sababu hospitali hii ilichaguliwa kwa utaratibu huu.

Nini kitatokea kama nataka kuchukua sehemu?
Uchunguzi ya vile moyo unapika (ECG) itafanyika katika chumba maalum katika Hospitali hii na inahitaji mtoto kulala kwa mgogo. Daktari au muuguzi atambatisha bandechi kwa mikono, miguu na kifua na kuambatanisha na ECG mashine ambayo inaweza kupima kupiga kwa moyo. Utaratibu huu unachukua muda wa dakika 5 na hauna uchungu kwa sababu bandechi iliyobandikwa haitadugwa kwa mwili wa mototo. Matokeo ya ECG itatolewa mara moja na kuelezwa baada ya kusoma. Kama moyo wa mtoto wako utapatikana kwa kuwa usiokuwa wa kawaida, yeye atatumwa kwa mtaalamu moyo kwa utathmini na matibabu zaidi. Hii inaweza kusababisha kuchelewesha au zuio kwa kutumia madawa ya utafiti.

Ratiba ya matokeo ya upimaji ya jinsi moyo inapika itakukwa kama ifwatavyo; uchunguzi wa kwanza kabla ya kumeza madawa, na wapili, siku 3, masaa 4-6 baada ya kunywa dawa ya 3. Hii pia kufanyika kabla ya kuchukuwa dawa ijayo katika wiki 6 na wiki 10. Hivyo jumla itakuwa uchunguzi wa ECG mara sita (6).

Wakati huo huo wa kufanya uchunguzi wa ECG, daktari itachukua kiasi kidogo cha damu (200ul) kutoka kwa kidole cha mtoto wako kuangalia viwango vya dawa kwenye damu. Kwa jumla, vijiti sita (6) vya damu vitachukuliwa. Sampuli za damu hizi zilizochukuliwa kwa ajili ya uchambuzi katika maabara nchini Thailand. Ni muhimu kwamba sisi kuhakikisha kuwa dawa iliyochukuliwa inafikia kiwango kinachohitajika katika damu, hii itadhibitisha utendakazi wa dawa.

Itakuwaje kama sitaki kuwa katika utafiti?
Kama utaamua kutoshiriki kwa utafiti wa jinsi moyo unapika moyo, haitahathiri tiba na matunzo ya mtoto wako wakati atakuwa hapa hospitalini na hata kuathiri ushiriki wako katika utafiti mkuu PMC au ushiriki baadaye katika utafiti mwingine. Unaweza kubadili nia yako na kuondoka kutoka utafiti huu ECG wakati wowote bila kutoa sababu. Hata kama wewe utaondoka kutoka utafiti wa ECG, bado unaweza kuendelea katika utafiti kuu PMC. Wewe bado utapata huduma sawa na watoto wengine katika utafiti huu.

Mawasiliana ya ziada [Ya Kenya]/[Ya Uganda]
Kenya
1. Kama una maswali zozote kuhusu utafiti huu, ama unataka mtoto wako awache kushiriki katika utafiti huu, tafadhali wasiliana na Daktari Simon Kariuki (Nambari ya simu: 057 202 29 02) au Daktari Titus Kwambai (nambari ya simu: 0723 354 238), KEMRI/CDC, Sanduku la Posta 1578, Kisumu. Unaweza pia kuwasiliana na yeyote kati ya watafiti au wauuguzi katika hospitali.

2. Kama una maswali zozote kuhusu haki yako kama mgojwa wa utafiti, ama unafikiri mtoto wako amepata mathara yeyote kuhusiana na utafiti huu, tafadhali wasiliana na katibu, shirika la la utafiti wa afya la kenya (KEMRI), Scientific and Ethics Review Unit, Mbagathi Rd. Nairobi, Kenya, Sanduku la Posta 54840-00200, Nairobi; Nambari za simu: 020 2722541, 020 2713349, or 0722-205901.]

Uganda

1. Kama una maswali zozote kuhusu utafiti huu, ama unataka mtoto wako awache kushiriki katika utafiti huu, tafadhali wasiliana na Daktari Richard Idro au Daktari Robert Opoka (Nambari za simu: 0774 274173 or 0772 996164. Unaweza pia kuwasiliana na yeyote kati ya watafiti kwenye hospitali

2. Kama una maswali zozote kuhusu haki yako kama mgojwa wa utafiti, ama unafikiri mtoto wako amepata mathara yeyote kuhusiana na utafiti huu, tafadhali wasiliana Profesa James Tumwine, Mwenye kiti wa Makerere University School of Medicine Research and Ethics Committee (SOMREC) kwa nambari ya simu 0414530020.]

16.2.18. Consent statement for ECG sub study (Kiswahili)

Uchunguzi wa PMC ya kuzuia malaria kwa kutumia dawa baada ya kuondoko hospitalini

Taarifa ya ridhaa ECG utafiti mdogo

Kicwha cha Utafiti: Utafiti mdogo wa ECG kwenye utafiti mkubwa wa post-discharge Malaria chemoprevention kwa matibabu ya kila mwezi na dihydroartemisinin-piperaquine.

(Nambari ya Mhusika) (Nakili Nambari ya Mhusika kutoka kwa Pre-enrollment CRF).

<table>
<tr><td rowspan="2">• Nimeelezewa na ninahabari ya kuhusu utafiti mdogo wa ECG
• Naelewa hii inahusisha kufanya uchunguzi wa moyo jinsi moyo unapika mara 6 kwa jumla, ikiwa ni pamoja leo na katika siku 2, na tena 2 ECGs mwezi ujao na mwezi mmoja baada ya hapo.
• Naelewa kwamba kila uchunguzi wa jinsi moyo unapika inaweza kuchukua kati ya dakika 5 hadi 10.
• Naelewa kwamba wakati mwingine itanibidi kusubiri kwa saa 1 kabla ya daktari au muuguzi kupata nafasi ya kufanya uchunguzi wa jinsi moyo unapika moyo.
• Naelewa kwamba wao watachukua kiasi kidogo cha damu kutoka kwa mtoto wangu kufuatilia ngazi ya dawa wakati kila uchunguzi wa jinsi moyo unapika utafanywa.
• Mimi pia naelewa na kukubaliana kwamba sampuli za damu ya mtoto wangu inaweza kutumwa kwa maabara nchini Thailand, Uingereza au Marekani kwa ajili ya uchambuzi.
• Naelewa kwamba naweza kubadili mawazo yangu kuwa sampluli ya damu ya mtoto wangu isitumwe, kuhifadhiwa au kutumika kwa ajili ya utafiti wa baadaye. Kwa kufanya hivyo, naweza kuelezea Dkt. Simon Kariuki au Dkt. Titus Kwambai wa KEMRI / CDC (Nchini Kenya), Dkt. Richard Idro au Dkt. Robert Opoka wa Chuo Kikuu cha Makerere (Uganda)
• Nimeelezewa kwamba ni juu yangu mimi kujiunga na utafiti huu ECG na naweze kuondoka utafiti ECG wakati wowote nataka, bila madhara kwa mtoto wangu kuwa katika utafiti kuu.</td><td colspan="2">Kama unakubaliana weka mzuguko kwa “NDIYO” na kama haukubaliani weka alama ya mzunguko kwa “LA”</td></tr>
<tr><td>Ndiyo</td><td>La</td></tr>
</table>

	Jina	Sahihi **au alama ya kidole cha**	Tarehe ya leo
Mzazi anayetoa ridhaa kwa mtoto			
Shahidi*			
Mfanya kazi wa utafiti anayemshirikisha			

* Mzazi / mlezi unaweza anaweza kutia saini na shahidi, au kwa maneno kuchukuwa ridhaa mbele ya shahidi atakaetia saini.

16.3. APPENDIX III. TERMS OF REFERENCE OVERSIGHT COMMITTEES

16.3.1. Trial Management Group (TMG)

16.3.1.1. Purpose

The TMG is responsible for the administrative management and day to day running of the trial. There will be one TMG per country.

16.3.1.2. Membership

1. Country Co-Principal Investigator
2. Site clinicians
3. Trial Coordinator
4. Administrators
5. Others who are involved in the day to day running of the trial
6. Chief Investigator (ad hoc)

The TMC will be chaired by the Country Principal Investigator or the Trial Coordinator.

16.3.1.3. Responsibilities:

- Study planning
- Organisation of Trial Steering Committee and Data Monitoring and Ethics Committee (DMEC) meetings
- Provide risk report to regulators, manufacture and ethics committees
- SUSAR [Serious unexpected suspected adverse events] reporting
- Responsible for trial master file
- Budget administration and contractual issues
- Advice for lead investigators
- Organisation of central data management and sample collection

16.3.2. Trial Steering Committee (TSC)

16.3.2.1. Purpose

The purpose of this document is to provide the TSC with a guidance of the terms of reference with the understanding that the Committee carry out their functions.

Attend regular meetings that shall be scheduled by the Trial Manager to address points 1,3, 4 and to track the progress of the trial

16.3.2.2. Membership TSC

Independent members

1. Chair: [name and contact details]
2. Statistician: [name and contact details]
3. Paediatrician/medical officer [name and contact details]

Trial members

- The Chief Investigator and all Country Co-Principal Investigators.
- Other co-investigators and the trial statistician will attend the meetings if and when required.

16.3.2.3. Roles and Responsibilities TSC

The TSC is a trial governing body which includes a majority of its members who are independent of the trial management group. The TSC concentrates on the progress of the trial and ensures that the trial is conducted to the standards set out in the Guidelines for Good Clinical Practice with consideration given to participant safety and provision of informed consent.

- To evaluate the progress of the trial in relation to timeliness, data quality and other factors that can affect the overall objectives of the trial
- To ensure participant rights and safety are adhered to and that the protocol demands freely given informed consent
- To review relevant information from other sources
- To consider the recommendations of the Data Monitoring and Ethics Committee (DMEC) and in light of it to inform the Chief Investigator and TMG the need to changes to the trial protocol
- To inform the GlobVac programme on the progress of the trial and in exceptional circumstances to extend or to terminate the trial.
- To ensure that the trial results are disseminated appropriately and consideration be given to the implementation of the results into policy

16.3.2.4. Operational TSC

The CI will present the full protocol to the TSC as an agenda before the start-up of data collection. The TSC members shall review the time line set out in the protocol for participant recruitment, informed consent documents and plans for data safety monitoring.

The TSC shall see that the finalised protocol is sent to the sponsor and funders before the start of participant recruitment and data collection.

The TSC in its first meeting shall approve the nominated members of the DMEC and establish the DMEC which shall meet regularly to review and report on the data quality and the results of interim analyses.

In all their deliberations the TSC should consider any deviations from the trial protocol, participant safety and information provided to the participants and consenting procedures.

16.3.2.5. Frequency of Meetings

The TSC shall have an initial face to face start up meeting to discuss the protocol and establish the DMEC. A second meeting shall take place before the initiation of the trial to finalise the protocol and approve the commencement of the trial. Thereafter the TSC will normally meet once a year in the life span of the trial and one meeting at the closure of the trial.

The Chair and at least 2 of the three independent members together with the CI and trial co-ordinator shall constitute the quorum. If so required, in addition a member of the funder can be invited to attend the meetings.

16.3.2.6. Trial Reports and actions TSC meeting

The TSC shall provide at each meeting a summary report of their findings and recommendations which must be submitted to the funder, the Sponsor and the TMG.

If the TSC makes a recommendation that the trial should be stopped or suspended, the Sponsor will take the necessary action to ensure that new recruitment to the trial is stopped whilst the TSC report is evaluated and the Research Ethics Committee is informed.

16.3.3. Data Monitoring and Ethics Committee (DMEC)

16.3.3.1. Membership DMEC

1. Chair: [name and contact details]
2. Statistician: [name and contact details]
3. Paediatrician/medical officer [name and contact details]

16.3.3.2. Role DMEC

The DMEC consist of 3-4 members (including one or more clinicians and one statisticians, all with experience in clinical trials).

The DMEC shall assess the data regularly (before the annual TSC meeting) to review the data and the interim analysis. The assessment could be via email or other electronic medium annually prior to the TSC meeting. In the first year of recruitment more frequent assessment (bi-annually) is recommended for this trial with one face to face meeting at least once during the trial.

The members should be the only personnel to see the results separated by treatment group during the trial. They are independent and look at the trial from an ethical point of view of the participant safety, future patients and society in general. It is their responsibility to prevent patients being exposed to any excess risks by recommending to the Trial Steering Committee (TSC) for the trial suspension or termination early if the safety or efficacy results are sufficiently convincing. The trial statistician is usually invited to attend part of the DMEC meeting to present the most current data from the trial. This will be blinded, unless the DMEC specifically requests for an unblinded analysis.

16.3.3.3. Responsibilities DMEC

- To determine how frequently interim analysis of trial data should be undertaken.
- To consider the blinded or unblinded interim data from the trial and relevant information from other sources.
- To consider any requests for unblinding and release of interim trial data and to recommend to the TSC on the importance of this.
- To report (following each DMEC meeting) to the TSC and to recommend whether the trial should continue, the protocol be modified or the trial be stopped.

A full confidence report should be submitted in writing to the TSC at the end of each DMEC meeting

16.4. APPENDIX IV.DECLARATION OF HELSINKI

WORLD MEDICAL ASSOCIATION DECLARATION OF HELSINKI

Recommendations guiding physicians in
Biomedical research involving human subjects

Adopted by the 18th World Medical Assembly, Helsinki
, Finland, June 1964,
Amended by the 29th World Medical Assembly, Tokyo, Japan, October 1975,
35th World Medical Assembly, Venice, Italy, October 1983
41st World Medical Assembly Hong Kong, September 1989
and the
48th General Assembly, Somerset West, Republic of South Africa,
October 1996

INTRODUCTION

It is the mission of the physician to safeguard the health of the people. His or her knowledge and conscience are dedicated to the fulfillment of this mission.

The Declaration of Geneva of the World Medical Association binds the physician with the words, "The health of my patient will be my first consideration", and the International Code of Medical Ethics declares that, "A physician shall act only in the patient's interest when providing medical care which might have the effect of weakening the physical and mental condition of the patient."

The purpose of biomedical research involving human subjects must be to improve diagnostic, therapeutic and prophylactic procedures and the understanding of the aetiology and pathogenesis of disease.

In current medical practice most diagnostic, therapeutic or prophylactic procedures involve hazards. This applies especially to biomedical research.

Medical progress is based on research which ultimately must rest in part on experimentation involving human subjects.

In the field of biomedical research a fundamental distinction must be recognized between medical research in which the aim is essentially diagnostic or therapeutic for a patient, and medical research, the essential object of which is purely scientific and without implying direct diagnostic or therapeutic value to the person subjected to the research.

Special caution must be exercised in the conduct of research which may affect the environment, and the welfare of animals used for research must be respected.

Because it is essential that the results of laboratory experiments be applied to human beings to further scientific knowledge and to help suffering humanity, the World Medical Association has prepared the following recommendations as a guide to every physician in biomedical research involving human subjects. They should be kept under review in the future. It must be stressed that the standards as drafted are only a guide to physicians all over the world. Physicians are not relieved from criminal, civil and ethical responsibilities under the laws of their own countries.

Basic principles

1. Biomedical research involving human subjects must conform to generally accepted scientific principles and should be based on adequately performed laboratory and animal experimentation and on a thorough knowledge of the scientific literature.
2. The design and performance of each experimental procedure involving human subjects should be clearly formulated in an experimental protocol which should be transmitted for consideration, comment and guidance to a specially appointed committee independent of the investigator and the sponsor provided that this independent committee is in conformity with the laws and regulations of the country in which the research experiment is performed.
3. Biomedical research involving human subjects should be conducted only by scientifically qualified persons and under the supervision of a clinically competent medical person. The responsibility for the human subject must always rest with a medically qualified person and never rest on the subject of the research, even though the subject has given his or her consent.
4. Biomedical research involving human subjects cannot legitimately be carried out unless the importance of the objective is in proportion to the inherent risk to the subject.
5. Every biomedical research project involving human subjects should be preceded with careful assessment of predictable risks in comparison with foreseeable benefits to the subject or to others. Concern for the interests of the subject must always prevail over the interests of science and society.
6. The right of the research subject to safeguard his or her integrity must always be respected. Every precaution should be taken to respect the privacy of the subject and to minimize the impact of the study on the subject's physical and mental integrity and on the personality of the subject.
7. Physicians should abstain from engaging in research projects involving human subjects unless they are satisfied that the hazards involved are believed to be predictable. Physicians should cease any investigation if the hazards are found to outweigh the potential benefits.
8. In publication of the results of his or her research, the physician is obliged to preserve the accuracy of the results. Reports of experimentation not in accordance with the principles laid down in this Declaration should not be accepted for publication.
9. In any research on human beings, each potential subject must be adequately informed of the aims, methods, anticipated benefits and potential hazards of the study and the discomfort it may entail. He or she should be informed that he or she is at liberty to abstain from participation in the study and that he or she is free to withdraw his or her consent to participation at any time. The physician should then obtain the subject's freely-given informed consent, preferably in writing.
10. When obtaining informed consent for the research project, the physician should be particularly cautious if the subject is in a dependent relationship to him or her or may consent under duress. In that case the informed consent should be obtained by a physician who is not engaged in the investigation and who is completely independent of this official relationship.
11. In case of legal incompetence, informed consent should be obtained from the legal guardian in accordance with national legislation. Where physical or mental incapacity makes it impossible to obtain informed consent, or when the subject is a minor, permission from the responsible relative replaces that of the subject in accordance with national legislation. Whenever the minor child is in fact able to give a consent, the minor's consent must be obtained in addition to the consent of the minor's legal guardian.
12. The research protocol should always contain a statement of the ethical considerations involved and should indicate that the principles enunciated in the present Declaration are complied with.

Medical research combined with professional care (Clinical Research)

1. In the treatment of the sick person, the physician must be free to use a new diagnostic and therapeutic measure, if in his or her judgement it offers hope of saving life, re-establishing health or alleviating suffering.
2. The potential benefits, hazards and discomfort of a new method should be weighed against the advantages of the best current diagnostic and therapeutic methods.
3. In any medical study, every patient - including those of a control group, if any - should be assured of the best proven diagnostic and therapeutic method. This does not exclude the use of inert placebo in studies where no proven diagnostic or therapeutic method exists.
4. The refusal of the patient to participate in a study must never interfere with the physician-patient relationship.
5. If the physician considers it essential not to obtain informed consent, the specific reasons for this proposal should be stated in the experimental protocol for transmission to the independent committee (I,2).
6. The physician can combine medical research with professional care, the objective being the acquisition of new medical knowledge, only to the extent that medical research is justified by its potential diagnostic or therapeutic value for the patient.

Non-therapeutic biomedical research involving human subjects (Non-clinical biomedical research)

1. In the purely scientific application of medical research carried out on a human being, it is the duty of the physician to remain the protector of the life and health of that person on whom biomedical research is being carried out.
2. The subjects should be volunteers -- either healthy persons or patients for whom the experimental design is not related to the patient's illness.
3. The investigator or the investigating team should discontinue the research if in his/her or their judgement it may, if continued, be harmful to the individual.
4. In research on man, the interest of science and society should never take precedence over considerations related to the well-being of the subject.

16.5. APPENDIX V. BUDGET

Item Description	Total Cost KSH	Total Cost USD*
**PERSONNEL**		
Subtotal cost Personnel	KES 121,427,216	1,395,715 $
**SUPPLIES**		
Study Drugs /supplies	KES 121,427,216	262,113 $
Equipment (lab)	KES 121,427,216	45,400 $
Subtotal Supplies	KES 26,753,635	307,513 $
**CONTRACTUAL SERVICES**		
Transport costs	KES 121,427,216	145,345 $
Other costs	KES 7,872,874	90,493 $
Subtotal Services	KES 20,517,889	235,838 $
**TRAVEL**		
In-country work-related travel	KES 1,478,385	16,993 $
Subtotal Travel	KES 1,478,385	16,993 $
**TOTAL DIRECT COST**		
Total Direct Costs	KES 170,177,125	1,956,059 $
Indirect Costs (20% of personnel)	KES 24,285,443	279,143 $
**TOTAL COSTS**	KES 194,462,568	2,235,202 $

*The original budget is in Norwegian kroner and subject to exchange rate fluctuations

16.6. APPENDIX VI. BUDGET JUSTIFICATION

Funding has been approved for 3 years of field work starting in 2015 and 3 years of Phd training. The overall budget is about 2.4 million USD, including 400 USD for central sponsorship support, including for trial insurance and trial monitoring, a central safety register, data management support and for trial coordination, international travel and Phd training, and the remaining for the field work and laboratory assays at just under 1,000 USD per patient. The budget also includes 20% overheads as indirect costs.

16.7. APPENDIX VII. SPIRIT 2013 CHECKLIST CLINICAL TRIAL PROTOCOL

SPIRIT 2013 Checklist: Recommended items to address in a clinical trial protocol and related documents*

Section/item	Item No	Description	Addressed on page number
Administrative information			
Title	1	Descriptive title identifying the study design, population, interventions, and, if applicable, trial acronym	8
Trial registration	2a	Trial identifier and registry name. If not yet registered, name of intended registry	1
	2b	All items from the World Health Organization Trial Registration Data Set	10
Protocol version	3	Date and version identifier	1
Funding	4	Sources and types of financial, material, and other support	1, 69
Roles and responsibilities	5a	Names, affiliations, and roles of protocol contributors	8 and 70
	5b	Name and contact information for the trial sponsor	1
	5c	Role of study sponsor and funders, if any, in study design; collection, management, analysis, and interpretation of data; writing of the report; and the decision to submit the report for publication, including whether they will have ultimate authority over any of these activities	69
	5d	Composition, roles, and responsibilities of the coordinating centre, steering committee, endpoint adjudication committee, data management team, and other individuals or groups overseeing the trial, if applicable (see Item 21a for data monitoring committee)	113 to 115

Introduction			
Background and rationale	6a	Description of research question and justification for undertaking the trial, including summary of relevant studies (published and unpublished) examining benefits and harms for each intervention	18 to 19
	6b	Explanation for choice of comparators	20
Objectives	7	Specific objectives or hypotheses	20 to 21
Trial design	8	Description of trial design including type of trial (eg, parallel group, crossover, factorial, single group), allocation ratio, and framework (eg, superiority, equivalence, noninferiority, exploratory)	21
Methods: Participants, interventions, and outcomes			
Study setting	9	Description of study settings (eg, community clinic, academic hospital) and list of countries where data will be collected. Reference to where list of study sites can be obtained	21
Eligibility criteria	10	Inclusion and exclusion criteria for participants. If applicable, eligibility criteria for study centres and individuals who will perform the interventions (eg, surgeons, psychotherapists)	23 to 27
Interventions	11a	Interventions for each group with sufficient detail to allow replication, including how and when they will be administered	29 to 31
	11b	Criteria for discontinuing or modifying allocated interventions for a given trial participant (eg, drug dose change in response to harms, participant request, or improving/worsening disease)	31 to 33
	11c	Strategies to improve adherence to intervention protocols, and any procedures for monitoring adherence (eg, drug tablet return, laboratory tests)	34 and 31
	11d	Relevant concomitant care and interventions that are permitted or prohibited during the trial	34
Outcomes	12	Primary, secondary, and other outcomes, including the specific measurement variable (eg, systolic blood pressure), analysis metric (eg, change from baseline, final value, time to event), method of aggregation (eg, median, proportion), and time point for each outcome. Explanation of the clinical relevance of chosen efficacy and harm outcomes is strongly recommended	35

Participant timeline	13	Time schedule of enrolment, interventions (including any run-ins and washouts), assessments, and visits for participants. A schematic diagram is highly recommended (see Figure)	14, 15, 17 and 36
Sample size	14	Estimated number of participants needed to achieve study objectives and how it was determined, including clinical and statistical assumptions supporting any sample size calculations	40
Recruitment	15	Strategies for achieving adequate participant enrolment to reach target sample size	41
Methods: Assignment of interventions (for controlled trials)			
Allocation:			
Sequence generation	16a	Method of generating the allocation sequence (eg, computer-generated random numbers), and list of any factors for stratification. To reduce predictability of a random sequence, details of any planned restriction (eg, blocking) should be provided in a separate document that is unavailable to those who enrol participants or assign interventions	42
Allocation concealment mechanism	16b	Mechanism of implementing the allocation sequence (eg, central telephone; sequentially numbered, opaque, sealed envelopes), describing any steps to conceal the sequence until interventions are assigned	42
Implementation	16c	Who will generate the allocation sequence, who will enrol participants, and who will assign participants to interventions	42
Blinding (masking)	17a	Who will be blinded after assignment to interventions (eg, trial participants, care providers, outcome assessors, data analysts), and how	43
	17b	If blinded, circumstances under which unblinding is permissible, and procedure for revealing a participant's allocated intervention during the trial	43
Methods: Data collection, management, and analysis			
Data collection methods	18a	Plans for assessment and collection of outcome, baseline, and other trial data, including any related processes to promote data quality (eg, duplicate measurements, training of assessors) and a description of study instruments (eg, questionnaires, laboratory tests) along with their reliability and validity, if known. Reference to where data collection forms can be found, if not in the protocol	44

	18b	Plans to promote participant retention and complete follow-up, including list of any outcome data to be collected for participants who discontinue or deviate from intervention protocols	31 to 34
Data management	19	Plans for data entry, coding, security, and storage, including any related processes to promote data quality (eg, double data entry; range checks for data values). Reference to where details of data management procedures can be found, if not in the protocol	44
Statistical methods	20a	Statistical methods for analysing primary and secondary outcomes. Reference to where other details of the statistical analysis plan can be found, if not in the protocol	44
	20b	Methods for any additional analyses (eg, subgroup and adjusted analyses)	46
	20c	Definition of analysis population relating to protocol non-adherence (eg, as randomised analysis), and any statistical methods to handle missing data (eg, multiple imputation)	45
Methods: Monitoring			
Data monitoring	21a	Composition of data monitoring committee (DMC); summary of its role and reporting structure; statement of whether it is independent from the sponsor and competing interests; and reference to where further details about its charter can be found, if not in the protocol. Alternatively, an explanation of why a DMC is not needed	48and 115
	21b	Description of any interim analyses and stopping guidelines, including who will have access to these interim results and make the final decision to terminate the trial	49
Harms	22	Plans for collecting, assessing, reporting, and managing solicited and spontaneously reported adverse events and other unintended effects of trial interventions or trial conduct	49
Auditing	23	Frequency and procedures for auditing trial conduct, if any, and whether the process will be independent from investigators and the sponsor	54
Ethics and dissemination			
Research ethics approval	24	Plans for seeking research ethics committee/institutional review board (REC/IRB) approval	57

Protocol amendments	25	Plans for communicating important protocol modifications (eg, changes to eligibility criteria, outcomes, analyses) to relevant parties (eg, investigators, REC/IRBs, trial participants, trial registries, journals, regulators)	57
Consent or assent	26a	Who will obtain informed consent or assent from potential trial participants or authorised surrogates, and how (see Item 32)	57
	26b	Additional consent provisions for collection and use of participant data and biological specimens in ancillary studies, if applicable	58
Confidentiality	27	How personal information about potential and enrolled participants will be collected, shared, and maintained in order to protect confidentiality before, during, and after the trial	58
Declaration of interests	28	Financial and other competing interests for principal investigators for the overall trial and each study site	59
Access to data	29	Statement of who will have access to the final trial dataset, and disclosure of contractual agreements that limit such access for investigators	59
Ancillary and post-trial care	30	Provisions, if any, for ancillary and post-trial care, and for compensation to those who suffer harm from trial participation	61
Dissemination policy	31a	Plans for investigators and sponsor to communicate trial results to participants, healthcare professionals, the public, and other relevant groups (eg, via publication, reporting in results databases, or other data sharing arrangements), including any publication restrictions	62
	31b	Authorship eligibility guidelines and any intended use of professional writers	63
	31c	Plans, if any, for granting public access to the full protocol, participant-level dataset, and statistical code	64
Appendices			
Informed consent materials	32	Model consent form and other related documentation given to participants and authorised surrogates	72
Biological specimens	33	Plans for collection, laboratory evaluation, and storage of biological specimens for genetic or molecular analysis in the current trial and for future use in ancillary studies, if applicable	NA

16.8. APPENDIX VIII. EURARTESIM PACKAGE INSERT

PACKAGE LEAFLET: INFORMATION FOR THE USER

Eurartesim 320 mg/40 mg film-coated tablets
Piperaquine tetraphosphate/dihydroartemisinin

Read all of this leaflet carefully before you start using this medicine
- Keep this leaflet. You may need to read it again.
- If you have any further questions, ask your doctor or pharmacist.
- This medicine has been prescribed for you. Do not pass it on to others. It may harm them, even if their symptoms are the same as yours.
- If any of the side effects gets serious, or if you notice any side effects not listed in this leaflet, please tell your doctor or pharmacist.

In this leaflet:

1. What Eurartesim is and what it is used for
2. Before you or your child takes Eurartesim
3. How to take Eurartesim
4. Possible side effects
5. How to store Eurartesim
6. Further information

1. WHAT EURARTESIM IS AND WHAT IT IS USED FOR

Eurartesim contains the ingredients piperaquine tetraphosphate and dihydroartemisinin. It is used to treat uncomplicated malaria when use of a medicine given by mouth is appropriate.

Malaria is caused by infection with a parasite called *Plasmodium*, spread by the bite of an infected mosquito.There are different types of *Plasmodium* parasite. Eurartesim kills the *Plasmodium falciparum* parasite.

The medicine can be taken by adults, children and infants over 6 months old who weigh 5 kilograms or more.

2. BEFORE YOU OR YOUR CHILD TAKE EURARTESIM

Do not take Eurartesim if you or your child:
- is allergic (hypersensitive) to the active substances, piperaquine tetraphosphate or dihydroartemisinin, or to any of the other ingredients of Eurartesim (see section 6 for a list of these);
- has a severe type of malaria infection which has affected parts of your body such as the brain, lungs or kidneys;
- has a heart condition, such as changes to the rhythm or rate of your heart beat, or heart disease;
- knows that any member of your family (parents, grandparents, brothers or sisters) died suddenly due to a heart problem or was born with heart problems;
- suffers from changes to the levels of salts in your body (electrolyte imbalances);
- is taking other medicines that can have an effect on heart rhythm, such as:
  - quinidine, disopyramide, procainamide, amiodarone, dofetilide, ibutilide, hydroquinidine or sotalol;
  - medicines used to treat depression;
  - medicines used to treat mental health problems such as phenothiazines, sertindole, sultopride, chlorpromazine, haloperidol, mesoridazine, pimozide, or thioridazine;

48

- medicines used to treat infections. These include some of the types of medicines used to treat bacterial infections (macrolides [such as erythromycin or clarithromycin] and fluoroquinolones [such as moxifloxacin and sparfloxacin]) or fungal infections (including fluconazole and imidazole) as well as pentamidine (used to treat a specific type of pneumonia) and saquinavir (for treatment of HIV);
- antihistamines used to treat allergies or inflammation such as terfenadine, astemizole or mizolastine;
- certain medicines used to treat stomach problems such as cisapride, domperidone or droperidol;
- other medicines such as vinca alkaloids and arsenic trioxide (used to treat certain cancers), bepridil (used to treat angina), diphemanil (used to treat stomach disturbances), levomethadyl and methadone (used to treat drug addiction), and probucol (used to treat high blood cholesterol levels).

• has recently (for example within about one month) been treated for malaria with certain medicines or has taken certain medicines to prevent malaria. These medicines include: mefloquine, halofantrine, lumefantrine, chloroquine or quinine

If any of the above applies to you or your child or if you are unsure, tell your doctor or pharmacist before taking or giving Eurartesim.

Take special care with Eurartesim

Check with your doctor or pharmacist before taking this medicine if you or your child:

- has liver or kidney problems;
- has a malaria infection caused by a parasite other than *Plasmodium falciparum*;
- is taking or has taken any other medicines for the treatment of malaria (other than those mentioned above);
- is pregnant or breastfeeding (see below);
- is female, elderly (over 65 years) or vomiting;
- is taking certain other medicines which could cause possible metabolic interactions. Examples are listed in the section "Taking other medicines".

If you are not sure about any of the above, please ask your doctor or pharmacist.

Use in children

Do not give this medicine to infants under 6 months or below 5 kg in weight.

Taking other medicines

Please tell your doctor or pharmacist if you or your child is taking or has recently taken any other medicines, including medicines obtained without a prescription. Some medicines can affect the way Eurartesim works and your doctor may decide that Eurartesim is not suitable or that extra checks are needed while you or your child is taking the medicinal products which could cause possible interactions. Examples are listed below (but there are several others):

- some medicines used to treat high cholesterol in the blood (such as atorvastatin, lovastatin, simvastatin);
- medicines used to treat hypertension and heart problems (such as diltiazem, nifedipine, nitrendipine, verapamil, felodipine, amlodipine);
- some medicines used to treat HIV (antiretroviral medicinal products): protease inhibitors (such as amprenavir, atazanavir, indinavir, nelfinavir, ritonavir), non-nucleoside reverse transcriptase inhibitors (such as efavirenz, nevirapine);
- some medicines used to treat microbial infections (such as telithromycin, rifampicin, dapsone);
- medicines used to help you fall asleep: benzodiazepines (such as midazolam, triazolam, diazepam, alprazolam), zaleplon, zolpidem;
- medicines used to prevent/treat epileptic seizures: barbiturates (such as phenobarbital), carbamazepine or phenytoin;
- medicines used after organ transplantation and in autoimmune diseases (such as cyclosporin, tacrolimus);

- sex hormones, including those contained in hormonal contraceptives (such as gestodene, progesterone, estradiol), testosterone;
- glucocorticoids (hydrocortisone, dexamethasone);
- omeprazole (used to treat diseases related to gastric acid production);
- paracetamol (used to treat pain and fever);
- theophylline (used to improve bronchial air flow);
- nefazodone (used to treat depression);
- aprepitant (used to treat nausea);
- some gases (such as enflurane, halothane and isoflurane) used to give a general anaesthetic.

Taking Eurartesim without food and drink
You should take Eurartesim tablets with water only.
You should take this medicine on an empty stomach. You should take each dose no less than 3 hours after the last food intake, and no food should be taken within 3 hours after each dose of Eurartesim. You can drink water at any time. You should not take Eurartesim with grapefruit juice due to possible interactions.

Pregnancy and breast-feeding
Tell your doctor if you are pregnant, think you may be pregnant or become pregnant, or if you are breast-feeding.

Eurartesim must not be used in pregnancy if your doctor can give you an alternative medicine. If you receive Eurartesim while pregnant, please note that a pregnancy registry is in place to monitor the pregnancy outcomes.

You should not breast-feed your baby while taking this medicine.

If you are taking folate supplements to prevent possible neural tube birth defects, you can continue taking them at the same time as Eurartesim.

Ask your doctor or pharmacist for advice before taking any medicine during pregnancy or breast-feeding.

Driving and using machines
You can drive or use machines after taking Eurartesim.

3. HOW TO TAKE EURARTESIM

Always take Eurartesim exactly as your doctor has told you to. You should check with your doctor or pharmacist if you are not sure.

Take this medicine with water and on an empty stomach. You or your child should take each dose at least 3 hours after your last meal. You should also avoid eating until 3 hours after taking Eurartesim. You can drink water at any time.
If the tablets are difficult to swallow, you can crush and mix them with water; drink the mixture immediately.

A course of Eurartesim lasts 3 consecutive days. Take one dose on each day. You should try to take the dose at about the same time on each of the three days.

The daily dose depends on the patient's **body weight**. Your doctor should have prescribed a dose that is appropriate for your weight or your child's weight as follows:

Body weight (kg)	Daily dose (mg)	Total number of tablets for treatment
5 to less than 7	Half 160 mg/20 mg tablet a day	1.5 tablet
7 to less than 13	One 160 mg/20 mg tablet a day	3 tablets
13 to less than 24	One 320 mg/40 mg tablet a day	3 tablets
24 to less than 36	Two 320 mg/40 mg tablets a day	6 tablets
36 to less than 75	Three 320 mg/40 mg tablets a day	9 tablets
75 to 100	Four 320 mg/40 mg tablets a day	12 tablets

If you weigh more than 100 kg then follow the dose that your doctor has prescribed.

Vomiting when taking this medicine
If this happens within:
- 30 minutes of taking Eurartesim, the whole dose must be taken again.
- 31-60 minutes, half the dose must be taken again.

If you or your child vomit also the second dose, do not take or give your child another dose. Contact your doctor urgently to obtain an alternative treatment for malaria.

Taking this medicine, if the malaria infection returns
- If you or your child gets another attack of malaria you may take a second course of Eurartesim within one year if your doctor thinks this is a suitable treatment. You or your child must not take more than two courses within one year. If this happens, talk to your doctor. You or your child should not take a second course of Eurartesim within 2 months of the first course.
- If you or your child is infected more than twice in a year, your doctor will prescribe an alternative treatment.

If you or your child takes more Eurartesim tablets than you should
If you or your child takes more than the recommended dose, tell your doctor. Your doctor may suggest special monitoring for you or your child because doses higher than those recommended may have an unwanted, severe effect on your heart (see also section 4).

If you or your child forgets to take Eurartesim
If you or your child forgets to take the second dose of Eurartesim at the right time, take it as soon as you remember. Then take the third (last) dose approximately 24 hours after the second dose.
If you or your child forgets to take the third (last) dose at the right time, take it as soon as you remember.
Never take more than one dose on the same day to make up for a missed dose.
Check with your doctor or pharmacist if you are not sure.

If you or your child stops taking Eurartesim
For the medicine to work effectively, you or your child should take the tablets as instructed and should complete the 3 days course of treatment. If you or your child is not able to do this, talk to your doctor or pharmacist.

If you have any further questions on the use of this medicine, ask your doctor or pharmacist.

4. POSSIBLE SIDE EFFECTS

Like all medicines, Eurartesim can cause side effects, although not everybody gets them. Most of the side effects are not severe and normally disappear within a few days or weeks after treatment.

If you or your child gets a rash, swelling of the face, lips, tongue or throat with difficulty in swallowing or breathing, these may be signs of an allergic reaction. Tell your doctor immediately, or go immediately to the emergency department of your nearest hospital, taking this leaflet with you.

A heart problem, called QT prolongation, can occur while taking Eurartesim and for some days after taking the last dose. This can cause a life-threatening abnormality of the heart rhythm.
Your doctor may take electrical recordings of your heart (electrocardiogram, ECG) while you are being treated and after the last dose is given. Your doctor will advise you when these readings will be taken.
If you notice anything different about your heart rhythm or have symptoms (such as palpitations or irregular heart beat) you should contact your doctor as soon as possible and before the next dose is due.

Side effects in adults

Common (affecting less than 1 in 10 patients but more than 1 in 100)
Anaemia, headache, heart rhythm disturbances (ECG changes or noticing unusually fast heart beats or palpitations), fever, general weakness.

Uncommon (affecting less than 1 in 100 patients but more than 1 in 1000)
Influenza, respiratory infections, poor appetite or loss of appetite, dizziness, convulsions (fits), irregular or slow heart rate, cough, vomiting, abdominal pain, diarrhoea, nausea, inflammation or enlargement of the liver, abnormal liver function tests, itching, pain in the muscles or joints.

Side effects in children

Very common (affecting more than 1 in 10 patients)
Influenza, cough, fever.

Common (affecting less than 1 in 10 patients but more than 1 in 100)
Respiratory infections, ear infection, anaemia, abnormalities in various types of blood cells (white blood cells and platelets), poor appetite or loss of appetite, eye infection, heart rhythm disturbances (change as in adults, ECG changes), abdominal pain, vomiting, diarrhoea, skin inflammation, rash, general weakness.

Uncommon (affecting less than 1 in 100 patients but more than 1 in 1000)
Abnormalities in red blood cells, excessive numbers of platelets, enlargement of some organs (such as liver or spleen), swollen lymph glands, convulsions (fits), headache, abnormal heart sounds (heard by your doctor with a stethoscope), nose bleeds, runny nose, nausea, inflammation of the mouth, inflammation or enlargement of the liver, jaundice, abnormal liver function blood tests, skin itching and inflammation, pain in the joints.

If any of the side effects gets serious, or if you notice any side effects not listed in this leaflet, please tell your doctor or pharmacist.

5. HOW TO STORE EURARTESIM

Keep Eurartesim tablets out of the reach and sight of children.

Do not take Eurartesim after the expiry date which is stated on the package after 'EXP'. The expiry date refers to the last day of that month.

Do not store above 30°C.
Store in the original package in order to protect from light and moisture.

Do not use Eurartesim if you notice the blister package is open.

Medicines should not be disposed of via wastewater or household waste. Ask your pharmacist how to dispose of medicines no longer required. These measures will help to protect the environment.

6. FURTHER INFORMATION

What Eurartesim contains
Each film-coated tablet contains 320 mg piperaquine tetraphosphate (as the tetrahydrate) and 40 mg dihydroartemisinin.

The other ingredients are:
Tablet core: pre-gelatinised starch, dextrin, hypromellose (E464), croscarmellose sodium, magnesium stearate (E572).
Film coating: hypromellose, titanium dioxide (E171), macrogol 400.

What Eurartesim looks like and contents of the pack
Eurartesim are white film-coated tablets, embossed and with a break line along the middle.

The 320 mg/40 mg tablets have two 'σ' letters on one side and come in blister strips containing 3, 6, 9 or 12 tablets.

Marketing Authorisation Holder

Sigma-Tau Industrie Farmaceutiche Riunite s.p.a.
Viale Shakespeare 47
00144 Rome
Italy

Tel: +39 06 5926443
Fax: +39 06 5926600
Email: sigmatauinfo@sigma-tau.it

Manufacturer

Sigma-Tau Industrie Farmaceutiche Riunite s.p.a.
Via Pontina km. 30,400
00040 Pomezia (Rome)
Italy

Tel: +39 06 91391
Fax: +39 06 91166976
Email: sigmatauinfo@sigma-tau.it

For any information about this medicine, please contact the local representative of the Marketing Authorisation Holder:

België/Belgique/Belgien
MPCA bvba
Tél/Tel: + 32 9 344 53 38

Luxembourg/Luxemburg
MPCA bvba
Belgique/Belgien
Tél/Tel: + 32 9 344 53 38

Nederland
Sigma-Tau BV
Tel: +31 30 6702020
info@sigma-tau.nl

Deutschland
Sigma-Tau Arzneimittel GmbH
Tel.: +49 211 687717 0
info@sigma-tau.de

España
Sigma-Tau España, S.A.
Tel: +34 91 888 36 00
sigmatau@sigma-tau.es

Portugal
Defiante Farmacêutica, S.A.
Tel: +351 291 214 090
info@defiante.com

France
Sigma-Tau France
Tél: + 33 1 45 21 02.69

United Kingdom
Sigma-Tau Pharma Limited UK
Tel: +44 (0) 8000431268
medical.information@sigma-tau.co.uk

Ireland
Sigma-Tau Pharma Limited UK
United Kingdom
Tel: +44 (0) 8000431268
medical.information@sigma-tau.co.uk

България, Magyarország, Ceská republika, Malta, Danmark, Norge, Eesti, Österreich, Ελλάδα, Polska, România, Slovenija, Ísland, Slovenská republika, Italia, Suomi/Finland, Κύπρος, Sverige,Latvija, Lietuva

Sigma-Tau Industrie Farmaceutiche Riunite s.p.a.
Италия, Olaszország, Itàlie, Italja, Italien, Italia, Itaalia, Ιταλία, Włochy, Italija, Ítalía, taliansko, Itālija
Тел/Tel/Tlf/Τηλ/Sími/Puh: +39 06 9139.3796
Daniela.Campanelli@sigma-tau.it

This leaflet was last revised in

Detailed information on this medicine is available on the European Medicines Agency web site:
http://www.ema.europa.eu

54

16.9. APPENDIX IX. QUESTIONNAIRES

To be designed
