# Supplementary material for: Malaria chemoprevention with monthly dihydroartemisinin-piperaquine for the post-discharge management of severe anaemia in children aged less than 5 years in Uganda and Kenya: study protocol for a multi-centre, two-arm, randomised, placebo-controlled, superiority trial
Source: Trials. 2018 Nov 6;19:610. doi: 10.1186/s13063-018-2972-1 (PMC6220494; doi:10.1186/s13063-018-2972-1)
Supplement: Supplementary file 2 — Ethics approvals: KEMRI, SOMREC, LSTM, REK vest and CDC. (ZIP 1940 kb) [file 13063_2018_2972_MOESM2_ESM.zip › 6919 CDC Continuation Approval 2.docx]

#
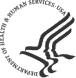
Memorandum

September 25, 2017January 21st, 2015

Date

d

From

Denise M. Marshall, BS

IRB Administrator, Human Research Protection Office

Subject

CDC Approval of Continuation #2 of Protocol #65116919.0, “The Sustainable Health Center Implementation PrEP Pilot (SHIPP) StudyMalaria Chemoprevention with monthly treatment with dihydroartemisinin-piperaquine for the post-discharge management of severe anaemia in children aged less than 5 years in Uganda and Kenya: A 3-year,multi-centre,parallel-group,two-arm randomized placebo controlled superiority trial” (Expedited)

To

Aaron Samuels, MD, MHS

CGH/DPDM

CDC's Human Research Protection Office reviewed and approved the request to continue

reliance on a non-CDC IRB for protocol #6919.0, “Malaria Chemoprevention with monthly treatment with dihydroartemisinin-piperaquine for the post-discharge management of severe anaemia in children aged less than 5 years in Uganda and Kenya: A 3-year,multi-centre,parallel-group,two-arm randomized placebo controlled superiority trial”, in accordance with 45 CFR 46.114. The protocol has been reviewed and approved by the KEMRI IRB for twelve months, and the IRB’s approval will expire on 09/19/2018.

Please submit CDC form 0.1251, Request for Continuing Review of IRB-Approved Protocol, along with certification of current IRB review and approval at the relied-upon institution, approximately six weeks prior to the protocol’s expiration date, even if you do not yet have certification of continuation approval to include with your submission. Please state on the 0.1251 that certification of continuation approval will be forwarded as soon as it is received.

Any problems of a serious nature should be brought to the immediate attention of the Human

Research Protection Office.

If you have any questions, please contact your National Center Human Subjects Contact or the CDC Human Research Protection Office at (404) 639-7570 or via e-mail: [huma@cdc.gov](mailto:huma@cdc.gov).

cc:

CGH Human Subjects Review (CDC)
