# Supplementary material for: Malaria chemoprevention with monthly dihydroartemisinin-piperaquine for the post-discharge management of severe anaemia in children aged less than 5 years in Uganda and Kenya: study protocol for a multi-centre, two-arm, randomised, placebo-controlled, superiority trial
Source: Trials. 2018 Nov 6;19:610. doi: 10.1186/s13063-018-2972-1 (PMC6220494; doi:10.1186/s13063-018-2972-1)
Supplement: Supplementary file 2 — Ethics approvals: KEMRI, SOMREC, LSTM, REK vest and CDC. (ZIP 1940 kb) [file 13063_2018_2972_MOESM2_ESM.zip › KEMRI Ethics Approval.pdf]

KENYA MEDICAL RESEARCH INSTITUTE

P.O. Box 54840-00200, NAIROBI, Kenya
Tel: (254) (020) 2722541, 2713349, 0722-205901, 0733-400003, Fax: (254) (020) 2720030
E-mail: director@kemri.org, info@kemri.org, Website. www.kemri.org

KEMRI/RES/7/3/1 **March 15, 2018**

TO: **PROF. FEIKO TER KUILE, DR. TITUS KWAMABAI AND DR. SIMON KARIUKI,**
PRINCIPAL INVESTIGATORS.

THRO: **THE DIRECTOR, CGHR,**
KISUMU.

FORWARDED
DIRECTOR - CGHR
21/3/2018

Dear Sir (s),

RE: **SSC 2965 *(AMENDMENT 3)*: MALARIA CHEMOPREVENTION WITH MONTHLY TREATMENT WITH DIHYDROARTEMISIN-PIPERAQUINE FOR THE POST DISCHARGE MANAGEMENT OF SEVERE ANAEMIA IN CHILDREN AGED LESS THAN 5 YEARS IN UGANDA AND KENYA: A 3-YEAR, MULTI-CENTER PARALLEL-GROUP, TWO ARM RANDOMIZED PLACEBO CONTOROLLLED SUPERIORITY TRIAL.**

This is to inform you that at the 273rd Committee A meeting of the KEMRI Scientific and Ethics Review Unit (SERU) held on **March 13 2018**, the request for amendment for the above referenced research proposal was discussed.

The SERU Committee acknowledges receipt of the following documents:

1. PMC 2965 SERU Amendment form v4.0 dated 6th February 2018
2. PMC protocol V4.0 dated 06 Feb 2018 clean and tracked version showing changes in BOLD relative to v3.0 dated 28th October 2016

The Committee noted the following amendment;

1. Page 10: Target sample size Revised to 1040 from original: 2212.
2. Page 15: **Narrative protocol summary Sample size:**
   Sample size revised from 1106 to 520 children per arm and 2212 to 1040 for total children.
3. Page 22: **Design and methodology** -Overview study design
   Sample size revised from 2212 to 1040 children; the number per study arm changed from 1106 to 520 less than 5 years of age who have been admitted for all cause severe anaemia and have completed the standard in-hospital treatment.
4. **Page 41: Sample Size**
   The following text has been added
   8.8.1.1. Sample size re-estimation
   Following recommendations from the DMEC and TSC, a blinded interim sample size re-estimation was conducted to take the lower than expected rate of loss to follow-up into account and the higher than expected pooled event rate of the composite primary endpoint (death or All-cause readmission) across both arms. This was favor (I over an

interim analysis, because the available funding did not allow and extension: 1' the recruitment period, even if the results of any Interim analysis would suggest his was required.

The revised sample size calculations were conducted in PASS (v15) software 'sing a test for the ratio of two Poisson rates. A total sample size of 1040 children (520 per arm) is required to detect a 25% reduction in the incidence of the composite primary outcome from 1,152 per 1000 child years (530 events per 1000 children during the 24 weeks from randomization at2 weeks to the end of follow-up at 26 weeks) in the control arm to 864 per 1000 child years (398 per 1000 children over 24 weeks) in the intervention arm (power 80%, a=0.05), allowing for 10% loss to follow-up. The same sample size would also provide 90% power to detect a 28.7% reduction in the primary endpoint from 1,152 to 822 events per 1000 children years

The Committee concluded that the amendments are justified and do not alter the risk/benefit status of the study and are therefore **granted approval** for implementation. You are also required to submit any further requests for changes to the approved protocol to SERU prior to initiation.

Yours faithfully,

for **THE HEAD,**
KEMRI SCIENTIFIC AND ETHICS REVIEW UNIT.
