# Supplementary material for: Malaria chemoprevention with monthly dihydroartemisinin-piperaquine for the post-discharge management of severe anaemia in children aged less than 5 years in Uganda and Kenya: study protocol for a multi-centre, two-arm, randomised, placebo-controlled, superiority trial
Source: Trials. 2018 Nov 6;19:610. doi: 10.1186/s13063-018-2972-1 (PMC6220494; doi:10.1186/s13063-018-2972-1)
Supplement: Supplementary file 2 — Ethics approvals: KEMRI, SOMREC, LSTM, REK vest and CDC. (ZIP 1940 kb) [file 13063_2018_2972_MOESM2_ESM.zip › REK VEST 2018.pdf]

REK
REGIONALE KOMITEER FOR MEDISINSK OG HELSEFAGLIG FORSKNINGSETIKK

Region:	Saksbehandler:	Telefon:	Vår dato:	Vår referanse:
REK vest	Fredrik Rongved	55978498	22.12.2017	2014/1911/REK vest
			**Deres dato:**	**Deres referanse:**
			11.12.2017	Not required for trials outside the EU

Vår referanse må oppgis ved alle henvendelser

Bjarne Robberstad
Senter for Internasjonal Helse/Institutt for Global helse og Samfunnsmedisin

2014/1911 Malariaførebyggjing etter behandling av alvorleg anemi (PDM)

Institution responsible for the research: Liverpool School of Tropical Medicine, University of Bergen
Chief Investigator: Bjarne Robberstad

With reference to your application regarding the above-mentioneded project. Chair of The Regional Committee for Medical and Health Research Ethics of Western Norway (REC West) reviewed the application pursuant to The Health Research Act § 11.

Assessment
Change to project

Chief investigator wish to change the sample size from 2212 children to about 1066.

The Chair of REC West assessed the changes.

Assessment

REC West has no objections to the proposed amendment of the study.

Decision
REC West approves the project amendment in accordance with the submitted application.

The Access to Appeal
You have access to appeal the decision. The appeal should be sent to REC West. The deadline for appeals is three weeks from the date on which you receive this letter. REC can either grant your appeal or forward it to the National Committee for Research Ethics in Norway (NEM). The decision by NEM is final.

Sincerly

Marit Grønning
Prof. dr.med.
Chair

Fredrik Rongved
Committee Secretary

Kopi til: *post@uib.no; carl.henry@lstmed.ac.uk*
