# Supplementary material for: Malaria chemoprevention with monthly dihydroartemisinin-piperaquine for the post-discharge management of severe anaemia in children aged less than 5 years in Uganda and Kenya: study protocol for a multi-centre, two-arm, randomised, placebo-controlled, superiority trial
Source: Trials. 2018 Nov 6;19:610. doi: 10.1186/s13063-018-2972-1 (PMC6220494; doi:10.1186/s13063-018-2972-1)
Supplement: Supplementary file 2 — Ethics approvals: KEMRI, SOMREC, LSTM, REK vest and CDC. (ZIP 1940 kb) [file 13063_2018_2972_MOESM2_ESM.zip › REK-vest 2014-1911 Approval PMC v3.0 (amendment) 28Oct16.pdf]

REK
REGIONALE KOMITEER FOR MEDISINSK OG HELSEFAGLIG FORSKNINGSETIKK

Region:	Committee Secretary:	Phone:	Our date:	Reference
REK vest	Øyvind Straume	+47 55978497	08.11.2016	2014/1911/REK vest
			**Your date:** 31.10.2016	

Bjarne Robberstad

Senter for Internasjonal Helse/Institutt for Global helse og Samfunnsmedisin

2014/1911 Malariaførebyggjing etter behandling av alvorleg anemi (PDM)

Institution responsible for the research: Liverpool School of Tropical Medicine, University of Bergen
Project manager: Bjarne Robberstad

With reference to your application regarding the abovementioneded project. Chairman of The Regional Committee for Medical and Health Research Ethics (REC Western Norway) reviewed the application pursuant to The Health Research Act § 11

Assesment

Change to project
The project manager wants to add malaria smears to RDT as option to diagnose clinical malaria for unscheduled visits.

Chairman of REC Western Norway reviewed the change

Assessment
REC Western Norway has no objections to the proposed amendment of the study.

Decision
REC Western Norway approves the project amendment in accordance with the submitted application.

The decision of the committee may be appealed to the National Committee for Research Ethics in Norway. The appeal should be sent to the Regional Committee for Research Ethics in Norway, West. The deadline for appeals is three weeks from the date on which you receive this letter.

Sincerly

Ansgar Berg
Prof. Dr.med
Chairman

Øyvind Straume
Committee Secretary

Kopi til: *postmottak@uib.no*
