# Supplementary material for: Malaria chemoprevention with monthly dihydroartemisinin-piperaquine for the post-discharge management of severe anaemia in children aged less than 5 years in Uganda and Kenya: study protocol for a multi-centre, two-arm, randomised, placebo-controlled, superiority trial
Source: Trials. 2018 Nov 6;19:610. doi: 10.1186/s13063-018-2972-1 (PMC6220494; doi:10.1186/s13063-018-2972-1)
Supplement: Supplementary file 2 — Ethics approvals: KEMRI, SOMREC, LSTM, REK vest and CDC. (ZIP 1940 kb) [file LSTM-REC 14.034 Approval PMC Protocol v4.0.pdf › LSTM-REC 14.034 Approval PMC Protocol v4.0.pdf]

LSTM
LIVERPOOL SCHOOL OF TROPICAL MEDICINE
Pembroke Place,
Liverpool, L3 5QA, UK
Tel: +44(0)151 705 3100
Fax: +44(0)151 705 3370
www.lstmed.ac.uk

Professor Feiko ter Kuile
Liverpool School of Tropical Medicine
Pembroke Place
L3 5QA

Friday, 13 April 2018

Dear Professor ter Kuile,

Research Protocol (14-034) Malaria chemoprevention with monthly treatment with dihydroartemisinin-piperaquine for the post-discharge management of severe anaemia in children aged less than 5 years in Uganda and Kenya: a 3-year, multi-centre, parallel-group, two-arm, randomized placebo controlled trial (PMC study)

Protocol Number	Date	Date Received
v4.0	06 February 2018	07 February 2018

Thank you for your correspondence of 7 February 2018 providing the LSTM Research Ethics Committee and LSTM Research Governance Manager with details of an amendment to protocol.

This amendment has now been reviewed, noted and accepted on the behalf of the Committee and the LSTM, as study Sponsor. Please continue to adhere to the conditions of approval and to update us of any further changes to the study that may arise.

Please continue to adhere to the original conditions of approval following notification of study conclusion with specific regard to confidentiality and data management.

Yours sincerely,

Angela Obasi

Dr Angela Obasi
Chair
LSTM Research Ethics Committee

Yours sincerely,

Mr Carl Henry
LSTM Research Governance Manager
LSTM Research Governance and Ethics Office

Researching and educating to save lives
A Company Limited by Guarantee. Registered Number 83405, England and Wales. Registered Charity Number 222655.

Athena SWAN Bronze Award
INVESTORS IN PEOPLE
