# Supplementary material for: Malaria chemoprevention with monthly dihydroartemisinin-piperaquine for the post-discharge management of severe anaemia in children aged less than 5 years in Uganda and Kenya: study protocol for a multi-centre, two-arm, randomised, placebo-controlled, superiority trial
Source: Trials. 2018 Nov 6;19:610. doi: 10.1186/s13063-018-2972-1 (PMC6220494; doi:10.1186/s13063-018-2972-1)
Supplement: Supplementary file 2 — Ethics approvals: KEMRI, SOMREC, LSTM, REK vest and CDC. (ZIP 1940 kb) [file PMC_Protocol Ver.4.0_ SOMREC Approval.pdf › PMC_Protocol Ver.4.0_ SOMREC Approval.pdf]

MAKERERE UNIVERSITY

P.O. Box 7072 Kampala, Uganda
E-mail: rresearch9@gmail.

Phone: 256 414 533541
Fax: 256 414 541036/0414 532204

COLLEGE OF HEALTH SCIENCES
SCHOOL OF MEDICINE

RESEARCH ETHICS COMMITTEE

April 18, 2018

Dr. Richard Idro
Department of Paediatrics and Child Health

Category of review
[ ] Initial review
[ ] Continuing review
[X] Amendment
[ ] Termination of study
[ ] SAEs

Dear Dr. Idro,

Re: REC REF 2015-125
Title: "Malaria chemoprevention with monthly treatment with dihydroartemisinin-piperaquine for the post-discharge management of severe anaemia in children aged less than 5 years in Uganda and Kenya: A 3-year, multi-centre, parallel-group, two –arm randomized placebo controlled superiority trial" (PMC study)"

Your proposal entitled **"Malaria chemoprevention with monthly treatment with dihydroartemisinin-piperaquine for the post-discharge management of severe anaemia in children aged less than 5 years in Uganda and Kenya: A 3-year, multi-centre, parallel-group, two –arm randomized placebo controlled superiority trial" (PMC study)"** was initially reviewed and approved by the School of Medicine Research and Ethics committee on September 10th , 2015

On February 12th 2018, you requested for permission to make some modifications in the study: to re-estimate the study sample size from, 2212 to 1040 because of Limited remaining funds, and Event rate for the primary outcome (post-discharge re-admissions or deaths) that has been observed being about 3-fold higher than the event rate used in the original sample size calculations.

The committee considered these changes on 17th April, 2018. On behalf of the committee, I am glad to inform you that these changes have been approved. You may now proceed with the study. But forward regular reports on your study to the committee.

Yours sincerely,

MAKERERE UNIVERSITY
SCHOOL OF MEDICINE
APPROVED
18 APR 2018
RESEARCH & ETHICS COMMITTEE
P.O. BOX 7072, KAMPALA

Assoc. Prof. Ponsiano Ocama
Chairman School of Medicine Research & Ethics Committee
