# Supplementary material for: Malaria chemoprevention with monthly dihydroartemisinin-piperaquine for the post-discharge management of severe anaemia in children aged less than 5 years in Uganda and Kenya: study protocol for a multi-centre, two-arm, randomised, placebo-controlled, superiority trial
Source: Trials. 2018 Nov 6;19:610. doi: 10.1186/s13063-018-2972-1 (PMC6220494; doi:10.1186/s13063-018-2972-1)
Supplement: Supplementary file 2 — Ethics approvals: KEMRI, SOMREC, LSTM, REK vest and CDC. (ZIP 1940 kb) [file SOMREC 2015-125 Approval v3.0 (Amendment) 28Oct16.pdf › SOMREC 2015-125 Approval v3.0 (Amendment) 28Oct16.pdf]

MAKERERE UNIVERSITY

P.O. Box 7072 Kampala, Uganda
E-mail: rresearch9@gmail.

Phone: 256 414 533541
Fax: 256 414 541036/0414 532204

COLLEGE OF HEALTH SCIENCES
SCHOOL OF MEDICINE

RESEARCH ETHICS COMMITTEE

December 13, 2016

Dr. Richard Idro
Department of Pediatrics and Child Health

Category of review
[ ] Initial review
[ ] Continuing review
[X] Amendment
[ ] Termination of study
[ ] SAEs

Dear Dr. Idro,

Re: **REC REF 2015-125**

Title: **"Malaria chemoprevention with monthly treatment with dihydroartemisinin-piperaquine for the post-discharge management of severe anaemia in children aged less than 5 years in Uganda and Kenya: A 3 year multi-centre, parallel-group, two-arm randomized placebo controlled superiority trial" Version 3.0 dated 28th October 2016**

Your proposal entitled **"Malaria chemoprevention with monthly treatment with dihydroartemisinin-piperaquine for the post-discharge management of severe anaemia in children aged less than 5 years in Uganda and Kenya: A 3 year multi-centre, parallel-group, two-arm randomized placebo controlled superiority trial"** was initially reviewed and approved by the School of Medicine Research and Ethics committee on September 10th, 2015

On 21st November 2016, you requested for permission to make some modifications in the study and informed consent documents : to expand the study sites to include other potential hospitals and clinics in Uganda and Kenya:- Mubende regional referral hospital, Arua regional referral hospital, Iganga general hospital and Kamuli Mission hospital, study identifier added NCT02671175 for registration with the Clinicaltrials.gov, to insert date of registration in primary registry as 28th January 2016, to insert name of sponsor Liverpool School of Tropical Medicine (LSTM), to delete the secondary outcomes which were erroneously stated as the primary sponsor, to insert date of first participant enrolment as 6th May 2016, change the recruitment status from "not yet recruiting" to "recruiting", to revise the minimum weight for recruitment from >5kg to ≥5kg, to revise the strategies for retention-"*At 18 weeks after enrolment, parents or caretakers will be called or visited at home if no mobile phone contact is possible, to find out about the wellbeing of the study subject as well as remind them to come to the study clinic for the last scheduled visit at week 26*", participant information sheet for trial (consent form)

added the statement " *We will also call or visit you at 4 to 5 months to find out how your child is doing and to remind you of the last clinic visit at the end of 6 months (26 weeks),* to add malaria smears under the study design and schedule of assessment section page 17, to delete the *statement "your signature below means that you voluntarily agree to participate/have your child to participate in this research"* so as to avoid any contradictions in case a parent/guardian chooses not to provide consent for his/her child from participating in the study, to add the statement *"....or contact me on phone...* under section 8.5.6.2 line 12of the consent statement and to add the words.....or left thumbprint" to the column for signature to provide space for illiterate parents/caretakers who cannot write to append their thumbprint.

The committee considered these changes on December 13th, 2016. On behalf of the committee, I am glad to inform you that these changes have been approved. You may now proceed with the study. But forward regular reports on your study to the committee.

Yours sincerely,

MAKERERE UNIVERSITY
SCHOOL OF MEDICINE
APPROVED
13 DEC 2016
RESEARCH & ETHICS COMMITTEE
P.O.BOX 7072, KAMPALA

Assoc. Prof. Ponsiano Ocama
Chairman School of Medicine Research & Ethics Committee
